# Supplementary figures and images for: Water-soluble 4-(dimethylaminomethyl)heliomycin exerts greater antitumor effects than parental heliomycin by targeting the tNOX-SIRT1 axis and apoptosis in oral cancer cells (part 1 of 3)
Source: eLife. 2024 Apr 3;12:RP87873. doi: 10.7554/eLife.87873 (PMC10990494; doi:10.7554/eLife.87873)

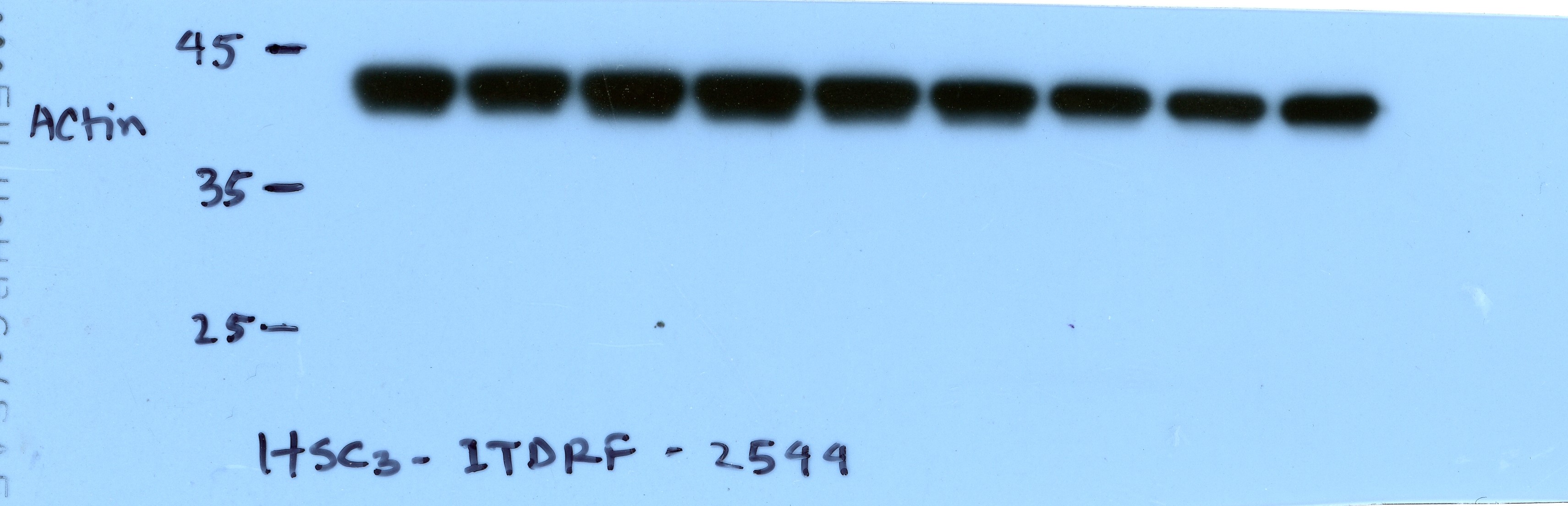

Supplement: Figure 2—source data 1. [file elife-87873-fig2-data1.zip › Figure 2-source data 1/Figure 2a (HSC-3)-Actin-4 dmH (N=1).jpg]

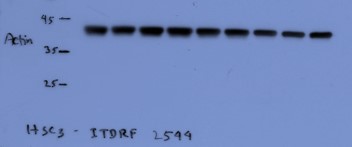

Supplement: Figure 2—source data 1. [file elife-87873-fig2-data1.zip › Figure 2-source data 1/Figure 2a (HSC-3)-Actin-4 dmH (N=2).jpg]

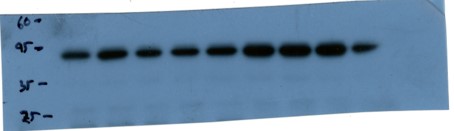

Supplement: Figure 2—source data 1. [file elife-87873-fig2-data1.zip › Figure 2-source data 1/Figure 2a (HSC-3)-Actin-4 dmH (N=3).jpg]

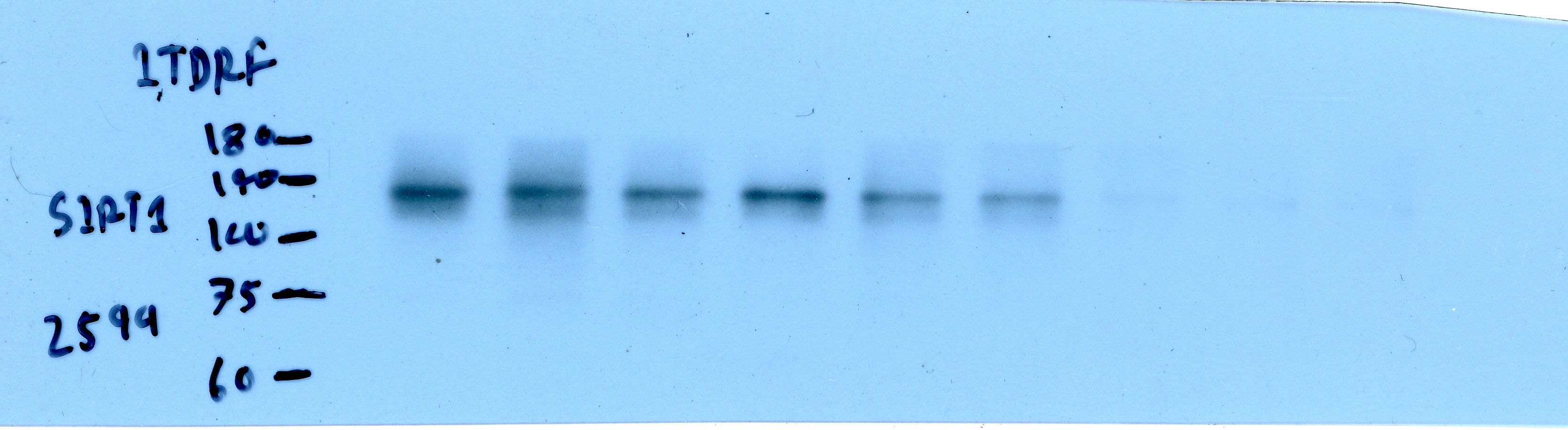

Supplement: Figure 2—source data 1. [file elife-87873-fig2-data1.zip › Figure 2-source data 1/Figure 2a (HSC-3)-SIRT1-4 dmH (N=1).jpg]

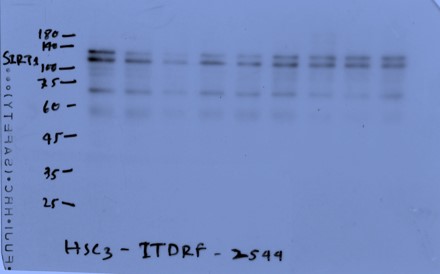

Supplement: Figure 2—source data 1. [file elife-87873-fig2-data1.zip › Figure 2-source data 1/Figure 2a (HSC-3)-SIRT1-4 dmH (N=2).jpg]

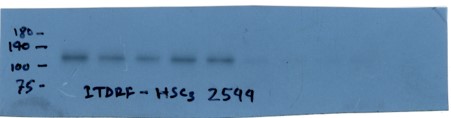

Supplement: Figure 2—source data 1. [file elife-87873-fig2-data1.zip › Figure 2-source data 1/Figure 2a (HSC-3)-SIRT1-4 dmH (N=3).jpg]

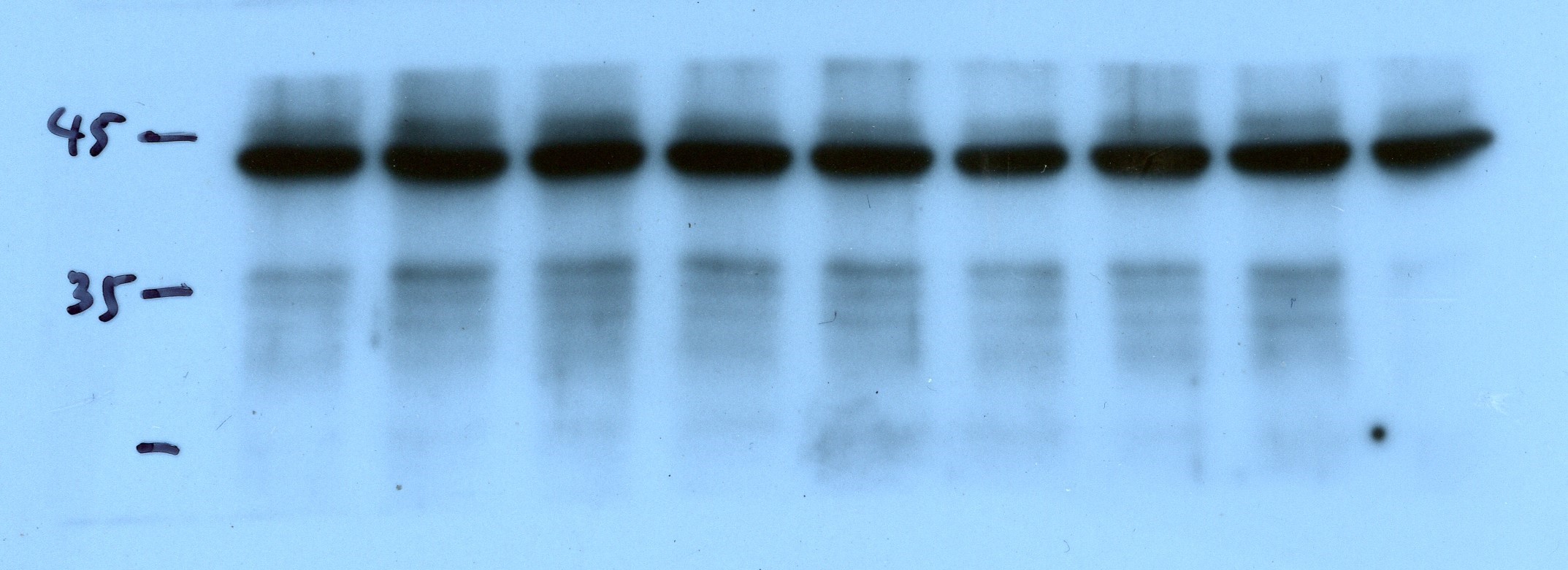

Supplement: Figure 2—source data 1. [file elife-87873-fig2-data1.zip › Figure 2-source data 1/Figure 2a (SAS)- Actin-4 dmH (N=1).jpg]

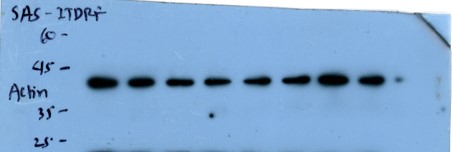

Supplement: Figure 2—source data 1. [file elife-87873-fig2-data1.zip › Figure 2-source data 1/Figure 2a (SAS)- Actin-4 dmH (N=2).jpg]

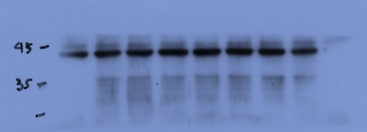

Supplement: Figure 2—source data 1. [file elife-87873-fig2-data1.zip › Figure 2-source data 1/Figure 2a (SAS)- Actin-4 dmH (N=3).jpg]

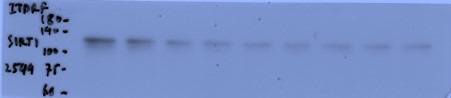

Supplement: Figure 2—source data 1. [file elife-87873-fig2-data1.zip › Figure 2-source data 1/Figure 2a (SAS)-SIRT1-4 dmH (N=1).jpg]

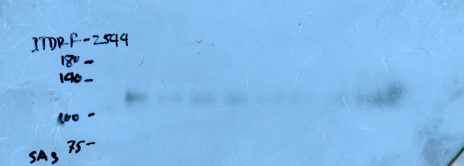

Supplement: Figure 2—source data 1. [file elife-87873-fig2-data1.zip › Figure 2-source data 1/Figure 2a (SAS)-SIRT1-4 dmH (N=2).jpg]

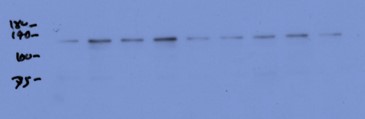

Supplement: Figure 2—source data 1. [file elife-87873-fig2-data1.zip › Figure 2-source data 1/Figure 2a (SAS)-SIRT1-4 dmH (N=3).jpg]

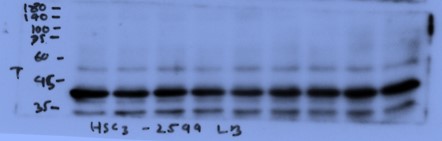

Supplement: Figure 2—source data 1. [file elife-87873-fig2-data1.zip › Figure 2-source data 1/Figure 2b (HSC-3)-Actin-4 dmH (N=1).jpg]

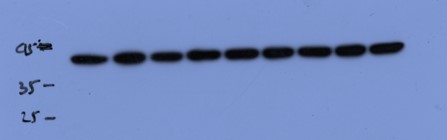

Supplement: Figure 2—source data 1. [file elife-87873-fig2-data1.zip › Figure 2-source data 1/Figure 2b (HSC-3)-Actin-4 dmH (N=2).jpg]

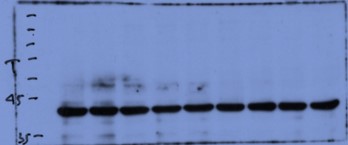

Supplement: Figure 2—source data 1. [file elife-87873-fig2-data1.zip › Figure 2-source data 1/Figure 2b (HSC-3)-Actin-4 dmH (N=3).jpg]

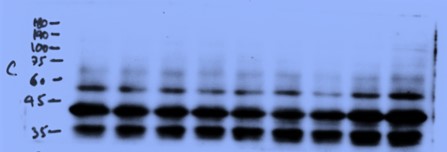

Supplement: Figure 2—source data 1. [file elife-87873-fig2-data1.zip › Figure 2-source data 1/Figure 2b (HSC-3)-Actin-H2O (N=1).jpg]

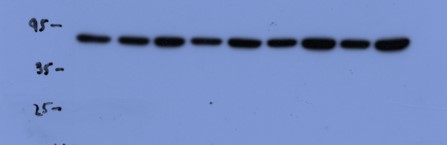

Supplement: Figure 2—source data 1. [file elife-87873-fig2-data1.zip › Figure 2-source data 1/Figure 2b (HSC-3)-Actin-H2O (N=2).jpg]

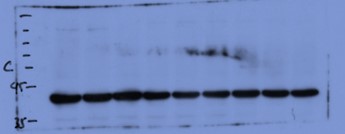

Supplement: Figure 2—source data 1. [file elife-87873-fig2-data1.zip › Figure 2-source data 1/Figure 2b (HSC-3)-Actin-H2O (N=3).jpg]

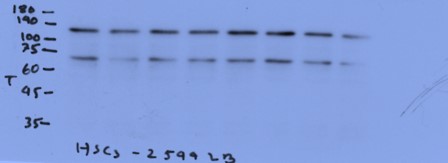

Supplement: Figure 2—source data 1. [file elife-87873-fig2-data1.zip › Figure 2-source data 1/Figure 2b (HSC-3)-SIRT1-4 dmH (N=1).jpg]

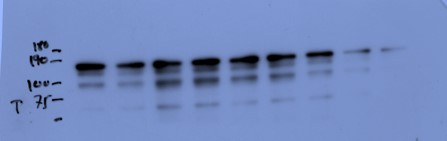

Supplement: Figure 2—source data 1. [file elife-87873-fig2-data1.zip › Figure 2-source data 1/Figure 2b (HSC-3)-SIRT1-4 dmH (N=2).jpg]

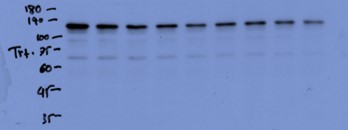

Supplement: Figure 2—source data 1. [file elife-87873-fig2-data1.zip › Figure 2-source data 1/Figure 2b (HSC-3)-SIRT1-4 dmH (N=3).jpg]

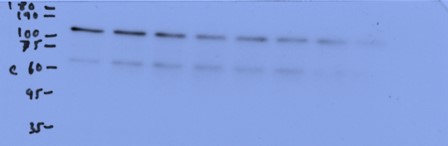

Supplement: Figure 2—source data 1. [file elife-87873-fig2-data1.zip › Figure 2-source data 1/Figure 2b (HSC-3)-SIRT1-H2O (N=1).jpg]

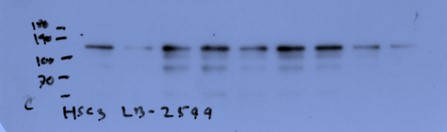

Supplement: Figure 2—source data 1. [file elife-87873-fig2-data1.zip › Figure 2-source data 1/Figure 2b (HSC-3)-SIRT1-H2O (N=2).jpg]

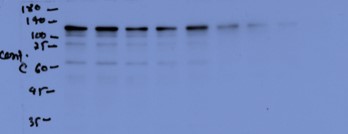

Supplement: Figure 2—source data 1. [file elife-87873-fig2-data1.zip › Figure 2-source data 1/Figure 2b (HSC-3)-SIRT1-H2O (N=3).jpg]

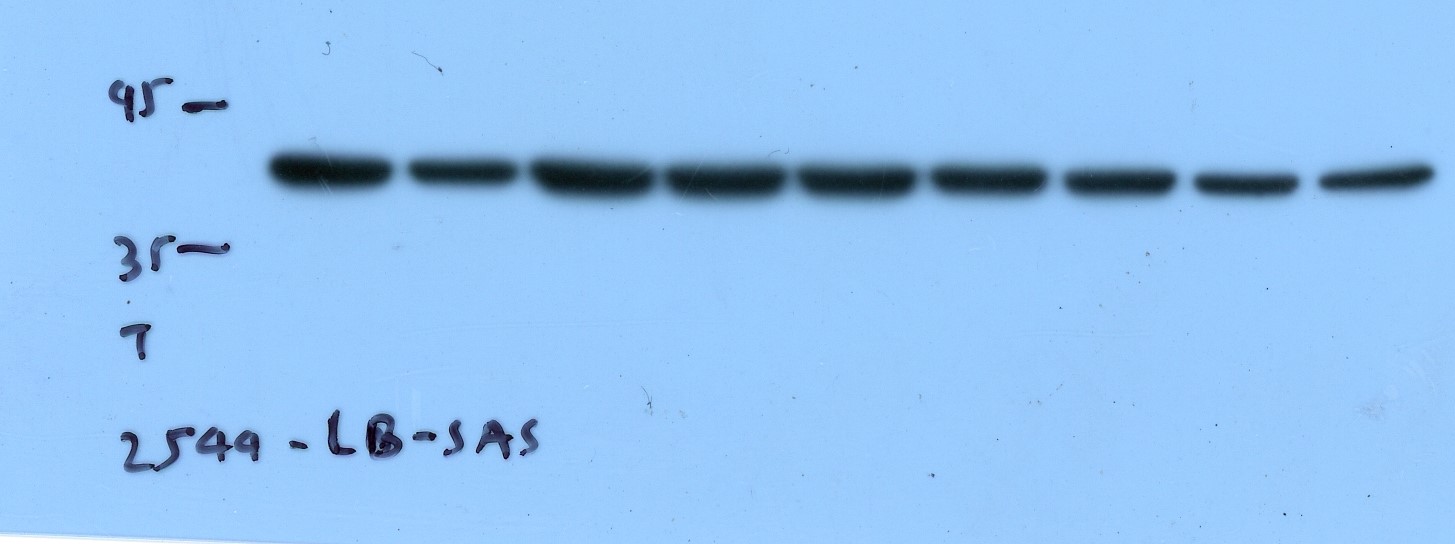

Supplement: Figure 2—source data 1. [file elife-87873-fig2-data1.zip › Figure 2-source data 1/Figure 2b (SAS)-Actin- 4 dmH (N=1).jpg]

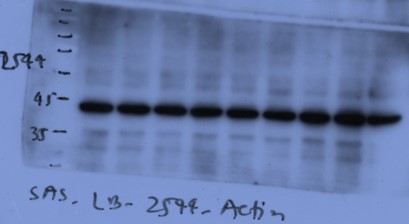

Supplement: Figure 2—source data 1. [file elife-87873-fig2-data1.zip › Figure 2-source data 1/Figure 2b (SAS)-Actin- 4 dmH (N=2).jpg]

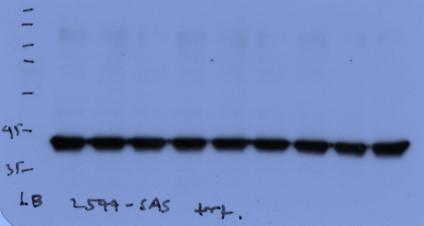

Supplement: Figure 2—source data 1. [file elife-87873-fig2-data1.zip › Figure 2-source data 1/Figure 2b (SAS)-Actin- 4 dmH (N=3).jpg]

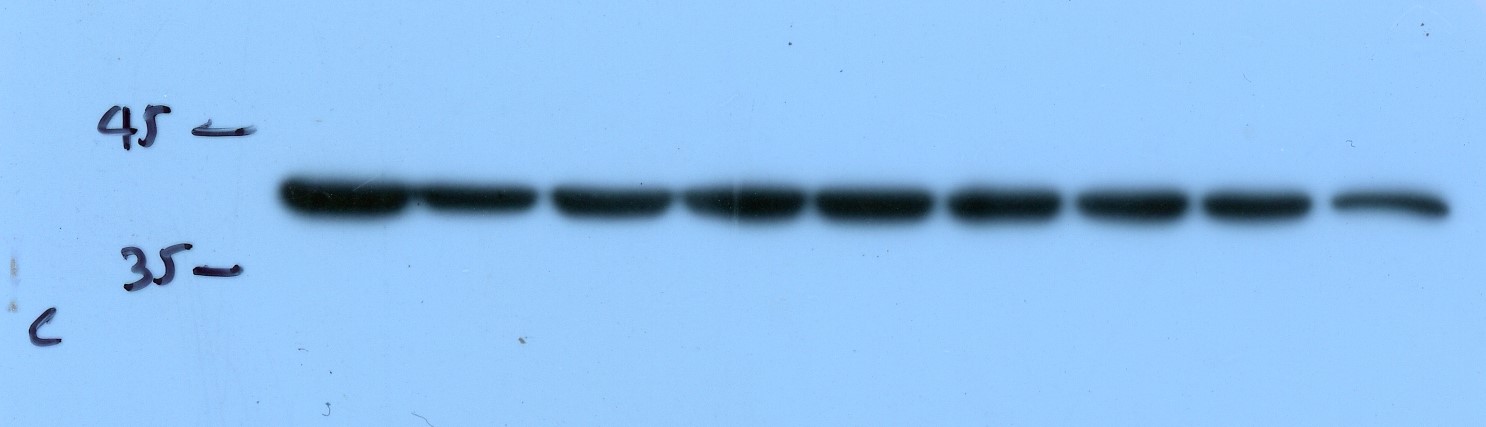

Supplement: Figure 2—source data 1. [file elife-87873-fig2-data1.zip › Figure 2-source data 1/Figure 2b (SAS)-Actin- H2O (N=1).jpg]

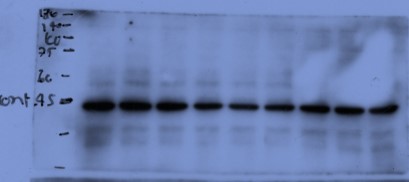

Supplement: Figure 2—source data 1. [file elife-87873-fig2-data1.zip › Figure 2-source data 1/Figure 2b (SAS)-Actin- H2O (N=2).jpg]

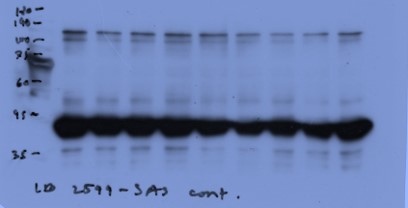

Supplement: Figure 2—source data 1. [file elife-87873-fig2-data1.zip › Figure 2-source data 1/Figure 2b (SAS)-Actin- H2O (N=3).jpg]

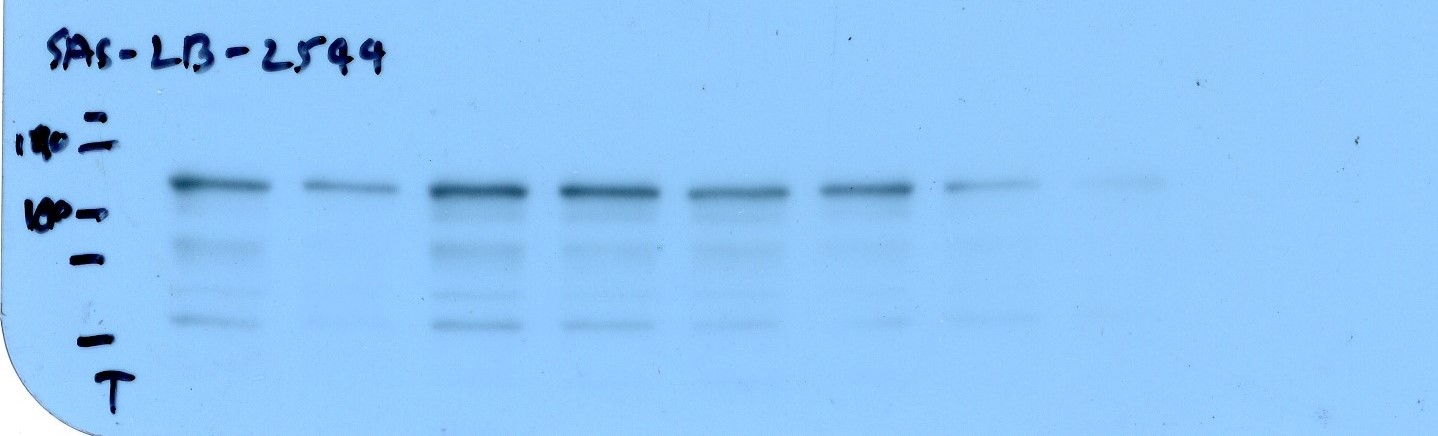

Supplement: Figure 2—source data 1. [file elife-87873-fig2-data1.zip › Figure 2-source data 1/Figure 2b (SAS)-SIRT1-4 dmH (N=1).jpg]

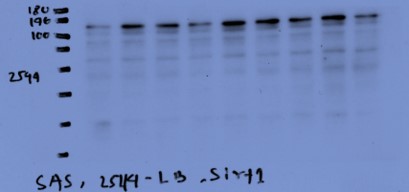

Supplement: Figure 2—source data 1. [file elife-87873-fig2-data1.zip › Figure 2-source data 1/Figure 2b (SAS)-SIRT1-4 dmH (N=2).jpg]

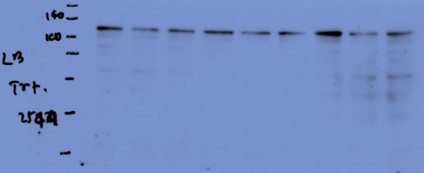

Supplement: Figure 2—source data 1. [file elife-87873-fig2-data1.zip › Figure 2-source data 1/Figure 2b (SAS)-SIRT1-4 dmH (N=3).jpg]

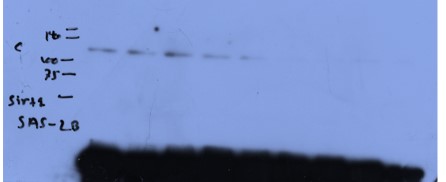

Supplement: Figure 2—source data 1. [file elife-87873-fig2-data1.zip › Figure 2-source data 1/Figure 2b (SAS)-SIRT1-H2O (N=1).jpg]

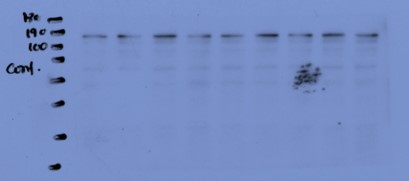

Supplement: Figure 2—source data 1. [file elife-87873-fig2-data1.zip › Figure 2-source data 1/Figure 2b (SAS)-SIRT1-H2O (N=2).jpg]

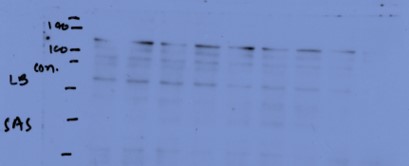

Supplement: Figure 2—source data 1. [file elife-87873-fig2-data1.zip › Figure 2-source data 1/Figure 2b (SAS)-SIRT1-H2O (N=3).jpg]

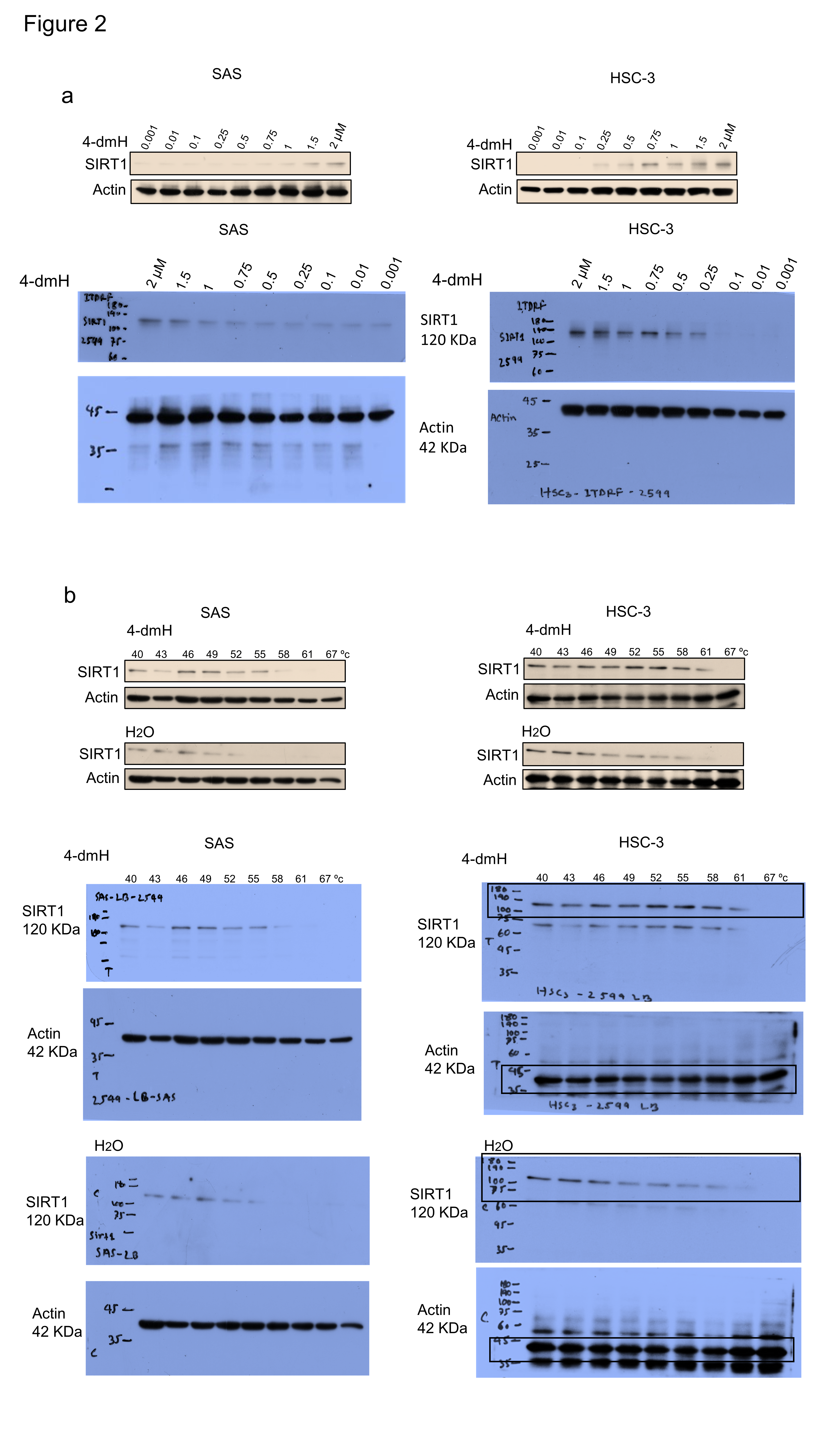

Supplement: Figure 2—source data 2. [file elife-87873-fig2-data2.zip › Figure 2-source data 2.tif]

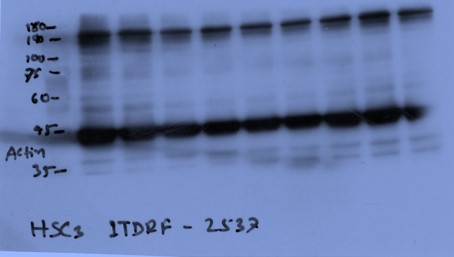

Supplement: Figure 3—source data 1. [file elife-87873-fig3-data1.zip › Figure 3-source data 1/Figure 3a (HSC-3)-Actin-Heliomycin (N=1).jpg]

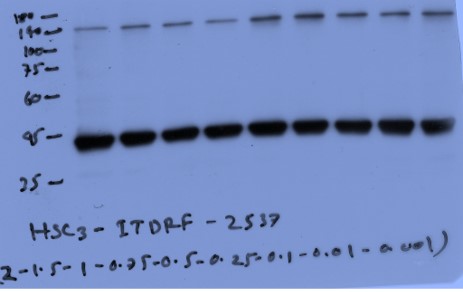

Supplement: Figure 3—source data 1. [file elife-87873-fig3-data1.zip › Figure 3-source data 1/Figure 3a (HSC-3)-Actin-Heliomycin (N=2).jpg]

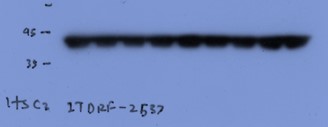

Supplement: Figure 3—source data 1. [file elife-87873-fig3-data1.zip › Figure 3-source data 1/Figure 3a (HSC-3)-Actin-Heliomycin (N=3).jpg]

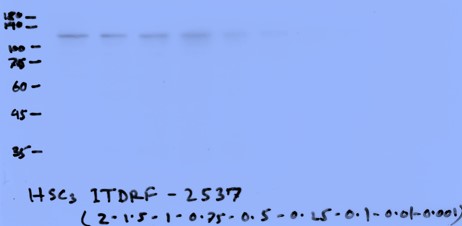

Supplement: Figure 3—source data 1. [file elife-87873-fig3-data1.zip › Figure 3-source data 1/Figure 3a (HSC-3)-SIRT1-Heliomycin (N=1).jpg]

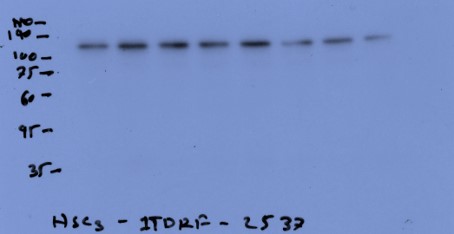

Supplement: Figure 3—source data 1. [file elife-87873-fig3-data1.zip › Figure 3-source data 1/Figure 3a (HSC-3)-SIRT1-Heliomycin (N=2).jpg]

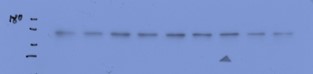

Supplement: Figure 3—source data 1. [file elife-87873-fig3-data1.zip › Figure 3-source data 1/Figure 3a (HSC-3)-SIRT1-Heliomycin (N=3).jpg]

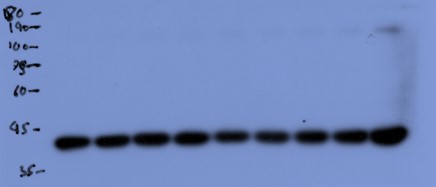

Supplement: Figure 3—source data 1. [file elife-87873-fig3-data1.zip › Figure 3-source data 1/Figure 3a (SAS)-Actin-Heliomycin (N=1).jpg]

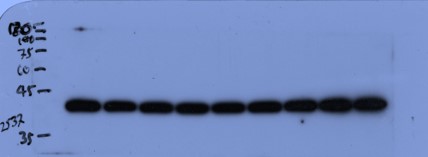

Supplement: Figure 3—source data 1. [file elife-87873-fig3-data1.zip › Figure 3-source data 1/Figure 3a (SAS)-Actin-Heliomycin (N=2).jpg]

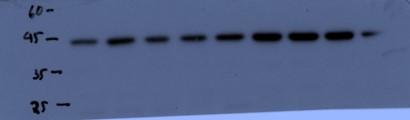

Supplement: Figure 3—source data 1. [file elife-87873-fig3-data1.zip › Figure 3-source data 1/Figure 3a (SAS)-Actin-Heliomycin (N=3).jpg]

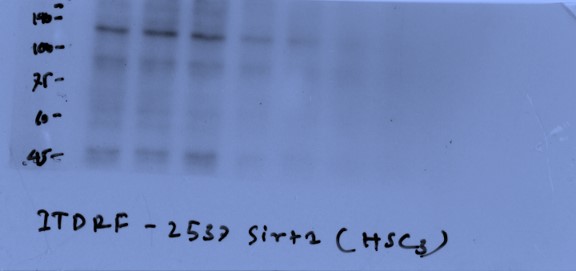

Supplement: Figure 3—source data 1. [file elife-87873-fig3-data1.zip › Figure 3-source data 1/Figure 3a (SAS)-SIRT1-Heliomycin (N=1).jpg]

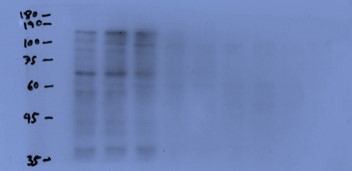

Supplement: Figure 3—source data 1. [file elife-87873-fig3-data1.zip › Figure 3-source data 1/Figure 3a (SAS)-SIRT1-Heliomycin (N=2).jpg]

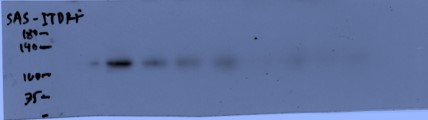

Supplement: Figure 3—source data 1. [file elife-87873-fig3-data1.zip › Figure 3-source data 1/Figure 3a (SAS)-SIRT1-Heliomycin (N=3).jpg]

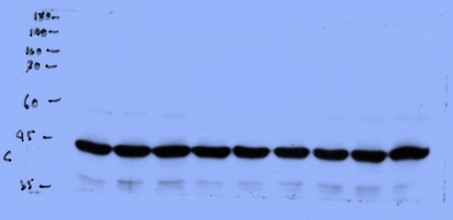

Supplement: Figure 3—source data 1. [file elife-87873-fig3-data1.zip › Figure 3-source data 1/Figure 3b (HSC-3)-Actin-DMSO (N=1).jpg]

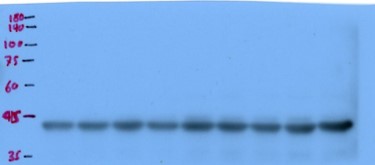

Supplement: Figure 3—source data 1. [file elife-87873-fig3-data1.zip › Figure 3-source data 1/Figure 3b (HSC-3)-Actin-DMSO (N=2).jpg]

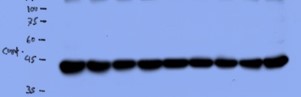

Supplement: Figure 3—source data 1. [file elife-87873-fig3-data1.zip › Figure 3-source data 1/Figure 3b (HSC-3)-Actin-DMSO (N=3).jpg]

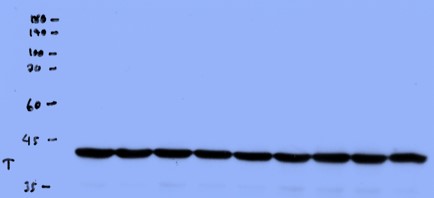

Supplement: Figure 3—source data 1. [file elife-87873-fig3-data1.zip › Figure 3-source data 1/Figure 3b (HSC-3)-Actin-Heliomycin (N=1).jpg]

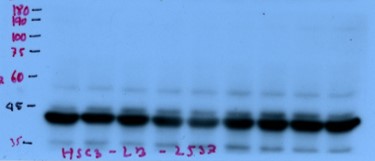

Supplement: Figure 3—source data 1. [file elife-87873-fig3-data1.zip › Figure 3-source data 1/Figure 3b (HSC-3)-Actin-Heliomycin (N=2).jpg]

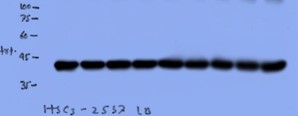

Supplement: Figure 3—source data 1. [file elife-87873-fig3-data1.zip › Figure 3-source data 1/Figure 3b (HSC-3)-Actin-Heliomycin (N=3).jpg]

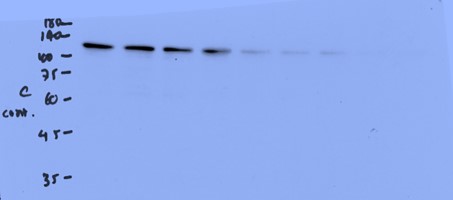

Supplement: Figure 3—source data 1. [file elife-87873-fig3-data1.zip › Figure 3-source data 1/Figure 3b (HSC-3)-SIRT1-DMSO (N=1).jpg]

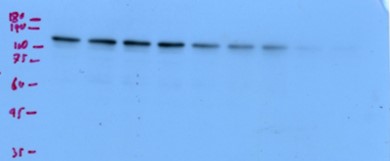

Supplement: Figure 3—source data 1. [file elife-87873-fig3-data1.zip › Figure 3-source data 1/Figure 3b (HSC-3)-SIRT1-DMSO (N=2).jpg]

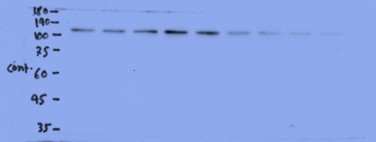

Supplement: Figure 3—source data 1. [file elife-87873-fig3-data1.zip › Figure 3-source data 1/Figure 3b (HSC-3)-SIRT1-DMSO (N=3).jpg]

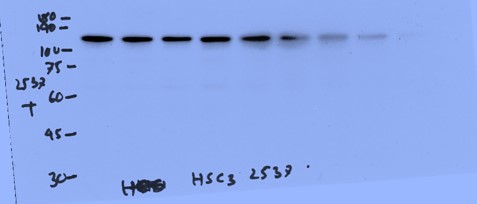

Supplement: Figure 3—source data 1. [file elife-87873-fig3-data1.zip › Figure 3-source data 1/Figure 3b (HSC-3)-SIRT1-Heliomycin (N=1).jpg]

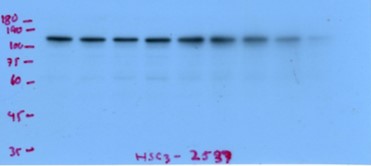

Supplement: Figure 3—source data 1. [file elife-87873-fig3-data1.zip › Figure 3-source data 1/Figure 3b (HSC-3)-SIRT1-Heliomycin (N=2).jpg]

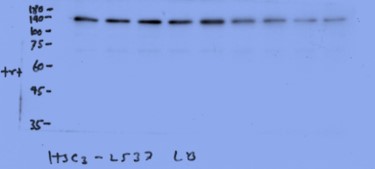

Supplement: Figure 3—source data 1. [file elife-87873-fig3-data1.zip › Figure 3-source data 1/Figure 3b (HSC-3)-SIRT1-Heliomycin (N=3).jpg]

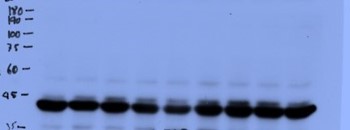

Supplement: Figure 3—source data 1. [file elife-87873-fig3-data1.zip › Figure 3-source data 1/Figure 3b (SAS)-Actin-DMS0 (N=1).jpg]

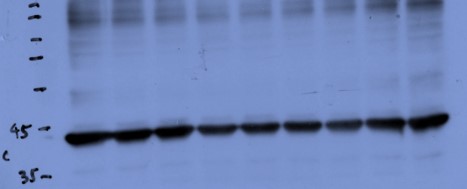

Supplement: Figure 3—source data 1. [file elife-87873-fig3-data1.zip › Figure 3-source data 1/Figure 3b (SAS)-Actin-DMS0 (N=2).jpg]

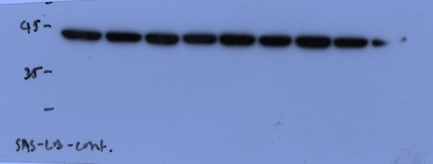

Supplement: Figure 3—source data 1. [file elife-87873-fig3-data1.zip › Figure 3-source data 1/Figure 3b (SAS)-Actin-DMS0 (N=3).jpg]

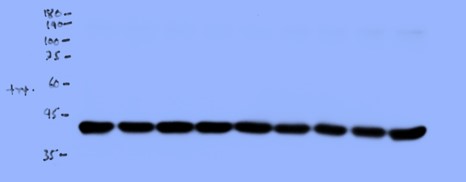

Supplement: Figure 3—source data 1. [file elife-87873-fig3-data1.zip › Figure 3-source data 1/Figure 3b (SAS)-Actin-Heliomycin (N=1).jpg]

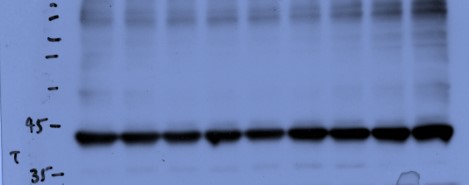

Supplement: Figure 3—source data 1. [file elife-87873-fig3-data1.zip › Figure 3-source data 1/Figure 3b (SAS)-Actin-Heliomycin (N=2).jpg]

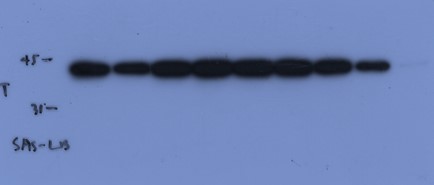

Supplement: Figure 3—source data 1. [file elife-87873-fig3-data1.zip › Figure 3-source data 1/Figure 3b (SAS)-Actin-Heliomycin (N=3).jpg]

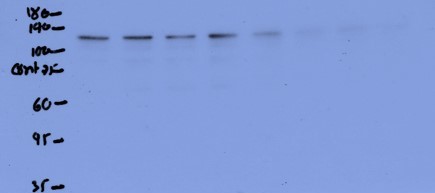

Supplement: Figure 3—source data 1. [file elife-87873-fig3-data1.zip › Figure 3-source data 1/Figure 3b (SAS)-SIRT1-DMSO (N=1).jpg]

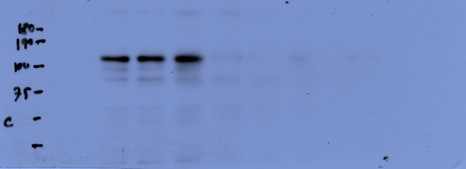

Supplement: Figure 3—source data 1. [file elife-87873-fig3-data1.zip › Figure 3-source data 1/Figure 3b (SAS)-SIRT1-DMSO (N=2).jpg]

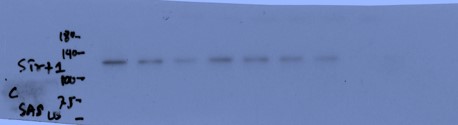

Supplement: Figure 3—source data 1. [file elife-87873-fig3-data1.zip › Figure 3-source data 1/Figure 3b (SAS)-SIRT1-DMSO (N=3).jpg]

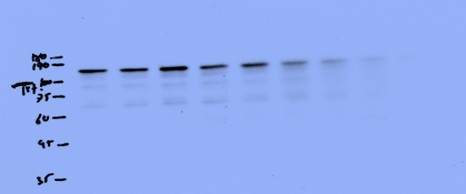

Supplement: Figure 3—source data 1. [file elife-87873-fig3-data1.zip › Figure 3-source data 1/Figure 3b (SAS)-SIRT1-Heliomycin (N=1).jpg]

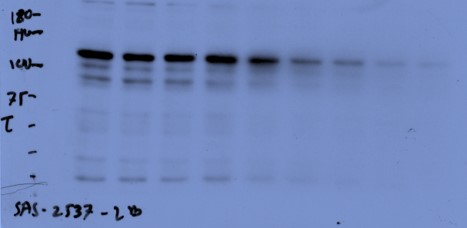

Supplement: Figure 3—source data 1. [file elife-87873-fig3-data1.zip › Figure 3-source data 1/Figure 3b (SAS)-SIRT1-Heliomycin (N=2).jpg]

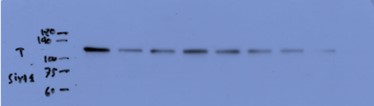

Supplement: Figure 3—source data 1. [file elife-87873-fig3-data1.zip › Figure 3-source data 1/Figure 3b (SAS)-SIRT1-Heliomycin (N=3).jpg]

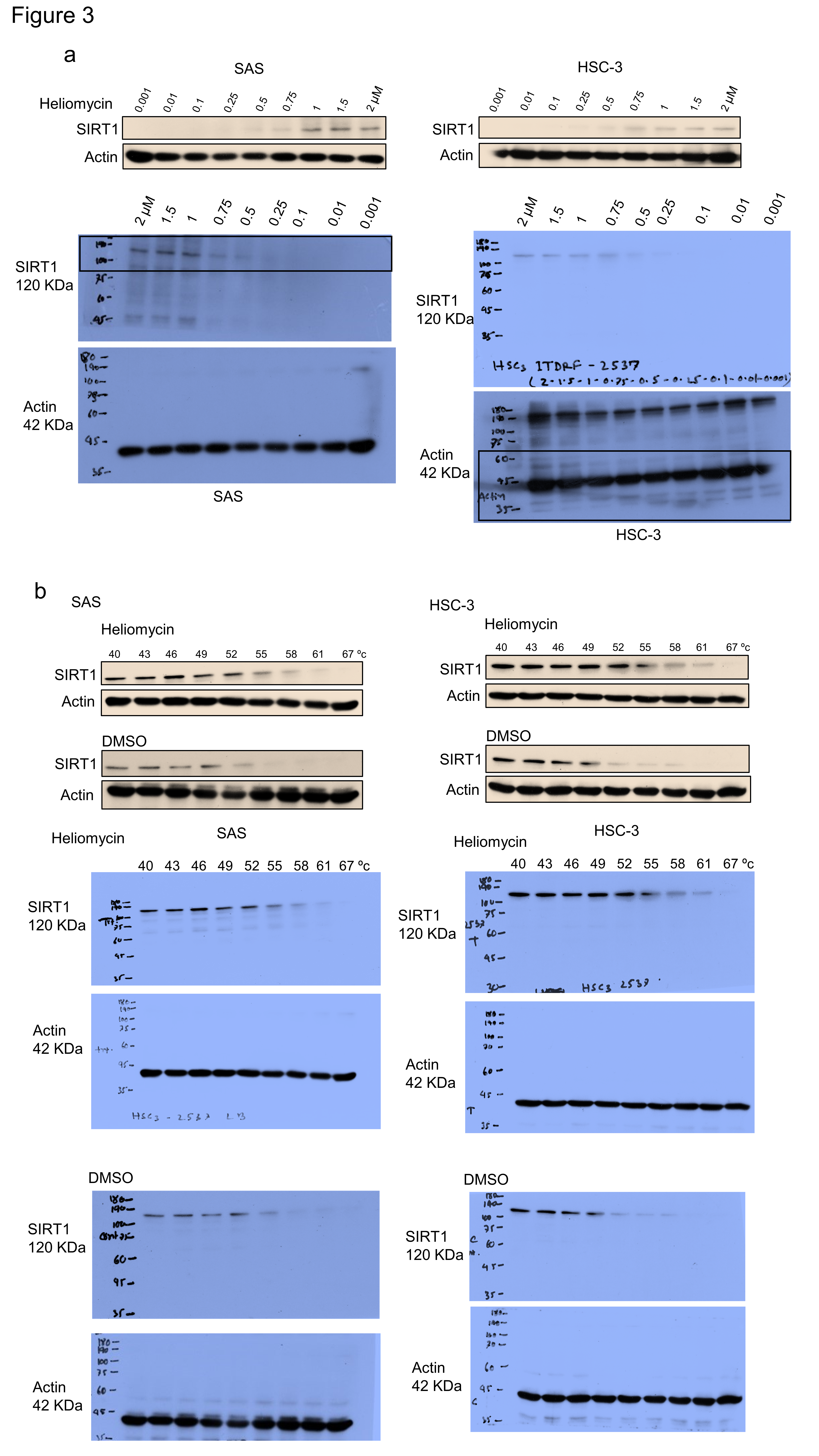

Supplement: Figure 3—source data 2. [file elife-87873-fig3-data2.zip › Figure 3-source data 2.tif]

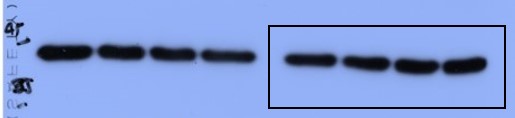

Supplement: Figure 4—source data 1. [file elife-87873-fig4-data1.zip › Figure 4-source data 1/Figure 4b (HSC-3)-Actin-4 dmH (N=1).jpg]

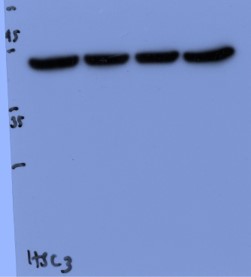

Supplement: Figure 4—source data 1. [file elife-87873-fig4-data1.zip › Figure 4-source data 1/Figure 4b (HSC-3)-Actin-4 dmH (N=2).jpg]

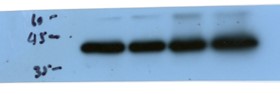

Supplement: Figure 4—source data 1. [file elife-87873-fig4-data1.zip › Figure 4-source data 1/Figure 4b (HSC-3)-Actin-4 dmH (N=3).jpg]

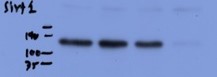

Supplement: Figure 4—source data 1. [file elife-87873-fig4-data1.zip › Figure 4-source data 1/Figure 4b (HSC-3)-SIRT1-4dmH (N=1).jpg]

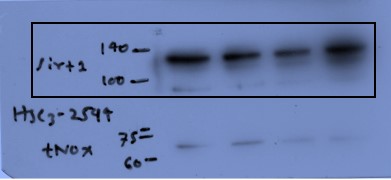

Supplement: Figure 4—source data 1. [file elife-87873-fig4-data1.zip › Figure 4-source data 1/Figure 4b (HSC-3)-SIRT1-4dmH (N=2).jpg]

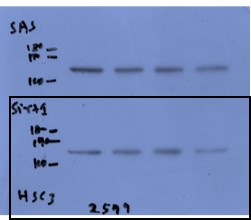

Supplement: Figure 4—source data 1. [file elife-87873-fig4-data1.zip › Figure 4-source data 1/Figure 4b (HSC-3)-SIRT1-4dmH (N=3).jpg]

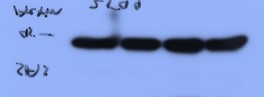

Supplement: Figure 4—source data 1. [file elife-87873-fig4-data1.zip › Figure 4-source data 1/Figure 4b (SAS)-Actin-4 dmH (N=1).jpg]

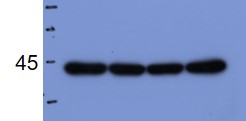

Supplement: Figure 4—source data 1. [file elife-87873-fig4-data1.zip › Figure 4-source data 1/Figure 4b (SAS)-Actin-4 dmH (N=2).jpg]

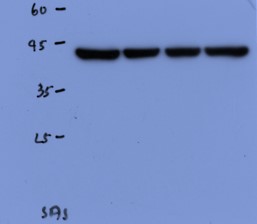

Supplement: Figure 4—source data 1. [file elife-87873-fig4-data1.zip › Figure 4-source data 1/Figure 4b (SAS)-Actin-4 dmH (N=3).jpg]

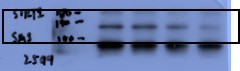

Supplement: Figure 4—source data 1. [file elife-87873-fig4-data1.zip › Figure 4-source data 1/Figure 4b (SAS)-SIRT1-4 dmH (N=1).jpg]

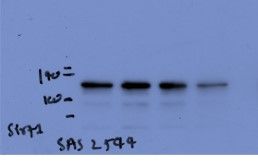

Supplement: Figure 4—source data 1. [file elife-87873-fig4-data1.zip › Figure 4-source data 1/Figure 4b (SAS)-SIRT1-4 dmH (N=2).jpg]

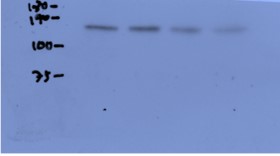

Supplement: Figure 4—source data 1. [file elife-87873-fig4-data1.zip › Figure 4-source data 1/Figure 4b (SAS)-SIRT1-4 dmH (N=3).jpg]

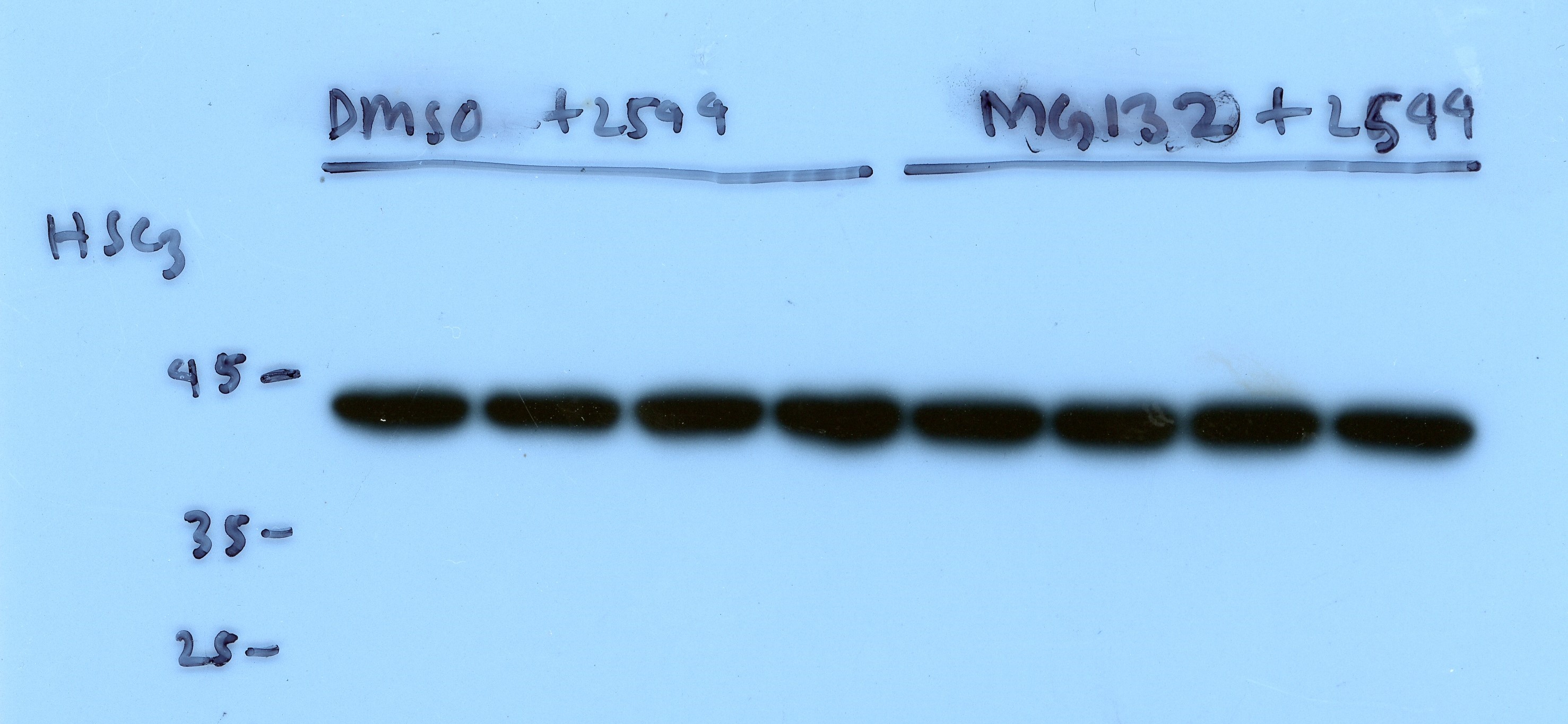

Supplement: Figure 4—source data 1. [file elife-87873-fig4-data1.zip › Figure 4-source data 1/Figure 4c (HSC-3)-Actin-4 dmH (N=1).jpg]

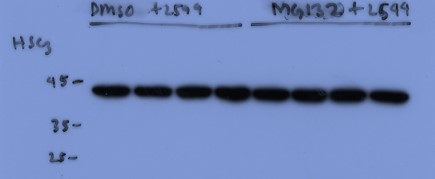

Supplement: Figure 4—source data 1. [file elife-87873-fig4-data1.zip › Figure 4-source data 1/Figure 4c (HSC-3)-Actin-4 dmH (N=2).jpg]

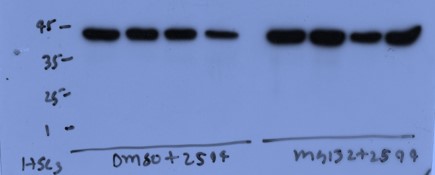

Supplement: Figure 4—source data 1. [file elife-87873-fig4-data1.zip › Figure 4-source data 1/Figure 4c (HSC-3)-Actin-4 dmH (N=3).jpg]

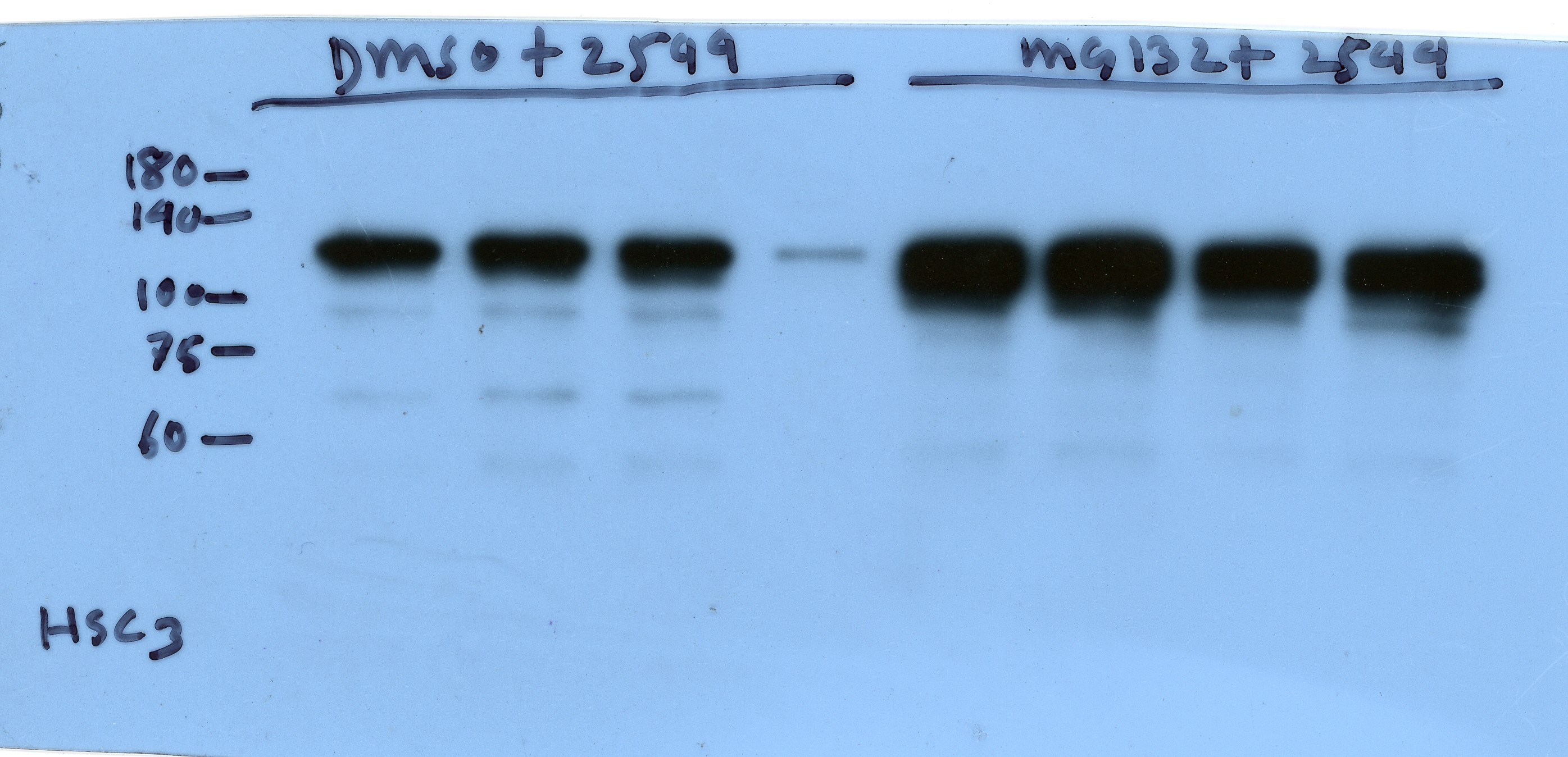

Supplement: Figure 4—source data 1. [file elife-87873-fig4-data1.zip › Figure 4-source data 1/Figure 4c (HSC-3)-SIRT1-4 dmH (N=1).jpg]

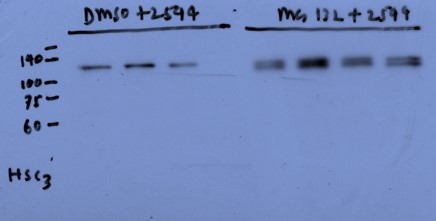

Supplement: Figure 4—source data 1. [file elife-87873-fig4-data1.zip › Figure 4-source data 1/Figure 4c (HSC-3)-SIRT1-4 dmH (N=2).jpg]

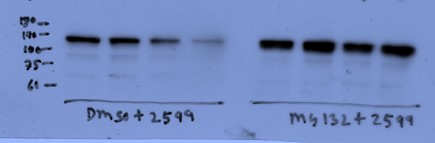

Supplement: Figure 4—source data 1. [file elife-87873-fig4-data1.zip › Figure 4-source data 1/Figure 4c (HSC-3)-SIRT1-4 dmH (N=3).jpg]

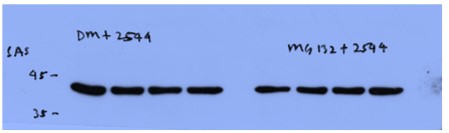

Supplement: Figure 4—source data 1. [file elife-87873-fig4-data1.zip › Figure 4-source data 1/Figure 4c (SAS)-Actin-4 dmH (N=1).jpg]

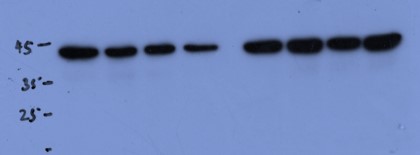

Supplement: Figure 4—source data 1. [file elife-87873-fig4-data1.zip › Figure 4-source data 1/Figure 4c (SAS)-Actin-4 dmH (N=2).jpg]

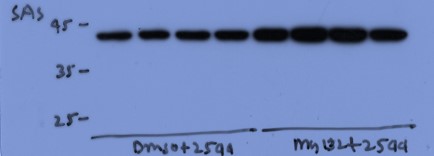

Supplement: Figure 4—source data 1. [file elife-87873-fig4-data1.zip › Figure 4-source data 1/Figure 4c (SAS)-Actin-4 dmH (N=3).jpg]

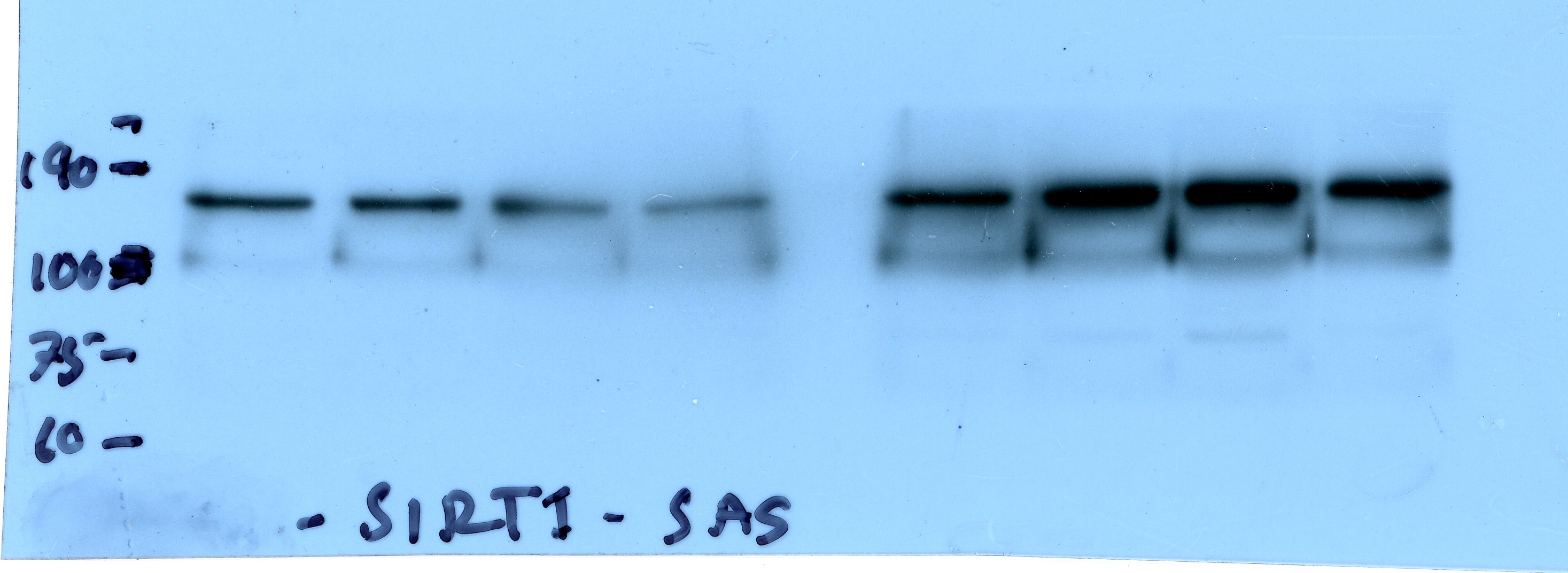

Supplement: Figure 4—source data 1. [file elife-87873-fig4-data1.zip › Figure 4-source data 1/Figure 4c (SAS)-SIRT1-4 dmH (N=1).jpg]

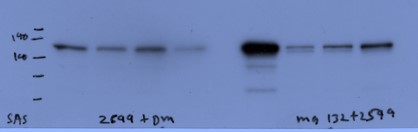

Supplement: Figure 4—source data 1. [file elife-87873-fig4-data1.zip › Figure 4-source data 1/Figure 4c (SAS)-SIRT1-4 dmH (N=2).jpg]

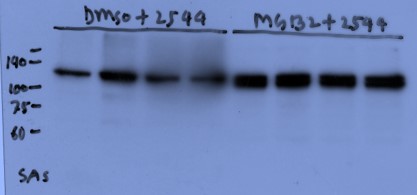

Supplement: Figure 4—source data 1. [file elife-87873-fig4-data1.zip › Figure 4-source data 1/Figure 4c (SAS)-SIRT1-4 dmH (N=3).jpg]

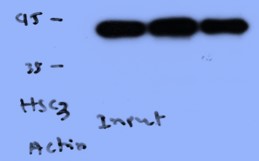

Supplement: Figure 4—source data 1. [file elife-87873-fig4-data1.zip › Figure 4-source data 1/Figure 4d (HSC-3)-Input-Actin-4 dmH.jpg]

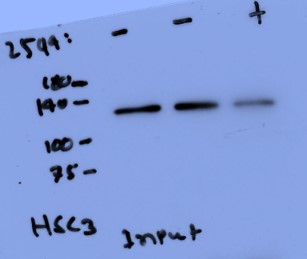

Supplement: Figure 4—source data 1. [file elife-87873-fig4-data1.zip › Figure 4-source data 1/Figure 4d (HSC-3)-Input-SIRT1-4 dmH.jpg]
