# Supplementary figures and images for: Water-soluble 4-(dimethylaminomethyl)heliomycin exerts greater antitumor effects than parental heliomycin by targeting the tNOX-SIRT1 axis and apoptosis in oral cancer cells (part 2 of 3)
Source: eLife. 2024 Apr 3;12:RP87873. doi: 10.7554/eLife.87873 (PMC10990494; doi:10.7554/eLife.87873)

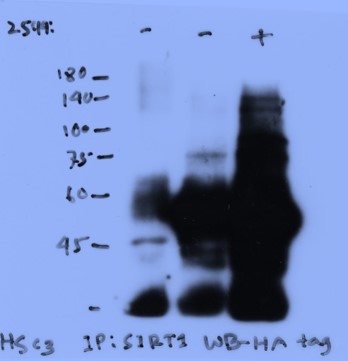

Supplement: Figure 4—source data 1. [file elife-87873-fig4-data1.zip › Figure 4-source data 1/Figure 4d (HSC-3)-IP-SIRT1,IB-HA-4 dmH.jpg]

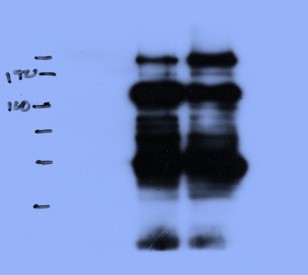

Supplement: Figure 4—source data 1. [file elife-87873-fig4-data1.zip › Figure 4-source data 1/Figure 4d (HSC-3)-IP-SIRT1,IB-SIRT1-4 dmH.jpg]

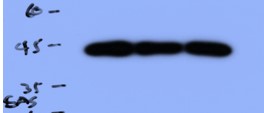

Supplement: Figure 4—source data 1. [file elife-87873-fig4-data1.zip › Figure 4-source data 1/Figure 4d (SAS)-Input-Actin-4 dmH.jpg]

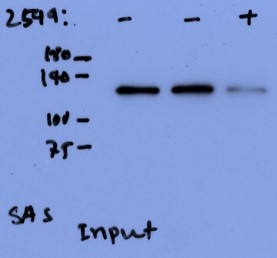

Supplement: Figure 4—source data 1. [file elife-87873-fig4-data1.zip › Figure 4-source data 1/Figure 4d (SAS)-Input-SIRT1-4 dmH.jpg]

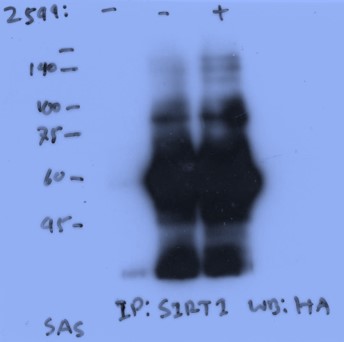

Supplement: Figure 4—source data 1. [file elife-87873-fig4-data1.zip › Figure 4-source data 1/Figure 4d (SAS)-IP-SIRT1,IB-HA-4 dmH.jpg]

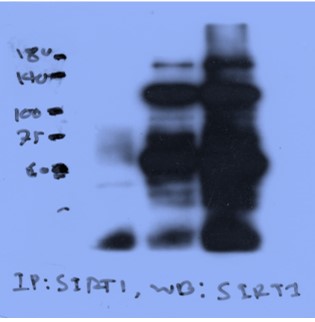

Supplement: Figure 4—source data 1. [file elife-87873-fig4-data1.zip › Figure 4-source data 1/Figure 4d (SAS)-IP-SIRT1,IB-SIRT1-4 dmH.jpg]

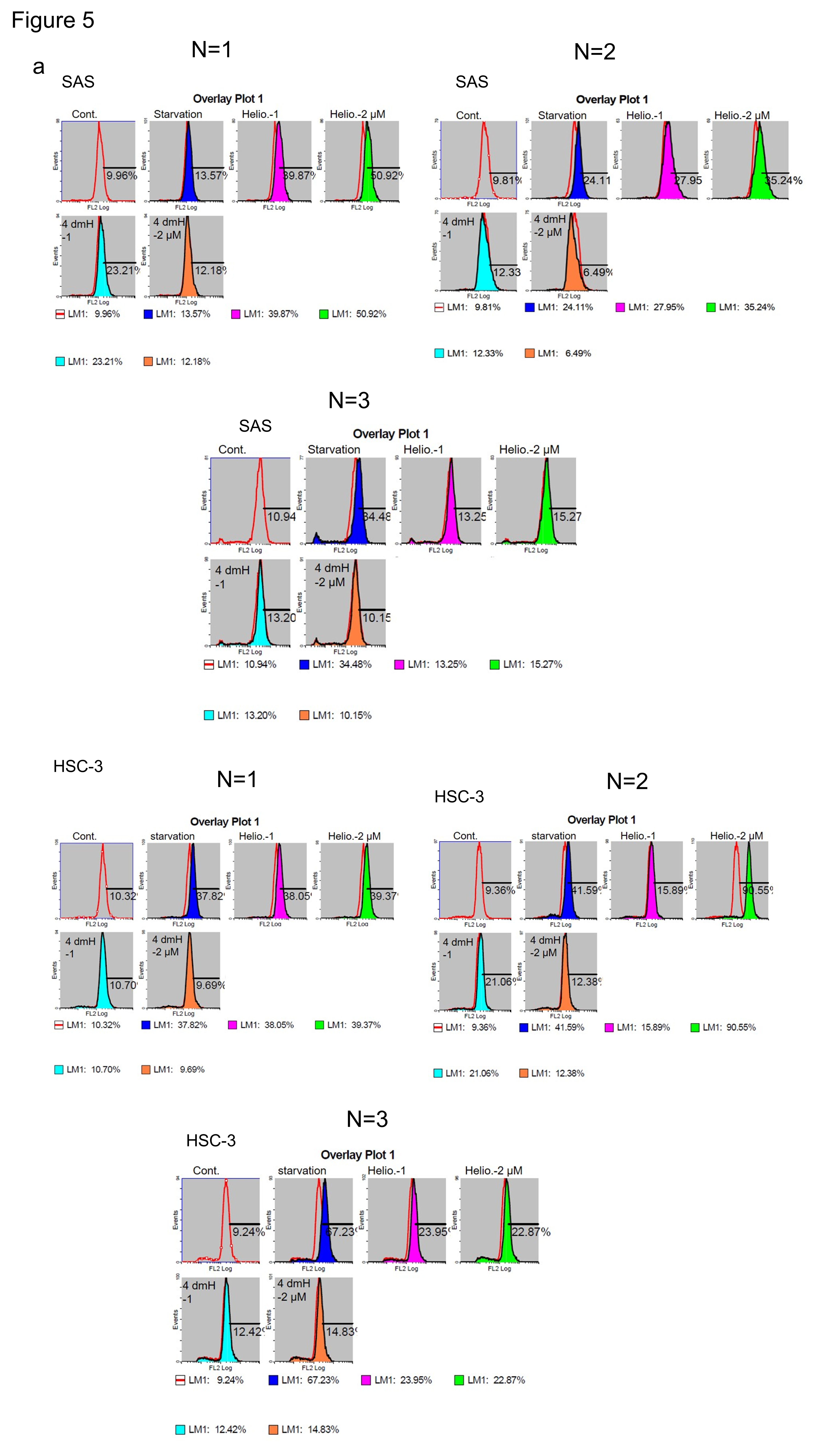

Supplement: Figure 5—source data 1. [file elife-87873-fig5-data1.zip › Figure 5-source data 1.tif]

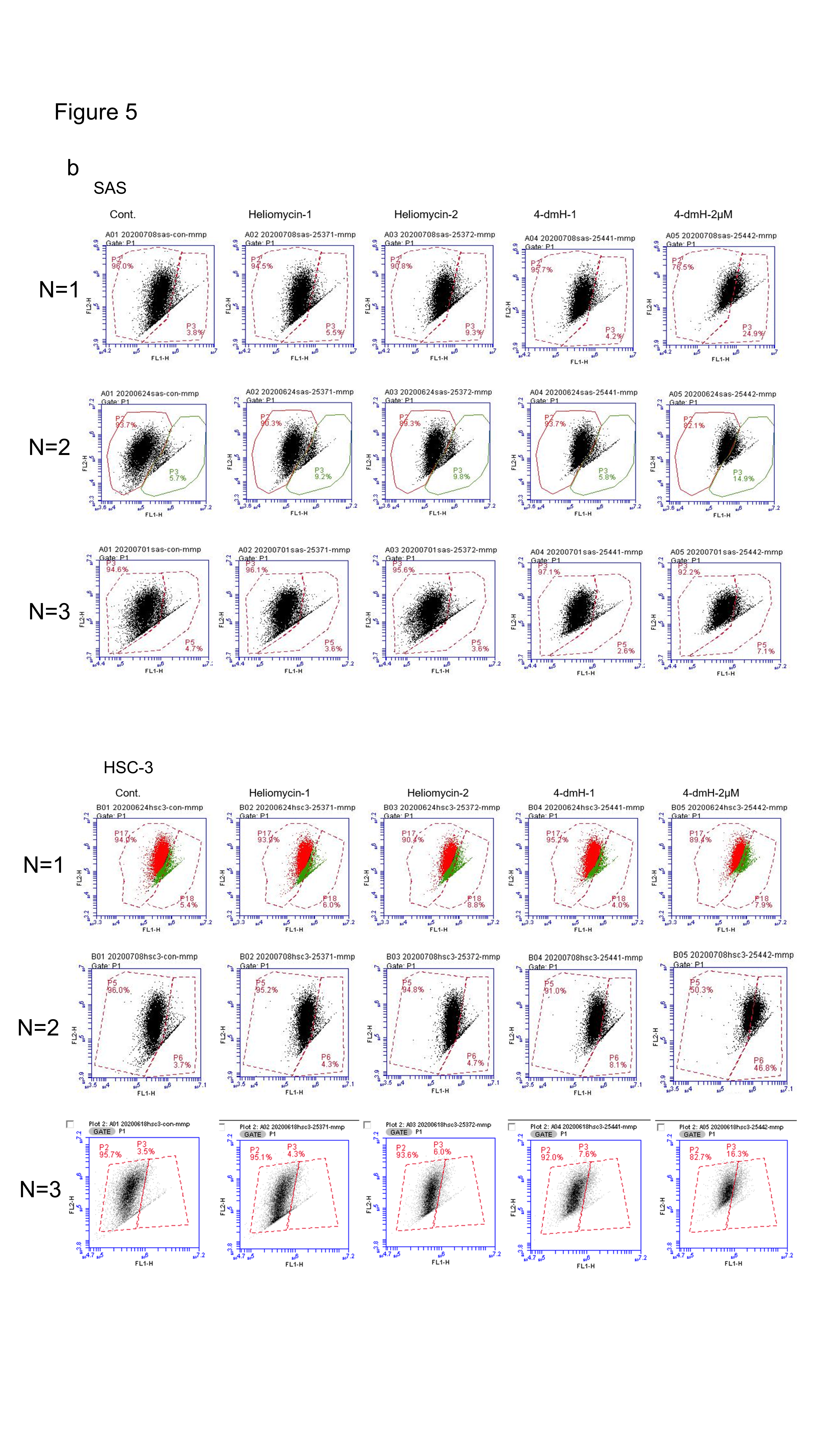

Supplement: Figure 5—source data 2. [file elife-87873-fig5-data2.zip › Figure 5-source data 2.tif]

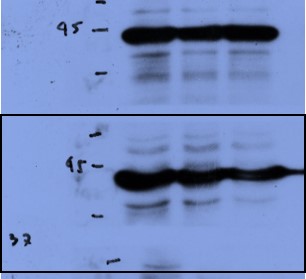

Supplement: Figure 5—source data 3. [file elife-87873-fig5-data3.zip › Figure 5-source data 3/Figure 5c (HSC-3)-Actin (2)-Heliomycin.jpg]

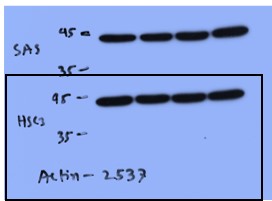

Supplement: Figure 5—source data 3. [file elife-87873-fig5-data3.zip › Figure 5-source data 3/Figure 5c (HSC-3)-Actin-Heliomycin.jpg]

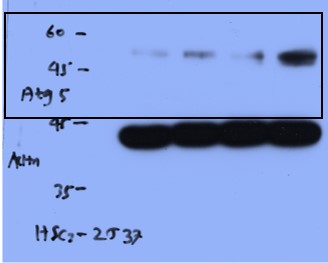

Supplement: Figure 5—source data 3. [file elife-87873-fig5-data3.zip › Figure 5-source data 3/Figure 5c (HSC-3)-Atg5-Heliomycin.jpg]

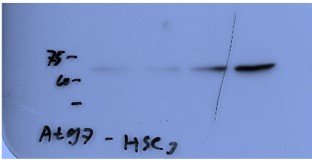

Supplement: Figure 5—source data 3. [file elife-87873-fig5-data3.zip › Figure 5-source data 3/Figure 5c (HSC-3)-Atg7-Heliomycin.jpg]

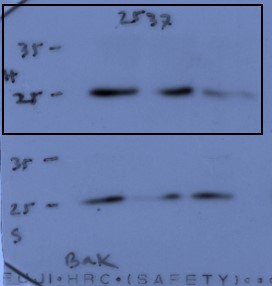

Supplement: Figure 5—source data 3. [file elife-87873-fig5-data3.zip › Figure 5-source data 3/Figure 5c (HSC-3)-Bak-Heliomycin.jpg]

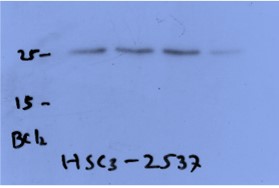

Supplement: Figure 5—source data 3. [file elife-87873-fig5-data3.zip › Figure 5-source data 3/Figure 5c (HSC-3)-Bcl2-Heliomycin.jpg]

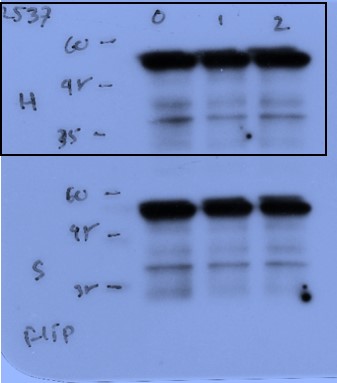

Supplement: Figure 5—source data 3. [file elife-87873-fig5-data3.zip › Figure 5-source data 3/Figure 5c (HSC-3)-Flip-Heliomycin.jpg]

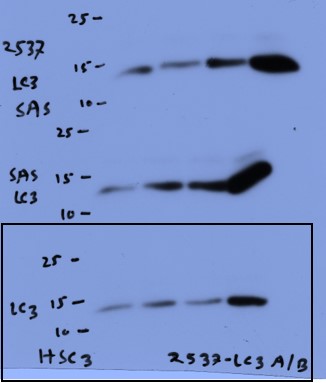

Supplement: Figure 5—source data 3. [file elife-87873-fig5-data3.zip › Figure 5-source data 3/Figure 5c (HSC-3)-LC3-Heliomycin.jpg]

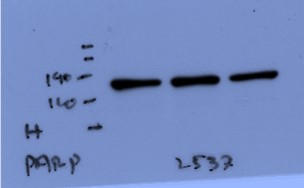

Supplement: Figure 5—source data 3. [file elife-87873-fig5-data3.zip › Figure 5-source data 3/Figure 5c (HSC-3)-PARP-Heliomycin.jpg]

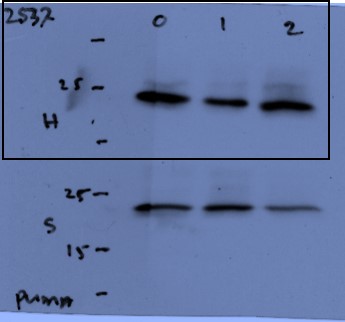

Supplement: Figure 5—source data 3. [file elife-87873-fig5-data3.zip › Figure 5-source data 3/Figure 5c (HSC-3)-Puma-Heliomycin.jpg]

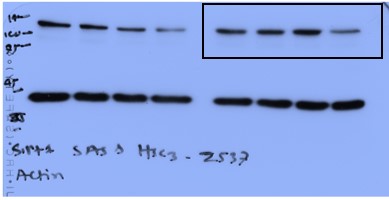

Supplement: Figure 5—source data 3. [file elife-87873-fig5-data3.zip › Figure 5-source data 3/Figure 5c (HSC-3)-SIRT1-Heliomycin.jpg]

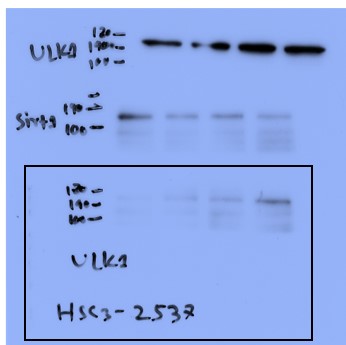

Supplement: Figure 5—source data 3. [file elife-87873-fig5-data3.zip › Figure 5-source data 3/Figure 5c (HSC-3)-ULK1-Heliomycin.jpg]

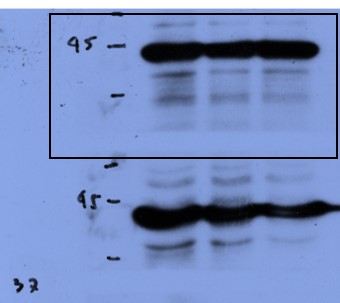

Supplement: Figure 5—source data 3. [file elife-87873-fig5-data3.zip › Figure 5-source data 3/Figure 5c (SAS)-Actin (2)-Heliomycin.jpg]

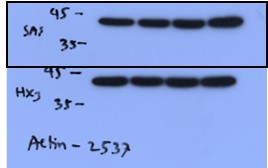

Supplement: Figure 5—source data 3. [file elife-87873-fig5-data3.zip › Figure 5-source data 3/Figure 5c (SAS)-Actin-Heliomycin.jpg]

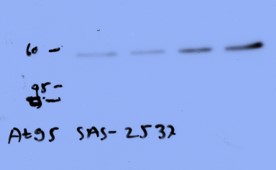

Supplement: Figure 5—source data 3. [file elife-87873-fig5-data3.zip › Figure 5-source data 3/Figure 5c (SAS)-Atg5-Heliomycin.jpg]

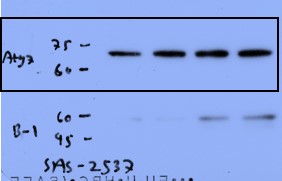

Supplement: Figure 5—source data 3. [file elife-87873-fig5-data3.zip › Figure 5-source data 3/Figure 5c (SAS)-Atg7-Heliomycin.jpg]

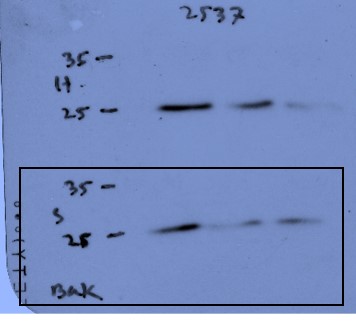

Supplement: Figure 5—source data 3. [file elife-87873-fig5-data3.zip › Figure 5-source data 3/Figure 5c (SAS)-Bak-Heliomycin.jpg]

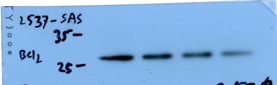

Supplement: Figure 5—source data 3. [file elife-87873-fig5-data3.zip › Figure 5-source data 3/Figure 5c (SAS)-Bcl2-Heliomycin.jpg]

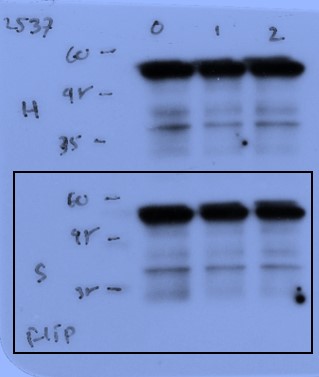

Supplement: Figure 5—source data 3. [file elife-87873-fig5-data3.zip › Figure 5-source data 3/Figure 5c (SAS)-Flip-Heliomycin.jpg]

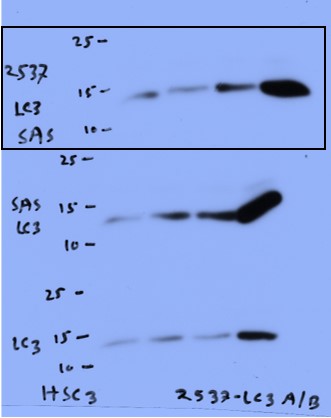

Supplement: Figure 5—source data 3. [file elife-87873-fig5-data3.zip › Figure 5-source data 3/Figure 5c (SAS)-LC3-Heliomycin.jpg]

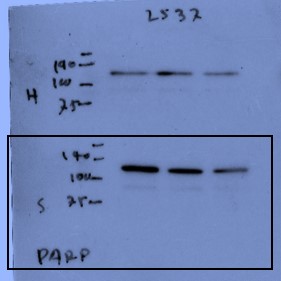

Supplement: Figure 5—source data 3. [file elife-87873-fig5-data3.zip › Figure 5-source data 3/Figure 5c (SAS)-PARP-Heliomycin.jpg]

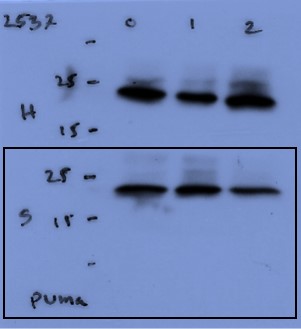

Supplement: Figure 5—source data 3. [file elife-87873-fig5-data3.zip › Figure 5-source data 3/Figure 5c (SAS)-Puma-Heliomycin.jpg]

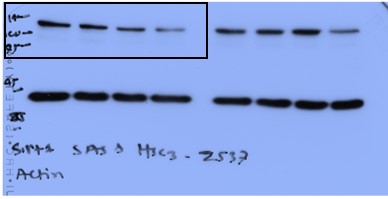

Supplement: Figure 5—source data 3. [file elife-87873-fig5-data3.zip › Figure 5-source data 3/Figure 5c (SAS)-SIRT1-Heliomycin.jpg]

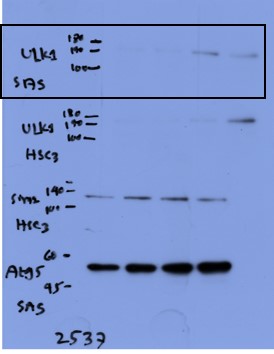

Supplement: Figure 5—source data 3. [file elife-87873-fig5-data3.zip › Figure 5-source data 3/Figure 5c (SAS)-ULK1-Heliomycin.jpg]

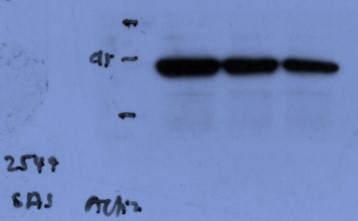

Supplement: Figure 5—source data 3. [file elife-87873-fig5-data3.zip › Figure 5-source data 3/Figure 5d ( SAS)-Actin (2)-4 dmH.jpg]

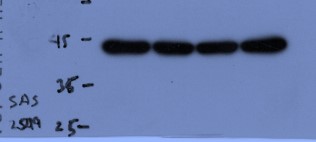

Supplement: Figure 5—source data 3. [file elife-87873-fig5-data3.zip › Figure 5-source data 3/Figure 5d ( SAS)-Actin-4 dmH.jpg]

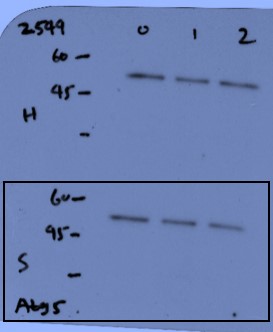

Supplement: Figure 5—source data 3. [file elife-87873-fig5-data3.zip › Figure 5-source data 3/Figure 5d ( SAS)-Atg5-4 dmH.jpg]

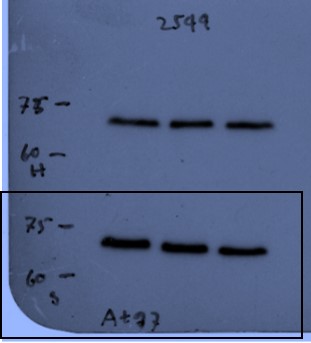

Supplement: Figure 5—source data 3. [file elife-87873-fig5-data3.zip › Figure 5-source data 3/Figure 5d ( SAS)-Atg7-4 dmH.jpg]

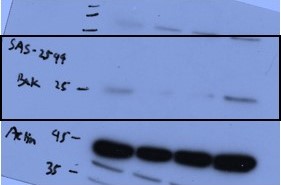

Supplement: Figure 5—source data 3. [file elife-87873-fig5-data3.zip › Figure 5-source data 3/Figure 5d ( SAS)-Bak-4 dmH.jpg]

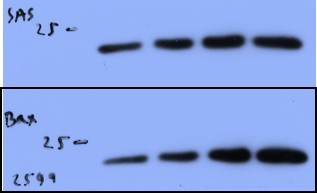

Supplement: Figure 5—source data 3. [file elife-87873-fig5-data3.zip › Figure 5-source data 3/Figure 5d ( SAS)-Bax-4 dmH.jpg]

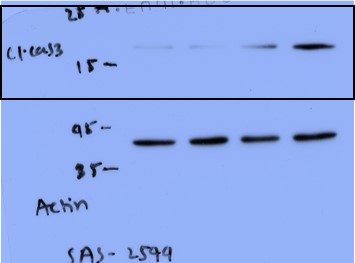

Supplement: Figure 5—source data 3. [file elife-87873-fig5-data3.zip › Figure 5-source data 3/Figure 5d ( SAS)-cleaved caspase 3-4 dmH.jpg]

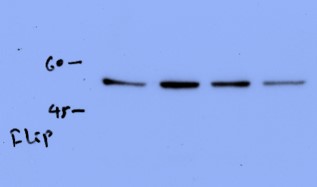

Supplement: Figure 5—source data 3. [file elife-87873-fig5-data3.zip › Figure 5-source data 3/Figure 5d ( SAS)-Flip-4 dmH.jpg]

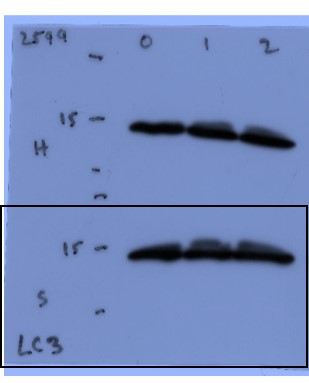

Supplement: Figure 5—source data 3. [file elife-87873-fig5-data3.zip › Figure 5-source data 3/Figure 5d ( SAS)-LC3-4 dmH.jpg]

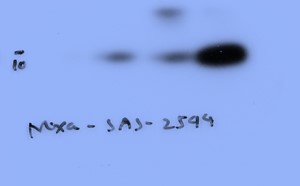

Supplement: Figure 5—source data 3. [file elife-87873-fig5-data3.zip › Figure 5-source data 3/Figure 5d ( SAS)-Noxa-4 dmH.jpg]

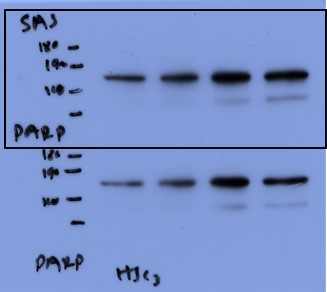

Supplement: Figure 5—source data 3. [file elife-87873-fig5-data3.zip › Figure 5-source data 3/Figure 5d ( SAS)-PARP-4 dmH.jpg]

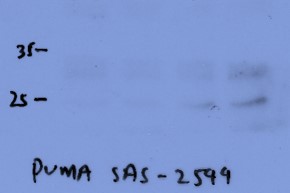

Supplement: Figure 5—source data 3. [file elife-87873-fig5-data3.zip › Figure 5-source data 3/Figure 5d ( SAS)-Puma-4 dmH.jpg]

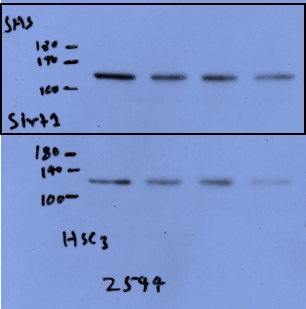

Supplement: Figure 5—source data 3. [file elife-87873-fig5-data3.zip › Figure 5-source data 3/Figure 5d ( SAS)-SIRT1-4 dmH.jpg]

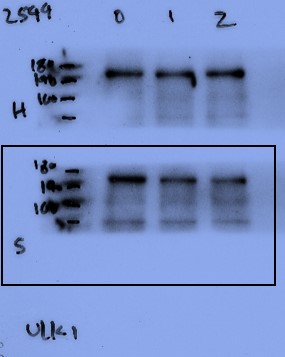

Supplement: Figure 5—source data 3. [file elife-87873-fig5-data3.zip › Figure 5-source data 3/Figure 5d ( SAS)-ULK1-4 dmH.jpg]

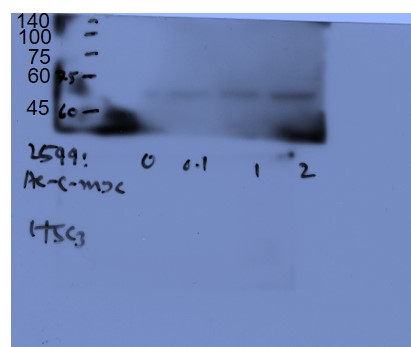

Supplement: Figure 5—source data 3. [file elife-87873-fig5-data3.zip › Figure 5-source data 3/Figure 5d(HSC-3)-Ac-c-Myc-4 dmH.jpg]

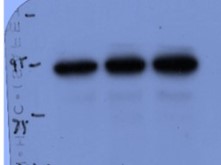

Supplement: Figure 5—source data 3. [file elife-87873-fig5-data3.zip › Figure 5-source data 3/Figure 5d(HSC-3)-Actin (2)-4 dmH.jpg]

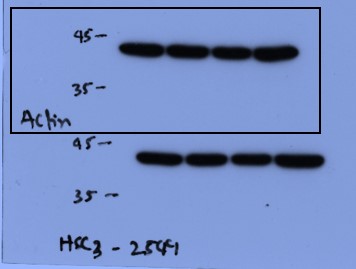

Supplement: Figure 5—source data 3. [file elife-87873-fig5-data3.zip › Figure 5-source data 3/Figure 5d(HSC-3)-Actin-4 dmH.jpg]

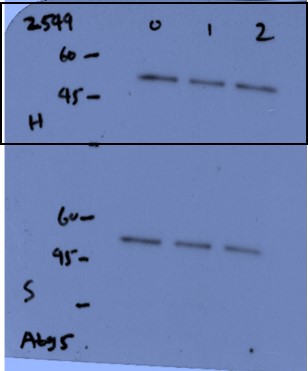

Supplement: Figure 5—source data 3. [file elife-87873-fig5-data3.zip › Figure 5-source data 3/Figure 5d(HSC-3)-Atg5-4 dmH.jpg]

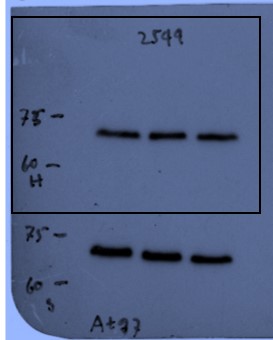

Supplement: Figure 5—source data 3. [file elife-87873-fig5-data3.zip › Figure 5-source data 3/Figure 5d(HSC-3)-Atg7-4 dmH.jpg]

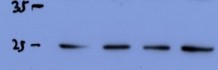

Supplement: Figure 5—source data 3. [file elife-87873-fig5-data3.zip › Figure 5-source data 3/Figure 5d(HSC-3)-Bak-4 dmH.jpg]

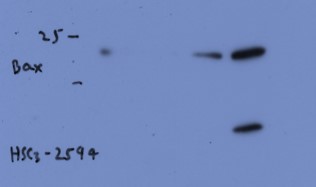

Supplement: Figure 5—source data 3. [file elife-87873-fig5-data3.zip › Figure 5-source data 3/Figure 5d(HSC-3)-Bax-4 dmH.jpg]

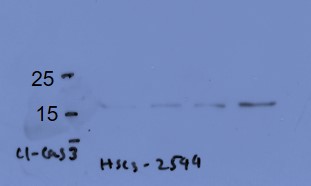

Supplement: Figure 5—source data 3. [file elife-87873-fig5-data3.zip › Figure 5-source data 3/Figure 5d(HSC-3)-cleaved caspase 3-4 dmH.jpg]

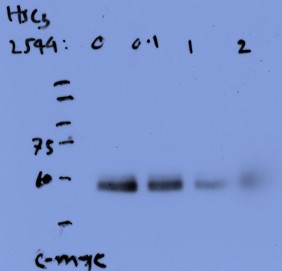

Supplement: Figure 5—source data 3. [file elife-87873-fig5-data3.zip › Figure 5-source data 3/Figure 5d(HSC-3)-c-Myc-4 dmH.jpg]

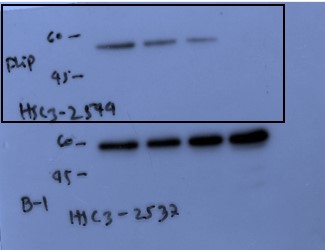

Supplement: Figure 5—source data 3. [file elife-87873-fig5-data3.zip › Figure 5-source data 3/Figure 5d(HSC-3)-Flip-4 dmH.jpg]

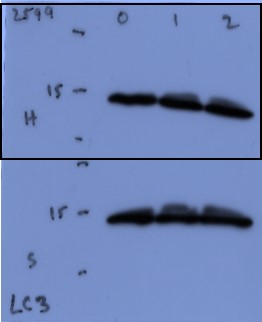

Supplement: Figure 5—source data 3. [file elife-87873-fig5-data3.zip › Figure 5-source data 3/Figure 5d(HSC-3)-LC3-4 dmH.jpg]

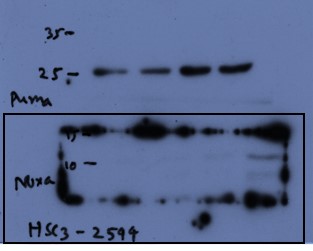

Supplement: Figure 5—source data 3. [file elife-87873-fig5-data3.zip › Figure 5-source data 3/Figure 5d(HSC-3)-Noxa-4 dmH.jpg]

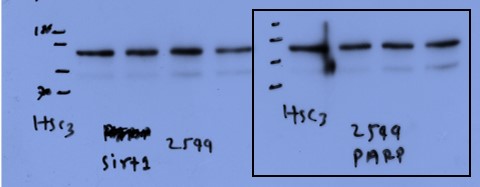

Supplement: Figure 5—source data 3. [file elife-87873-fig5-data3.zip › Figure 5-source data 3/Figure 5d(HSC-3)-PARP-4 dmH.jpg]

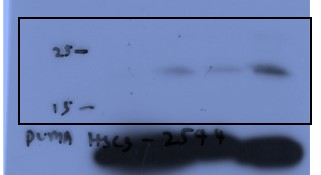

Supplement: Figure 5—source data 3. [file elife-87873-fig5-data3.zip › Figure 5-source data 3/Figure 5d(HSC-3)-Puma-4 dmH.jpg]

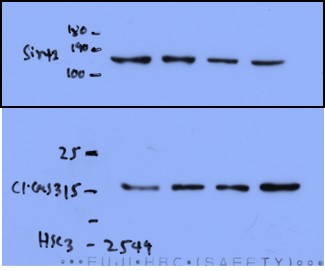

Supplement: Figure 5—source data 3. [file elife-87873-fig5-data3.zip › Figure 5-source data 3/Figure 5d(HSC-3)-SIRT1-4 dmH.jpg]

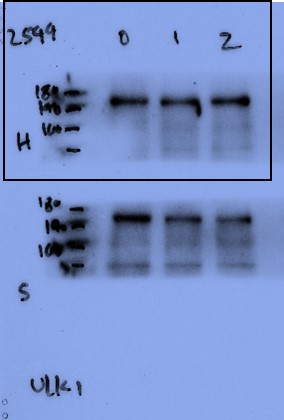

Supplement: Figure 5—source data 3. [file elife-87873-fig5-data3.zip › Figure 5-source data 3/Figure 5d(HSC-3)-ULK1-4 dmH.jpg]

C

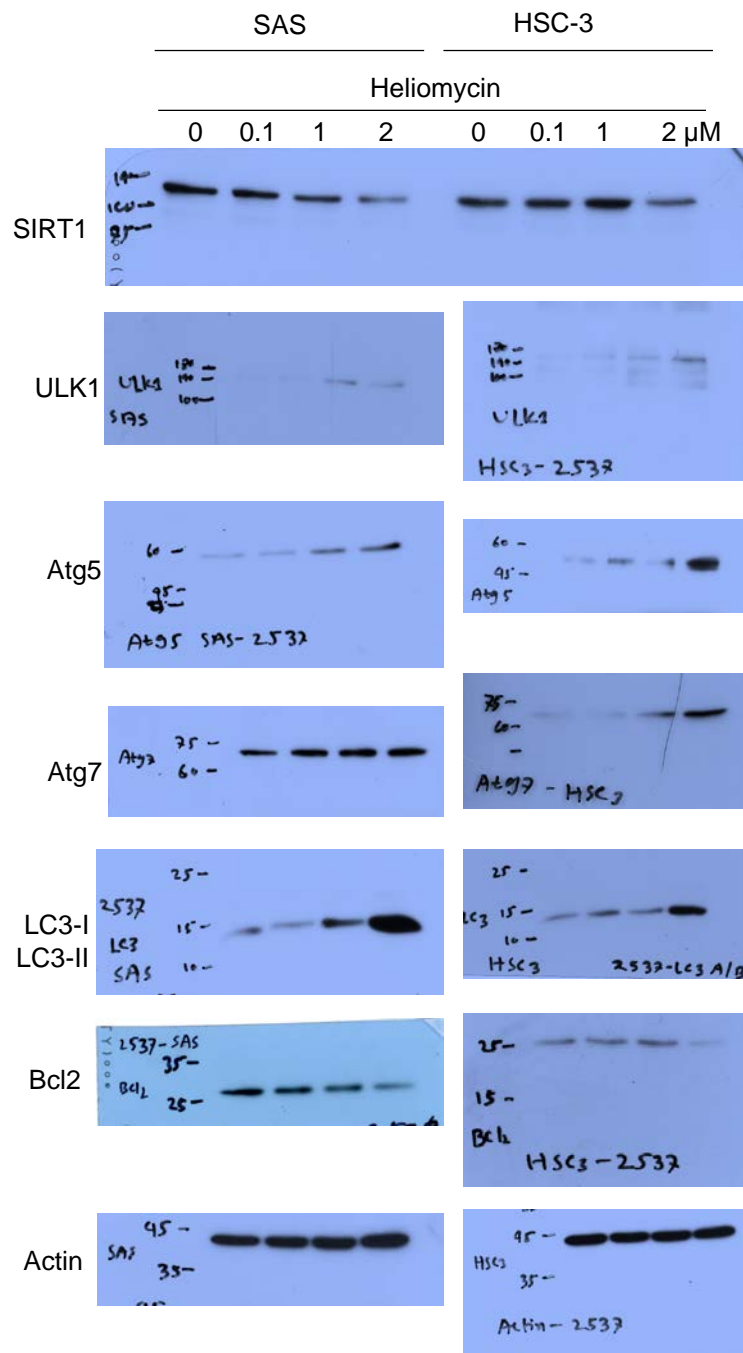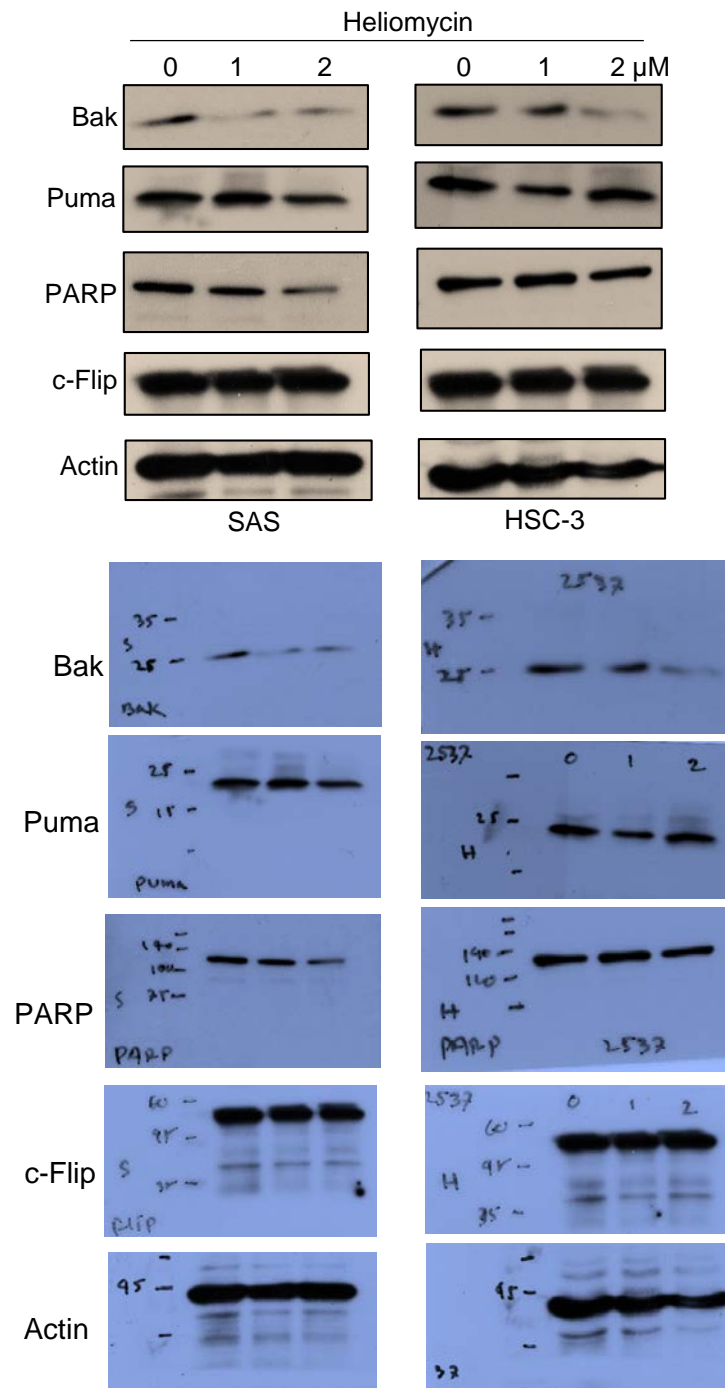

Figure 5

d

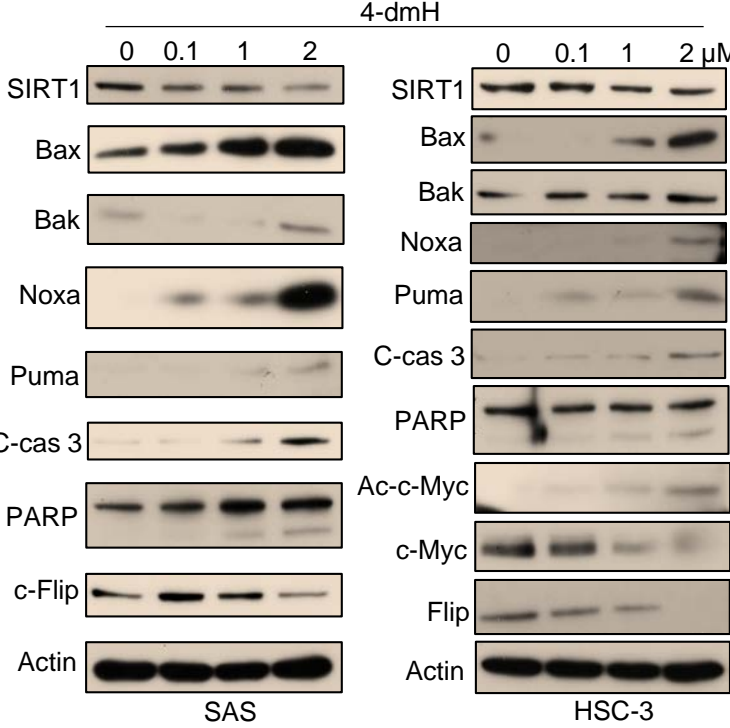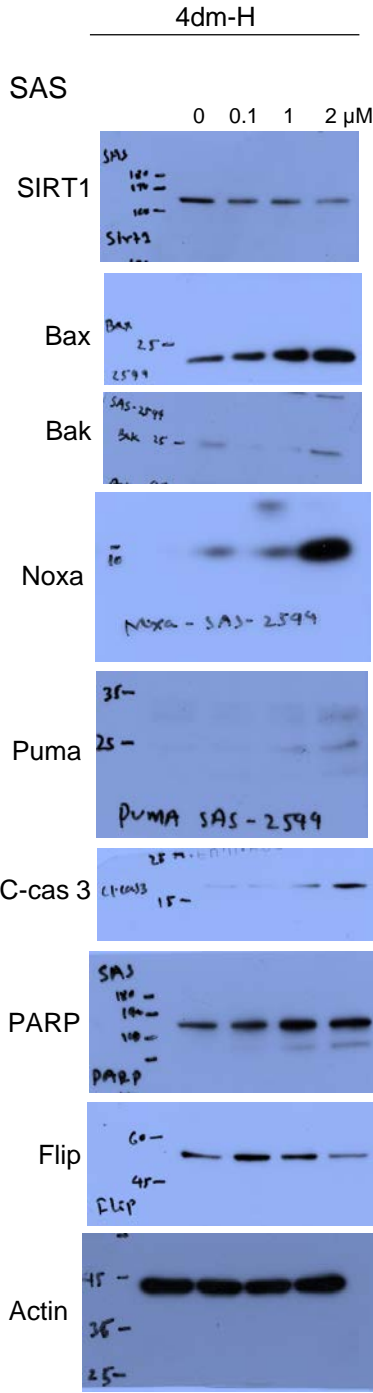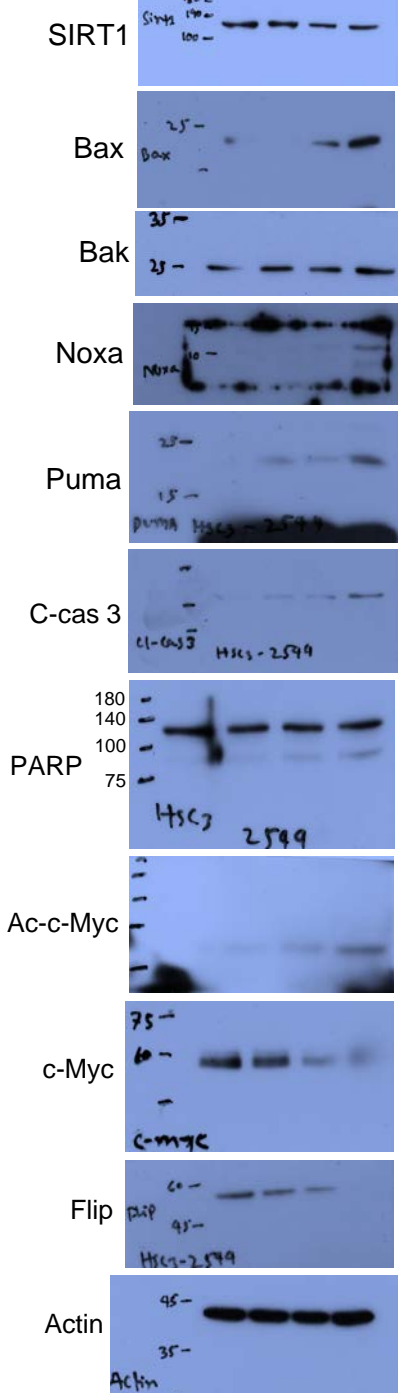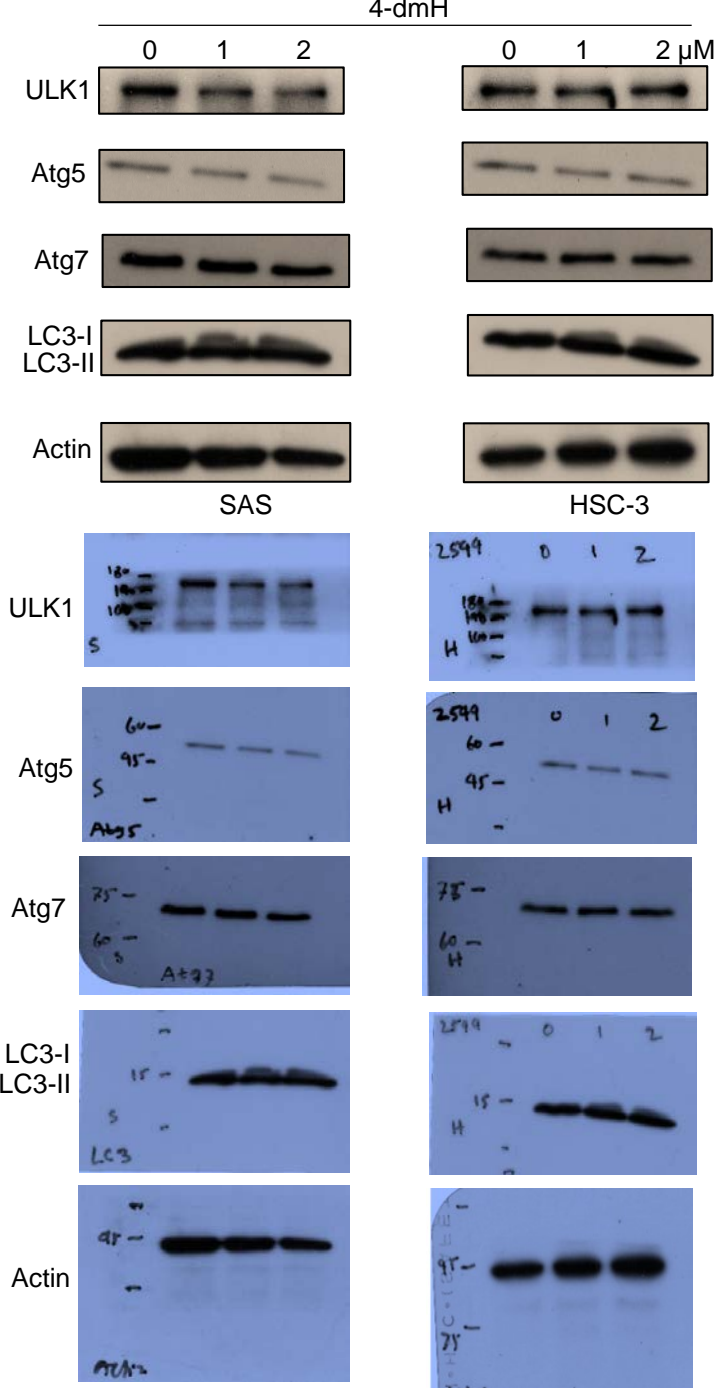

Supplement: Figure 5—source data 4. [file elife-87873-fig5-data4.zip › Figure 5-source data 4.pdf.pdf]

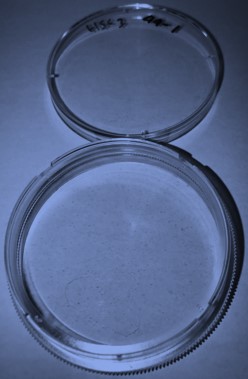

Supplement: Figure 6—source data 1. [file elife-87873-fig6-data1.zip › Figure 6-source data 1/Figure 6c ( HSC-3)-4 dmH 1 μM.jpg]

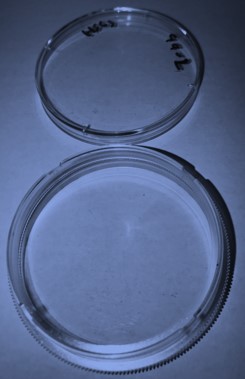

Supplement: Figure 6—source data 1. [file elife-87873-fig6-data1.zip › Figure 6-source data 1/Figure 6c ( HSC-3)-4 dmH 2 μM.jpg]

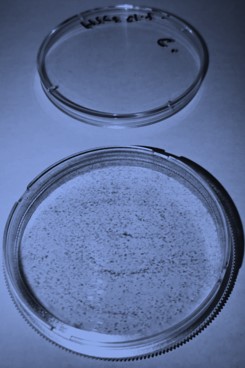

Supplement: Figure 6—source data 1. [file elife-87873-fig6-data1.zip › Figure 6-source data 1/Figure 6c ( HSC-3)-control (DMSO).jpg]

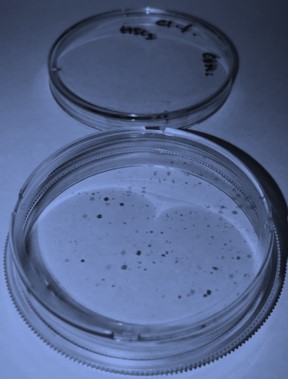

Supplement: Figure 6—source data 1. [file elife-87873-fig6-data1.zip › Figure 6-source data 1/Figure 6c ( HSC-3)-control (H20).jpg]

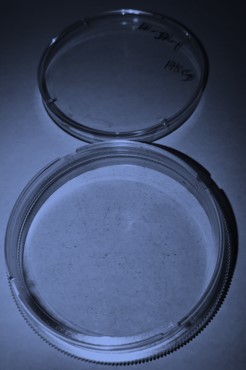

Supplement: Figure 6—source data 1. [file elife-87873-fig6-data1.zip › Figure 6-source data 1/Figure 6c ( HSC-3)-Heliomycin 1 μM.jpg]

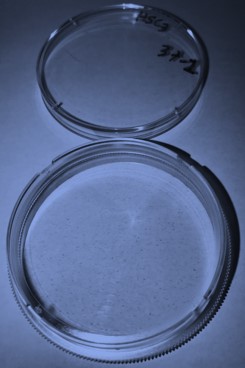

Supplement: Figure 6—source data 1. [file elife-87873-fig6-data1.zip › Figure 6-source data 1/Figure 6c ( HSC-3)-Heliomycin 2 μM.jpg]

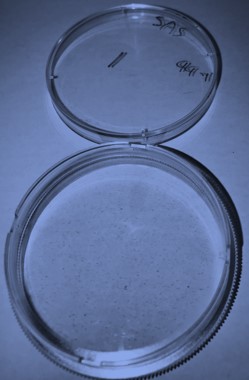

Supplement: Figure 6—source data 1. [file elife-87873-fig6-data1.zip › Figure 6-source data 1/Figure 6c ( SAS)-4 dmH 1 μM.jpg]

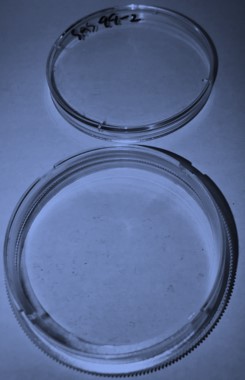

Supplement: Figure 6—source data 1. [file elife-87873-fig6-data1.zip › Figure 6-source data 1/Figure 6c ( SAS)-4 dmH 2 μM.jpg]

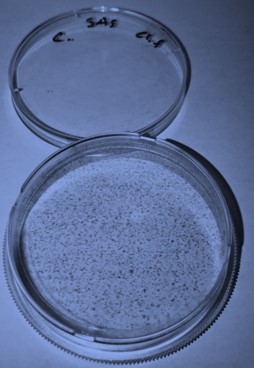

Supplement: Figure 6—source data 1. [file elife-87873-fig6-data1.zip › Figure 6-source data 1/Figure 6c ( SAS)-control (DMSO).jpg]

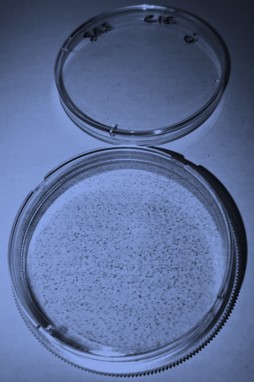

Supplement: Figure 6—source data 1. [file elife-87873-fig6-data1.zip › Figure 6-source data 1/Figure 6c ( SAS)-control (H20).jpg]

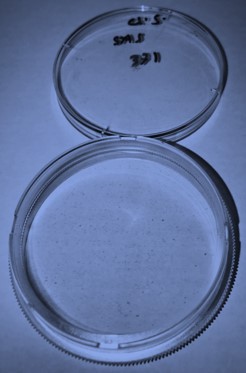

Supplement: Figure 6—source data 1. [file elife-87873-fig6-data1.zip › Figure 6-source data 1/Figure 6c ( SAS)-Heliomycin 1 μM.jpg]

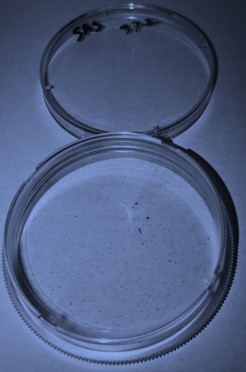

Supplement: Figure 6—source data 1. [file elife-87873-fig6-data1.zip › Figure 6-source data 1/Figure 6c ( SAS)-Heliomycin 2 μM.jpg]

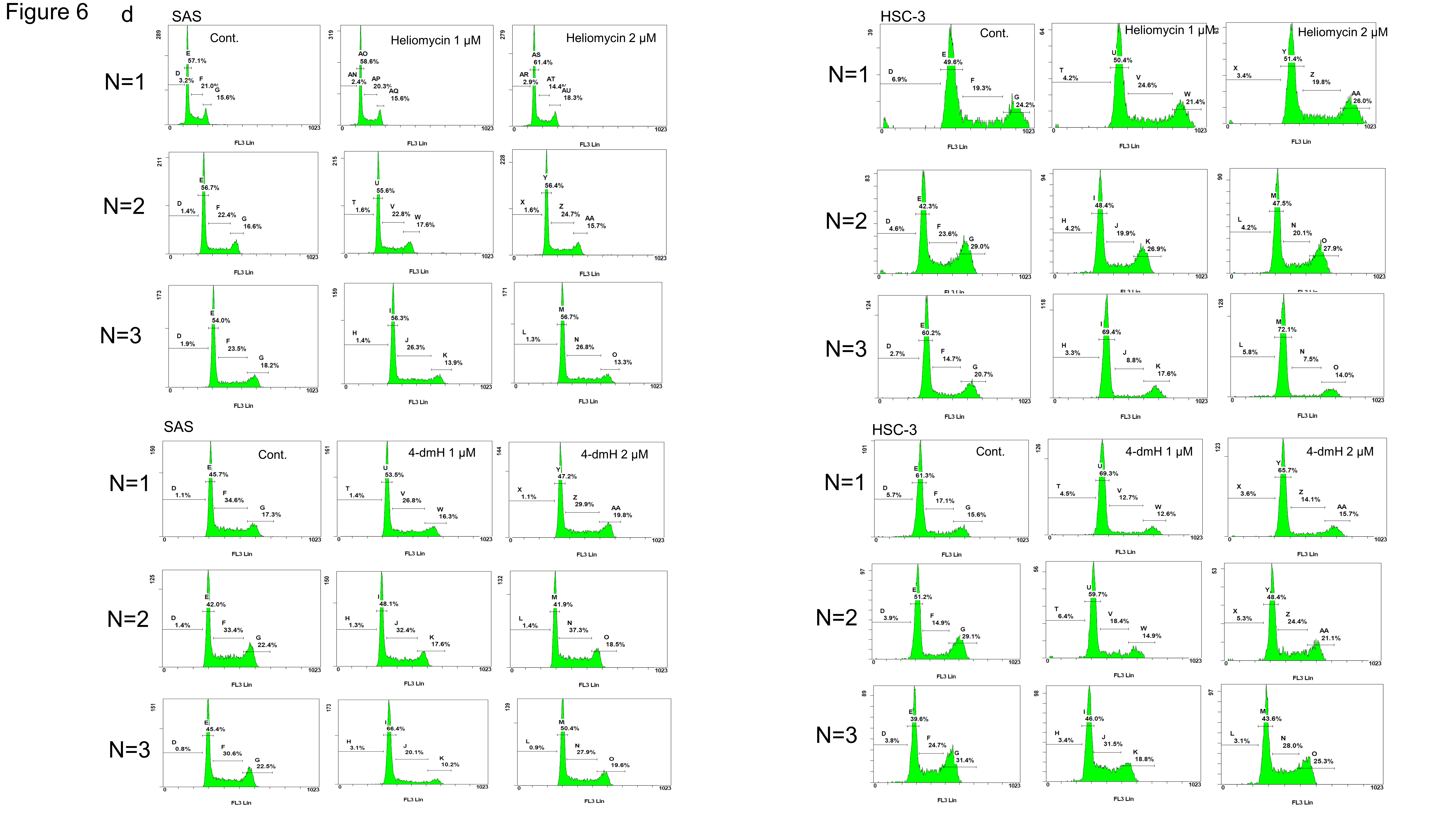

Supplement: Figure 6—source data 2. [file elife-87873-fig6-data2.zip › Figure 6-source data 2.tif]

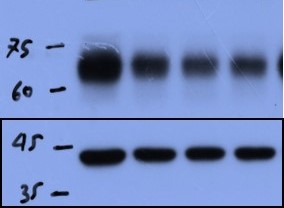

Supplement: Figure 7—source data 1. [file elife-87873-fig7-data1.zip › Figure 7-source data 1/Figure 7b (HSC-3)-Actin-4 dmH (N=1).jpg]

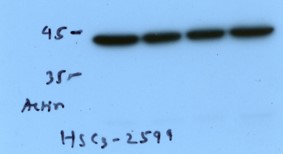

Supplement: Figure 7—source data 1. [file elife-87873-fig7-data1.zip › Figure 7-source data 1/Figure 7b (HSC-3)-Actin-4 dmH (N=2).jpg]

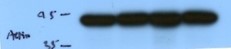

Supplement: Figure 7—source data 1. [file elife-87873-fig7-data1.zip › Figure 7-source data 1/Figure 7b (HSC-3)-Actin-4 dmH (N=3).jpg]

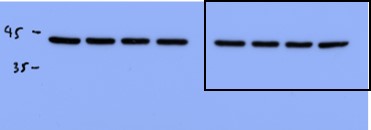

Supplement: Figure 7—source data 1. [file elife-87873-fig7-data1.zip › Figure 7-source data 1/Figure 7b (HSC-3)-Actin-Heliomycin.jpg]

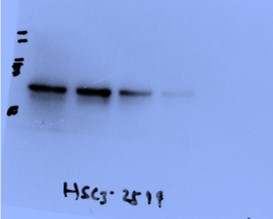

Supplement: Figure 7—source data 1. [file elife-87873-fig7-data1.zip › Figure 7-source data 1/Figure 7b (HSC-3)-tNOX-4 dmH (N=1).jpg]

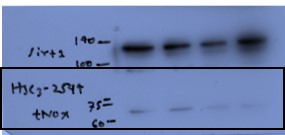

Supplement: Figure 7—source data 1. [file elife-87873-fig7-data1.zip › Figure 7-source data 1/Figure 7b (HSC-3)-tNOX-4 dmH (N=2).jpg]

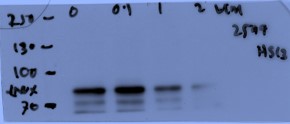

Supplement: Figure 7—source data 1. [file elife-87873-fig7-data1.zip › Figure 7-source data 1/Figure 7b (HSC-3)-tNOX-4 dmH (N=3).jpg]

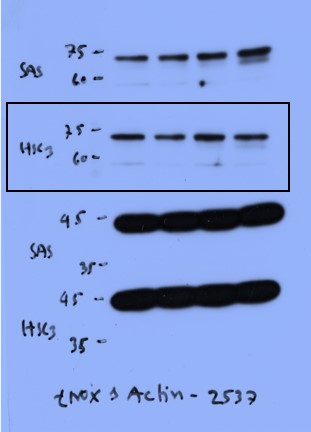

Supplement: Figure 7—source data 1. [file elife-87873-fig7-data1.zip › Figure 7-source data 1/Figure 7b (HSC-3)-tNOX-Heliomycin.jpg]

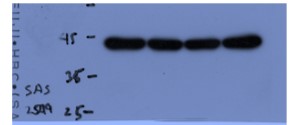

Supplement: Figure 7—source data 1. [file elife-87873-fig7-data1.zip › Figure 7-source data 1/Figure 7b (SAS)-Actin-4 dmH (N=1).jpg]

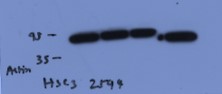

Supplement: Figure 7—source data 1. [file elife-87873-fig7-data1.zip › Figure 7-source data 1/Figure 7b (SAS)-Actin-4 dmH (N=2).jpg]

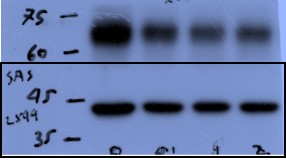

Supplement: Figure 7—source data 1. [file elife-87873-fig7-data1.zip › Figure 7-source data 1/Figure 7b (SAS)-Actin-4 dmH (N=3).jpg]

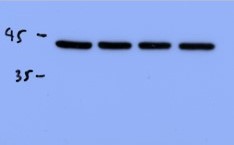

Supplement: Figure 7—source data 1. [file elife-87873-fig7-data1.zip › Figure 7-source data 1/Figure 7b (SAS)-Actin-Heliomycin.jpg]

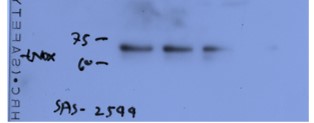

Supplement: Figure 7—source data 1. [file elife-87873-fig7-data1.zip › Figure 7-source data 1/Figure 7b (SAS)-tNOX-4 dmH (N=1).jpg]

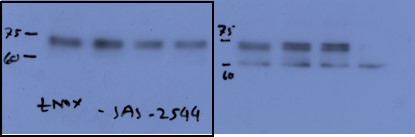

Supplement: Figure 7—source data 1. [file elife-87873-fig7-data1.zip › Figure 7-source data 1/Figure 7b (SAS)-tNOX-4 dmH (N=2).jpg]

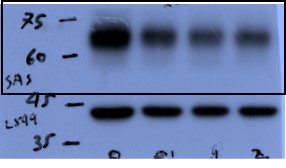

Supplement: Figure 7—source data 1. [file elife-87873-fig7-data1.zip › Figure 7-source data 1/Figure 7b (SAS)-tNOX-4 dmH (N=3).jpg]

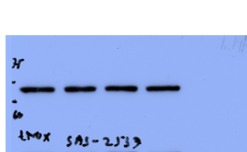

Supplement: Figure 7—source data 1. [file elife-87873-fig7-data1.zip › Figure 7-source data 1/Figure 7b (SAS)-tNOX-Heliomycin.png]

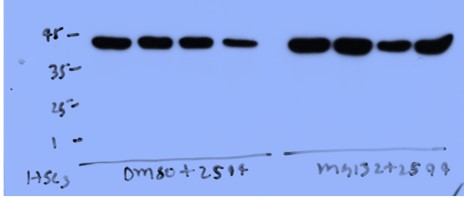

Supplement: Figure 7—source data 1. [file elife-87873-fig7-data1.zip › Figure 7-source data 1/Figure 7c (HSC-3)-Actin-4 dmH (N=1).jpg]

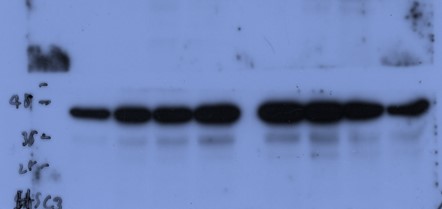

Supplement: Figure 7—source data 1. [file elife-87873-fig7-data1.zip › Figure 7-source data 1/Figure 7c (HSC-3)-Actin-4 dmH (N=2).jpg]

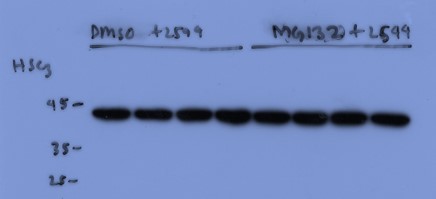

Supplement: Figure 7—source data 1. [file elife-87873-fig7-data1.zip › Figure 7-source data 1/Figure 7c (HSC-3)-Actin-4 dmH (N=3).jpg]

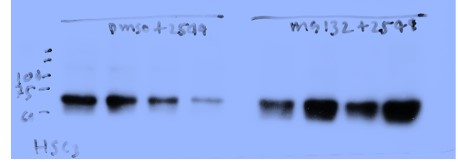

Supplement: Figure 7—source data 1. [file elife-87873-fig7-data1.zip › Figure 7-source data 1/Figure 7c (HSC-3)-tNOX-4 dmH (N=1).jpg]

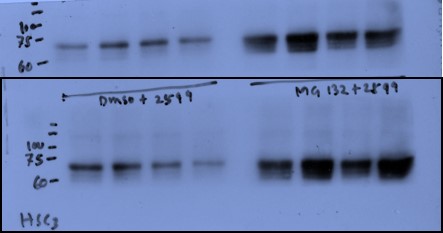

Supplement: Figure 7—source data 1. [file elife-87873-fig7-data1.zip › Figure 7-source data 1/Figure 7c (HSC-3)-tNOX-4 dmH (N=2).jpg]

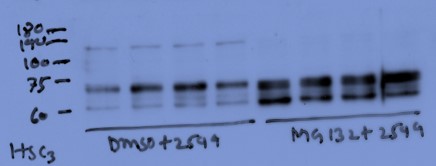

Supplement: Figure 7—source data 1. [file elife-87873-fig7-data1.zip › Figure 7-source data 1/Figure 7c (HSC-3)-tNOX-4 dmH (N=3).jpg]

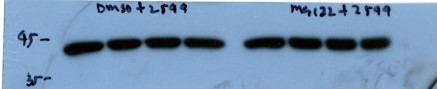

Supplement: Figure 7—source data 1. [file elife-87873-fig7-data1.zip › Figure 7-source data 1/Figure 7c (SAS)-Actin-4 dmH (N=1).jpg]

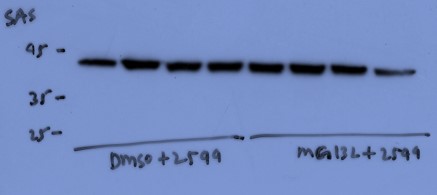

Supplement: Figure 7—source data 1. [file elife-87873-fig7-data1.zip › Figure 7-source data 1/Figure 7c (SAS)-Actin-4 dmH (N=2).jpg]
