# Supplementary material for: Water-soluble 4-(dimethylaminomethyl)heliomycin exerts greater antitumor effects than parental heliomycin by targeting the tNOX-SIRT1 axis and apoptosis in oral cancer cells
Source: eLife. 2024 Apr 3;12:RP87873. doi: 10.7554/eLife.87873 (PMC10990494; doi:10.7554/eLife.87873)
Supplement: Figure 4—source data 2. [file elife-87873-fig4-data2.zip › Figure 4-source data 2.PDF]

Figure 4

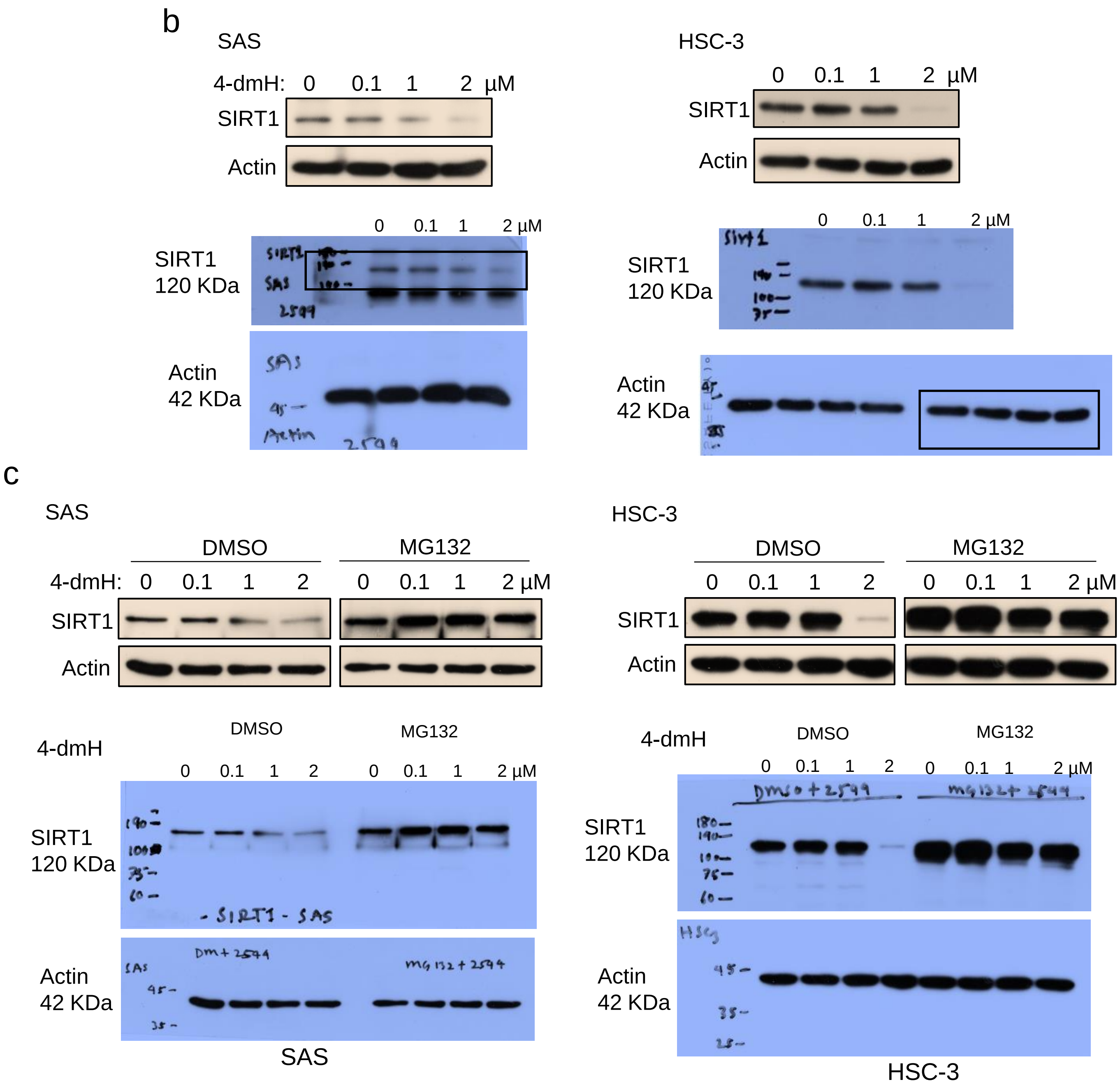
b
SAS
4-dmH: 0 0.1 1 2 µM
SIRT1
Actin
HSC-3
0 0.1 1 2 µM
SIRT1
Actin
0 0.1 1 2 µM
SIRT1
120 KDa
Actin
42 KDa
0 0.1 1 2 µM
SIRT1
120 KDa
Actin
42 KDa
c
SAS
DMSO
MG132
4-dmH: 0 0.1 1 2 0 0.1 1 2 µM
SIRT1
Actin
HSC-3
DMSO
MG132
0 0.1 1 2 0 0.1 1 2 µM
SIRT1
Actin
4-dmH
DMSO
MG132
0 0.1 1 2 0 0.1 1 2 µM
SIRT1
120 KDa
Actin
42 KDa
SAS
4-dmH
DMSO
MG132
0 0.1 1 2 0 0.1 1 2 µM
SIRT1
120 KDa
Actin
42 KDa
HSC-3

Figure 4

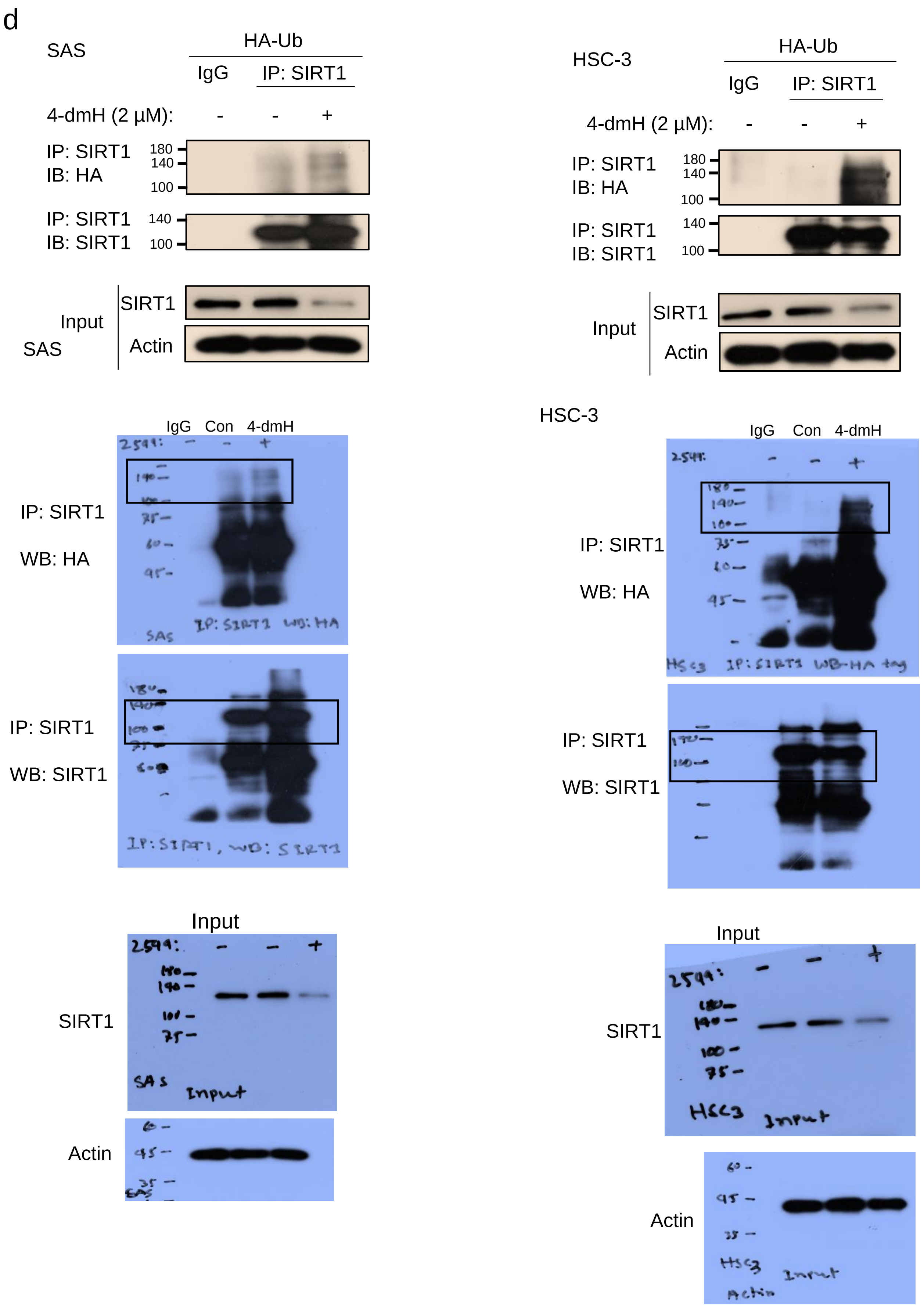

d
SAS
HA-Ub
IgG
IP: SIRT1
4-dmH (2 μM):
-
-
+
IP: SIRT1
IB: HA
180
140
100
IP: SIRT1
IB: SIRT1
140
100
Input
SAS
SIRT1
Actin
HSC-3
HA-Ub
IgG
IP: SIRT1
4-dmH (2 μM):
-
-
+
IP: SIRT1
IB: HA
180
140
100
IP: SIRT1
IB: SIRT1
140
100
Input
SIRT1
Actin
IgG
Con
4-dmH
IP: SIRT1
WB: HA
IP: SIRT1
WB: SIRT1
HSC-3
IgG
Con
4-dmH
IP: SIRT1
WB: HA
IP: SIRT1
WB: SIRT1
Input
SIRT1
Actin
Input
SIRT1
Actin
