# Supplementary figures and images for: Water-soluble 4-(dimethylaminomethyl)heliomycin exerts greater antitumor effects than parental heliomycin by targeting the tNOX-SIRT1 axis and apoptosis in oral cancer cells (part 3 of 3)
Source: eLife. 2024 Apr 3;12:RP87873. doi: 10.7554/eLife.87873 (PMC10990494; doi:10.7554/eLife.87873)

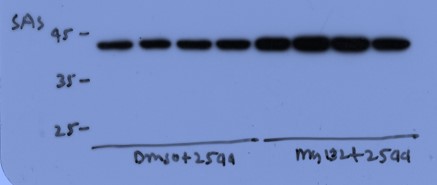

Supplement: Figure 7—source data 1. [file elife-87873-fig7-data1.zip › Figure 7-source data 1/Figure 7c (SAS)-Actin-4 dmH (N=3).jpg]

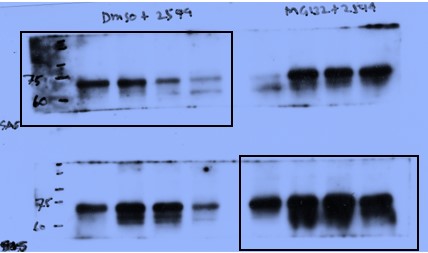

Supplement: Figure 7—source data 1. [file elife-87873-fig7-data1.zip › Figure 7-source data 1/Figure 7c (SAS)-tNOX-4 dmH (N=1).jpg]

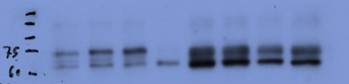

Supplement: Figure 7—source data 1. [file elife-87873-fig7-data1.zip › Figure 7-source data 1/Figure 7c (SAS)-tNOX-4 dmH (N=2).jpg]

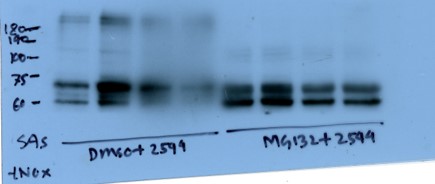

Supplement: Figure 7—source data 1. [file elife-87873-fig7-data1.zip › Figure 7-source data 1/Figure 7c (SAS)-tNOX-4 dmH (N=3).jpg]

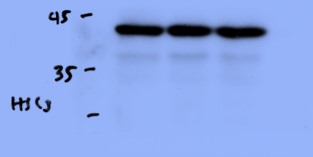

Supplement: Figure 7—source data 1. [file elife-87873-fig7-data1.zip › Figure 7-source data 1/Figure 7d (HSC-3)-Actin-input.jpg]

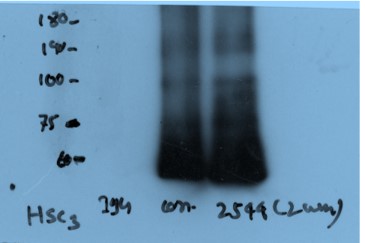

Supplement: Figure 7—source data 1. [file elife-87873-fig7-data1.zip › Figure 7-source data 1/Figure 7d (HSC-3)-IP-ENOX2, IB-HA.jpg]

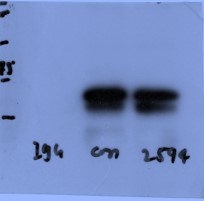

Supplement: Figure 7—source data 1. [file elife-87873-fig7-data1.zip › Figure 7-source data 1/Figure 7d (HSC-3)-IP-ENOX2, IB-tNOX.jpg]

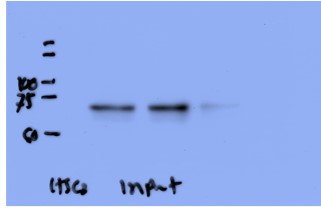

Supplement: Figure 7—source data 1. [file elife-87873-fig7-data1.zip › Figure 7-source data 1/Figure 7d (HSC-3)-tNOX-input.jpg]

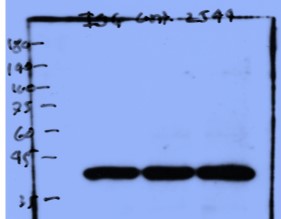

Supplement: Figure 7—source data 1. [file elife-87873-fig7-data1.zip › Figure 7-source data 1/Figure 7d (SAS)-Actin-input.jpg]

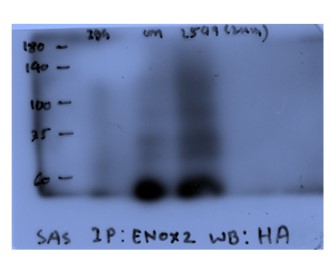

Supplement: Figure 7—source data 1. [file elife-87873-fig7-data1.zip › Figure 7-source data 1/Figure 7d (SAS)-IP-ENOX2, IB-HA.jpg]

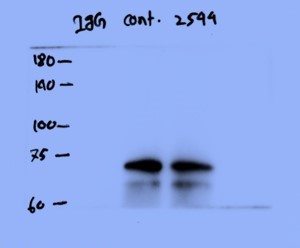

Supplement: Figure 7—source data 1. [file elife-87873-fig7-data1.zip › Figure 7-source data 1/Figure 7d (SAS)-IP-ENOX2, IB-tNOX.jpg]

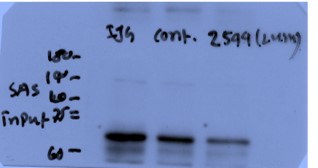

Supplement: Figure 7—source data 1. [file elife-87873-fig7-data1.zip › Figure 7-source data 1/Figure 7d (SAS)-tNOX-input.jpg]

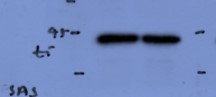

Supplement: Figure 7—source data 1. [file elife-87873-fig7-data1.zip › Figure 7-source data 1/Figure 7e-knockdown-Actin (N=1).jpg]

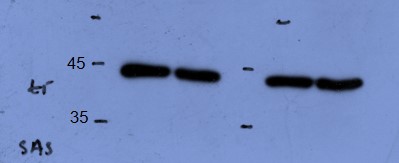

Supplement: Figure 7—source data 1. [file elife-87873-fig7-data1.zip › Figure 7-source data 1/Figure 7e-knockdown-Actin (N=2).jpg]

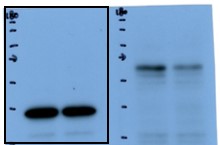

Supplement: Figure 7—source data 1. [file elife-87873-fig7-data1.zip › Figure 7-source data 1/Figure 7e-knockdown-Actin (N=3).jpg]

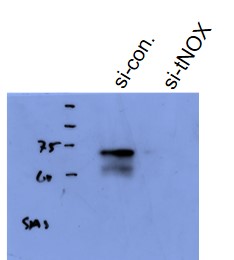

Supplement: Figure 7—source data 1. [file elife-87873-fig7-data1.zip › Figure 7-source data 1/Figure 7e-knockdown-tNOX (N=1).jpg]

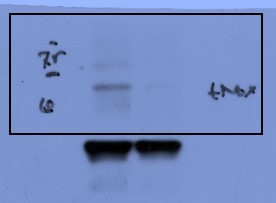

Supplement: Figure 7—source data 1. [file elife-87873-fig7-data1.zip › Figure 7-source data 1/Figure 7e-knockdown-tNOX (N=2).jpg]

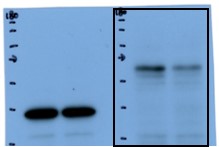

Supplement: Figure 7—source data 1. [file elife-87873-fig7-data1.zip › Figure 7-source data 1/Figure 7e-knockdown-tNOX (N=3).jpg]

Figure 7

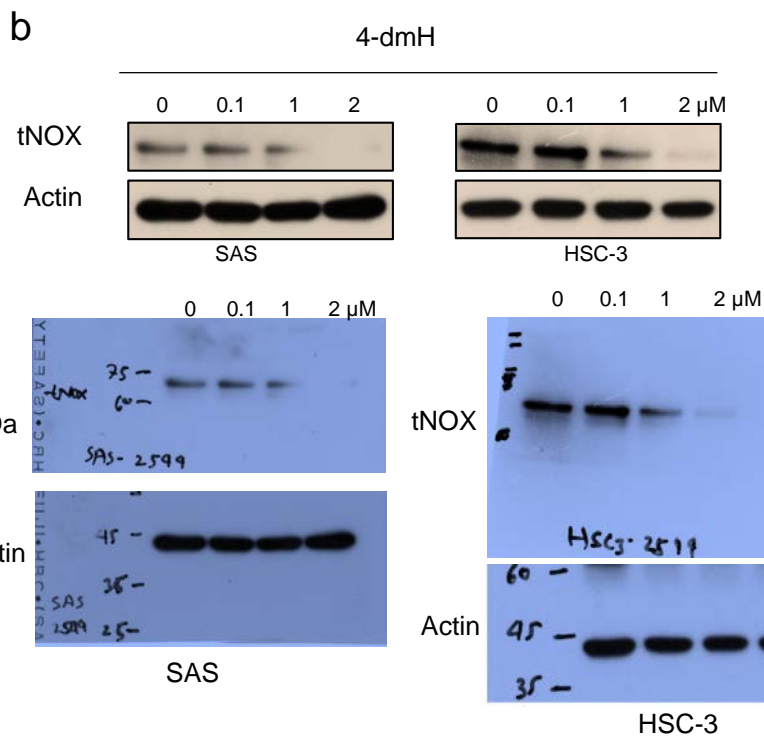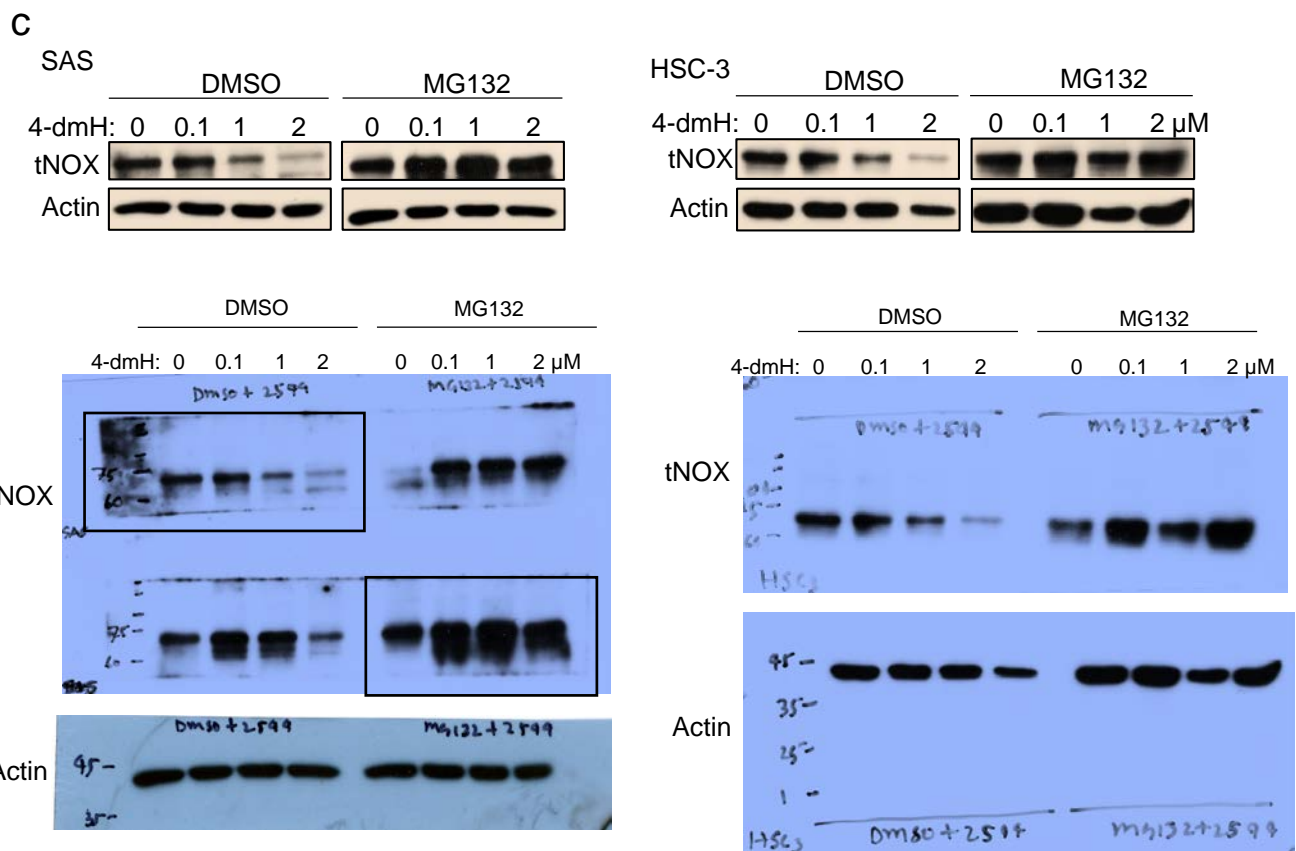

Figure 7

d

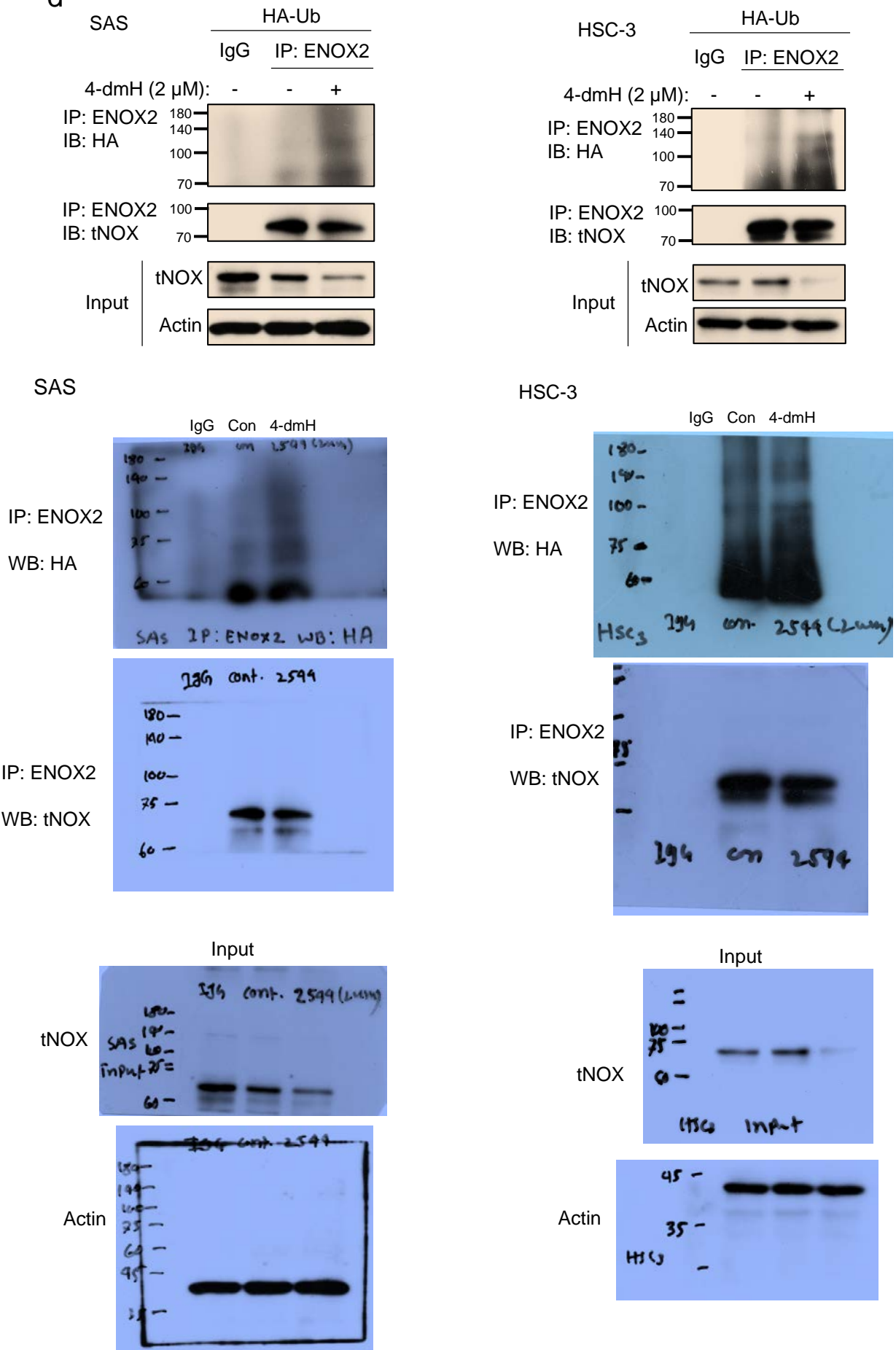

e

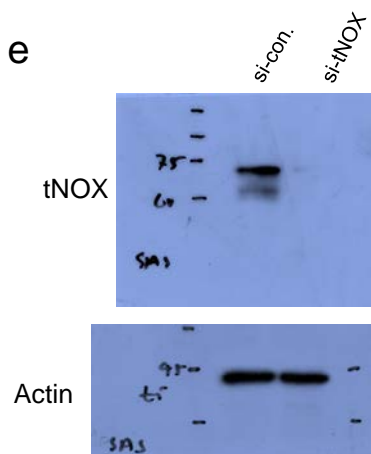

Supplement: Figure 7—source data 2. [file elife-87873-fig7-data2.zip › Figure 7-source data 2.pdf]

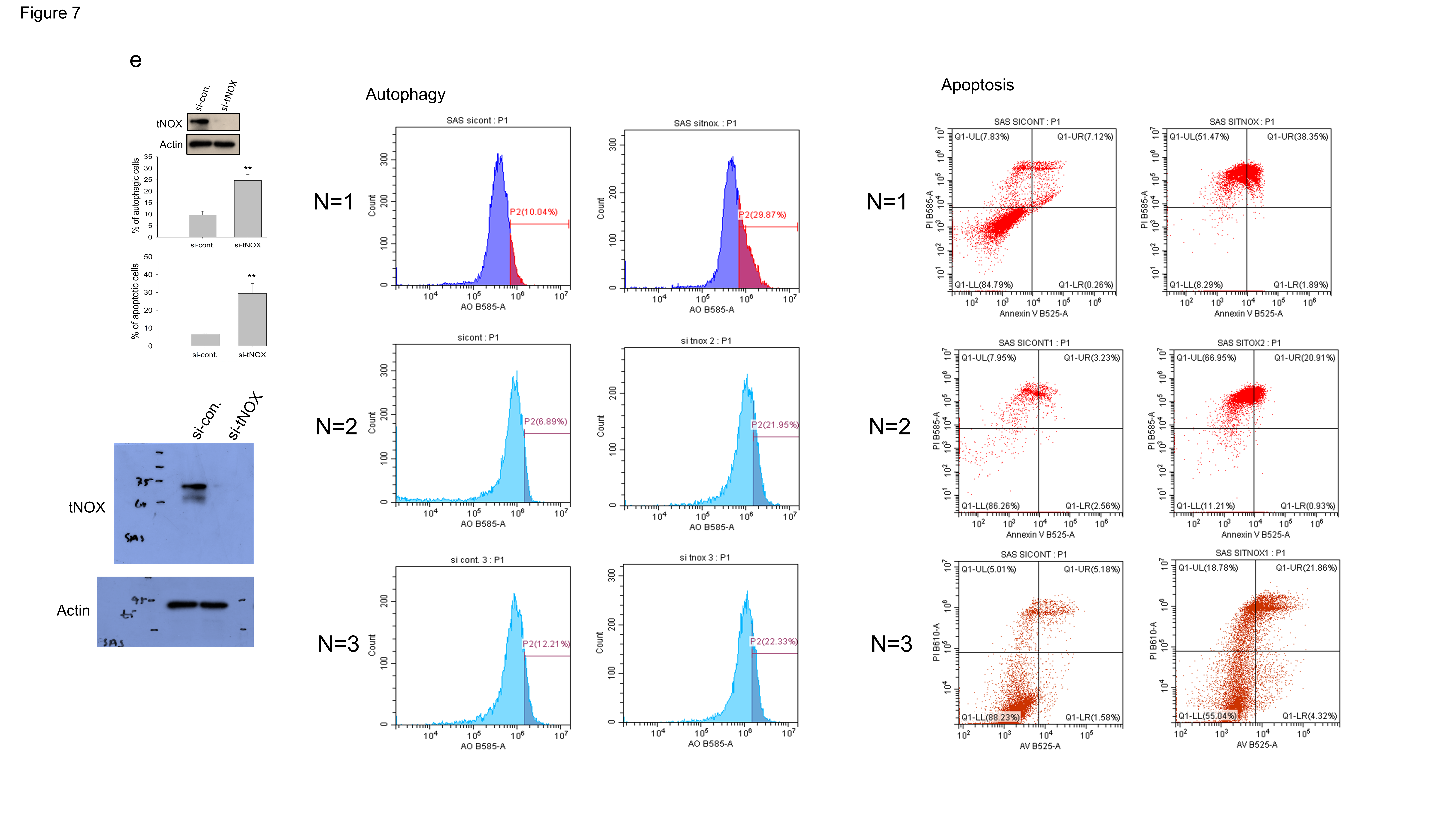

Supplement: Figure 7—source data 3. [file elife-87873-fig7-data3.zip › Figure 7-source data 3.tif]

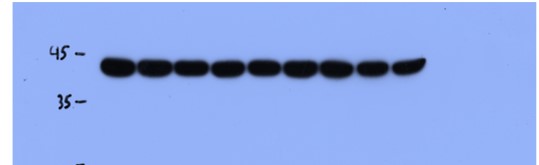

Supplement: Figure 8—source data 1. [file elife-87873-fig8-data1.zip › Figure 8-source data 1/Figure 8a (HSC-3)-Actin-4 dmH (N=1).jpg]

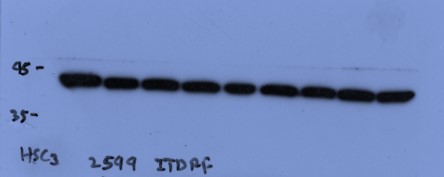

Supplement: Figure 8—source data 1. [file elife-87873-fig8-data1.zip › Figure 8-source data 1/Figure 8a (HSC-3)-Actin-4 dmH (N=2).jpg]

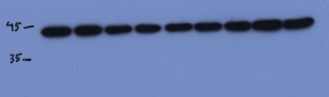

Supplement: Figure 8—source data 1. [file elife-87873-fig8-data1.zip › Figure 8-source data 1/Figure 8a (HSC-3)-Actin-4 dmH (N=3).jpg]

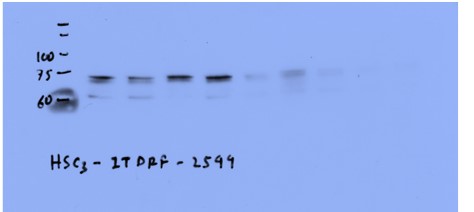

Supplement: Figure 8—source data 1. [file elife-87873-fig8-data1.zip › Figure 8-source data 1/Figure 8a (HSC-3)-tNOX-4 dmH (N=1).jpg]

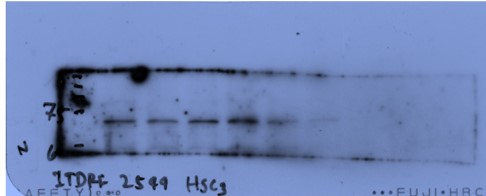

Supplement: Figure 8—source data 1. [file elife-87873-fig8-data1.zip › Figure 8-source data 1/Figure 8a (HSC-3)-tNOX-4 dmH (N=2).jpg]

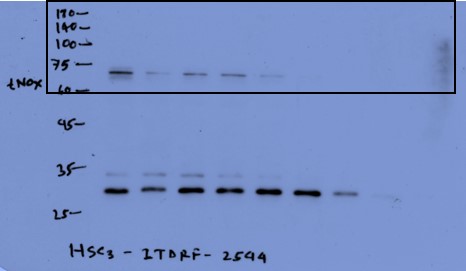

Supplement: Figure 8—source data 1. [file elife-87873-fig8-data1.zip › Figure 8-source data 1/Figure 8a (HSC-3)-tNOX-4 dmH (N=3).jpg]

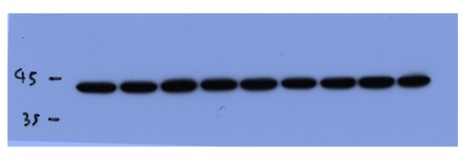

Supplement: Figure 8—source data 1. [file elife-87873-fig8-data1.zip › Figure 8-source data 1/Figure 8a (SAS)-Actin-4 dmH (N=1).jpg]

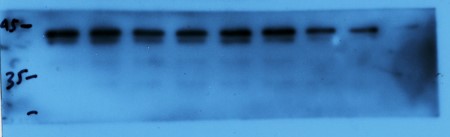

Supplement: Figure 8—source data 1. [file elife-87873-fig8-data1.zip › Figure 8-source data 1/Figure 8a (SAS)-Actin-4 dmH (N=2).jpg]

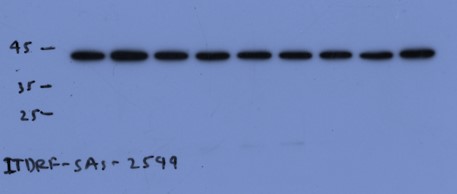

Supplement: Figure 8—source data 1. [file elife-87873-fig8-data1.zip › Figure 8-source data 1/Figure 8a (SAS)-Actin-4 dmH (N=3).jpg]

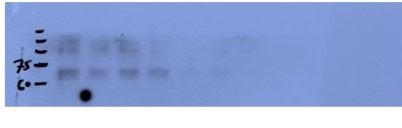

Supplement: Figure 8—source data 1. [file elife-87873-fig8-data1.zip › Figure 8-source data 1/Figure 8a (SAS)-tNOX-4 dmH (N=1).jpg]

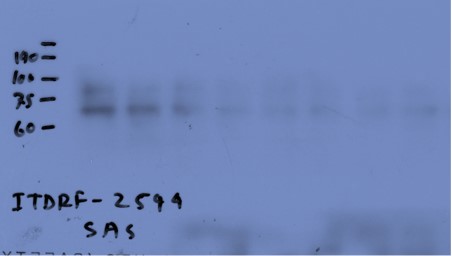

Supplement: Figure 8—source data 1. [file elife-87873-fig8-data1.zip › Figure 8-source data 1/Figure 8a (SAS)-tNOX-4 dmH (N=2).jpg]

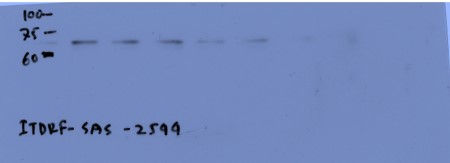

Supplement: Figure 8—source data 1. [file elife-87873-fig8-data1.zip › Figure 8-source data 1/Figure 8a (SAS)-tNOX-4 dmH (N=3).jpg]

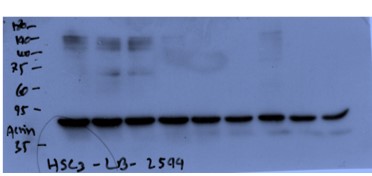

Supplement: Figure 8—source data 1. [file elife-87873-fig8-data1.zip › Figure 8-source data 1/Figure 8b (HSC-3)-Actin-4 dmH (N=1).jpg]

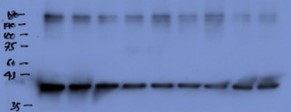

Supplement: Figure 8—source data 1. [file elife-87873-fig8-data1.zip › Figure 8-source data 1/Figure 8b (HSC-3)-Actin-4 dmH (N=2).jpg]

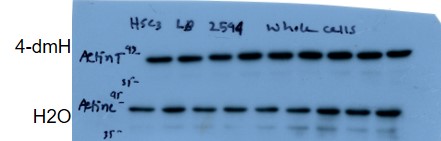

Supplement: Figure 8—source data 1. [file elife-87873-fig8-data1.zip › Figure 8-source data 1/Figure 8b (HSC-3)-Actin-4 dmH and control (N=3).jpg]

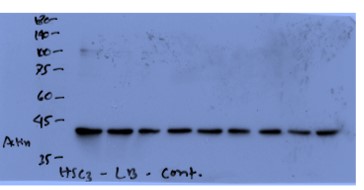

Supplement: Figure 8—source data 1. [file elife-87873-fig8-data1.zip › Figure 8-source data 1/Figure 8b (HSC-3)-Actin-control (H2O) (N=1).jpg]

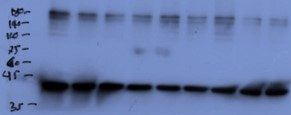

Supplement: Figure 8—source data 1. [file elife-87873-fig8-data1.zip › Figure 8-source data 1/Figure 8b (HSC-3)-Actin-control (H2O) (N=2).jpg]

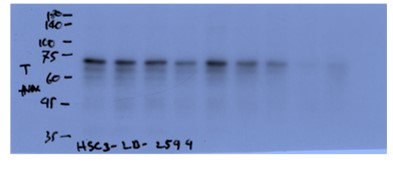

Supplement: Figure 8—source data 1. [file elife-87873-fig8-data1.zip › Figure 8-source data 1/Figure 8b (HSC-3)-tNOX-4 dmH (N=1).jpg]

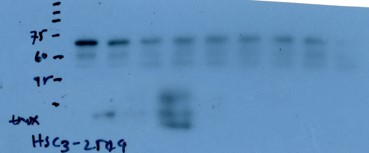

Supplement: Figure 8—source data 1. [file elife-87873-fig8-data1.zip › Figure 8-source data 1/Figure 8b (HSC-3)-tNOX-4 dmH (N=2).jpg]

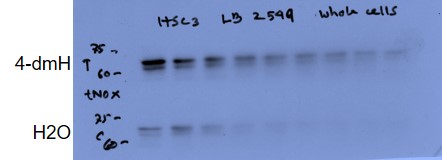

Supplement: Figure 8—source data 1. [file elife-87873-fig8-data1.zip › Figure 8-source data 1/Figure 8b (HSC-3)-tNOX-4 dmH and cont. (N=3).jpg]

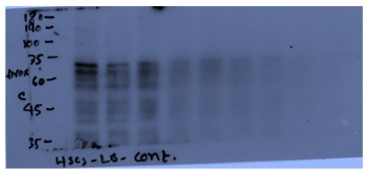

Supplement: Figure 8—source data 1. [file elife-87873-fig8-data1.zip › Figure 8-source data 1/Figure 8b (HSC-3)-tNOX-control (H2O) (N=1).jpg]

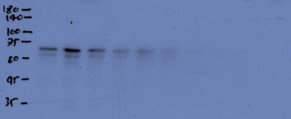

Supplement: Figure 8—source data 1. [file elife-87873-fig8-data1.zip › Figure 8-source data 1/Figure 8b (HSC-3)-tNOX-control (H2O) (N=2).jpg]

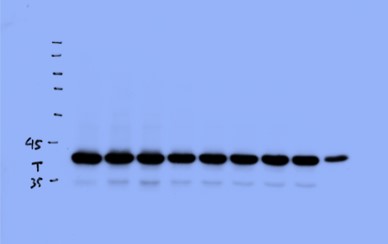

Supplement: Figure 8—source data 1. [file elife-87873-fig8-data1.zip › Figure 8-source data 1/Figure 8b (SAS)-Actin-4 dmH (N=1).jpg]

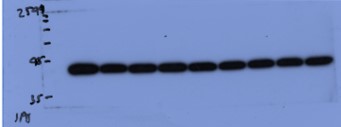

Supplement: Figure 8—source data 1. [file elife-87873-fig8-data1.zip › Figure 8-source data 1/Figure 8b (SAS)-Actin-4 dmH (N=2).jpg]

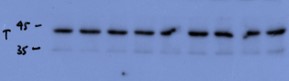

Supplement: Figure 8—source data 1. [file elife-87873-fig8-data1.zip › Figure 8-source data 1/Figure 8b (SAS)-Actin-4 dmH (N=3).jpg]

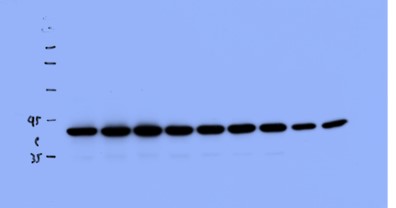

Supplement: Figure 8—source data 1. [file elife-87873-fig8-data1.zip › Figure 8-source data 1/Figure 8b (SAS)-Actin-control (H2O) (N=1).jpg]

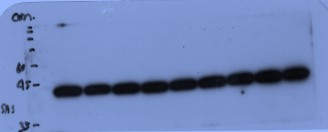

Supplement: Figure 8—source data 1. [file elife-87873-fig8-data1.zip › Figure 8-source data 1/Figure 8b (SAS)-Actin-control (H2O) (N=2).jpg]

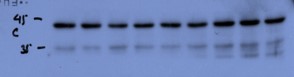

Supplement: Figure 8—source data 1. [file elife-87873-fig8-data1.zip › Figure 8-source data 1/Figure 8b (SAS)-Actin-control (H2O) (N=3).jpg]

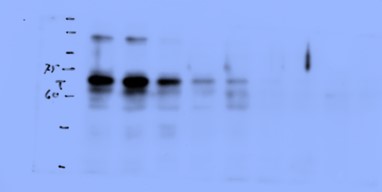

Supplement: Figure 8—source data 1. [file elife-87873-fig8-data1.zip › Figure 8-source data 1/Figure 8b (SAS)-tNOX-4 dmH (N=1).jpg]

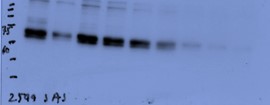

Supplement: Figure 8—source data 1. [file elife-87873-fig8-data1.zip › Figure 8-source data 1/Figure 8b (SAS)-tNOX-4 dmH (N=2).jpg]

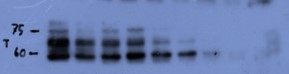

Supplement: Figure 8—source data 1. [file elife-87873-fig8-data1.zip › Figure 8-source data 1/Figure 8b (SAS)-tNOX-4 dmH (N=3).jpg]

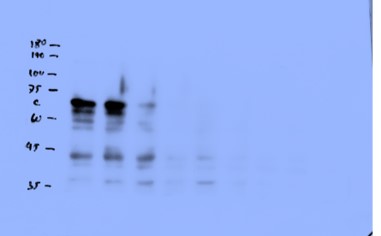

Supplement: Figure 8—source data 1. [file elife-87873-fig8-data1.zip › Figure 8-source data 1/Figure 8b (SAS)-tNOX-control (H2O) (N=1).jpg]

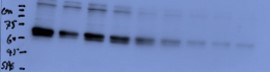

Supplement: Figure 8—source data 1. [file elife-87873-fig8-data1.zip › Figure 8-source data 1/Figure 8b (SAS)-tNOX-control (H2O) (N=2).jpg]

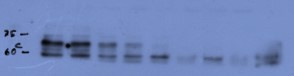

Supplement: Figure 8—source data 1. [file elife-87873-fig8-data1.zip › Figure 8-source data 1/Figure 8b (SAS)-tNOX-control (H2O) (N=3).jpg]

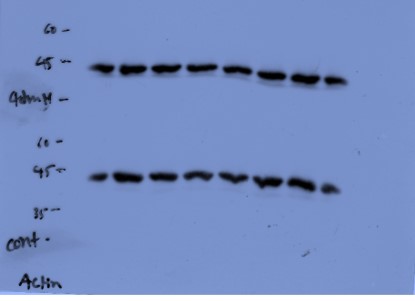

Supplement: Figure 8—source data 1. [file elife-87873-fig8-data1.zip › Figure 8-source data 1/Figure 8c ( SAS)-Actin for Nox4.jpg]

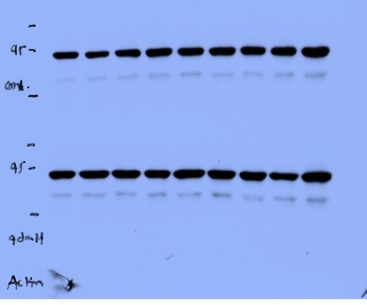

Supplement: Figure 8—source data 1. [file elife-87873-fig8-data1.zip › Figure 8-source data 1/Figure 8c ( SAS)-Actin for PARP.jpg]

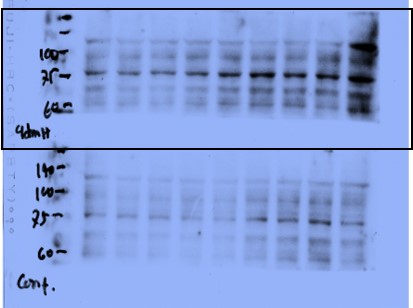

Supplement: Figure 8—source data 1. [file elife-87873-fig8-data1.zip › Figure 8-source data 1/Figure 8c ( SAS)-Nox4-4 dmH.jpg]

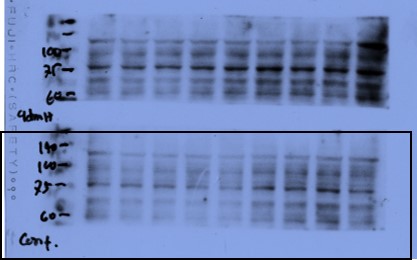

Supplement: Figure 8—source data 1. [file elife-87873-fig8-data1.zip › Figure 8-source data 1/Figure 8c ( SAS)-Nox4-control (H2O).jpg]

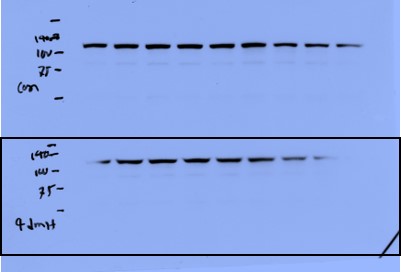

Supplement: Figure 8—source data 1. [file elife-87873-fig8-data1.zip › Figure 8-source data 1/Figure 8c ( SAS)-PARP-4 dmH.jpg]

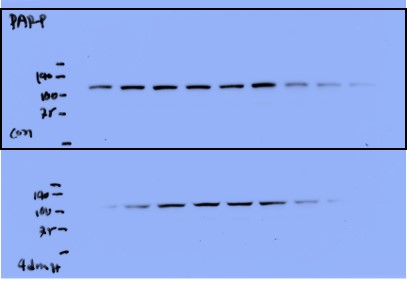

Supplement: Figure 8—source data 1. [file elife-87873-fig8-data1.zip › Figure 8-source data 1/Figure 8c ( SAS)-PARP-Control (H2O).jpg]

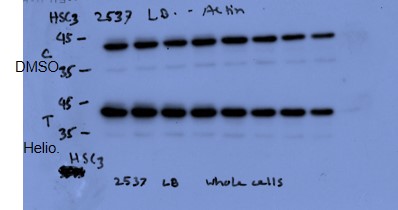

Supplement: Figure 8—source data 1. [file elife-87873-fig8-data1.zip › Figure 8-source data 1/Figure 8d (HSC-3)-Actin -DMSO(N=1).jpg]

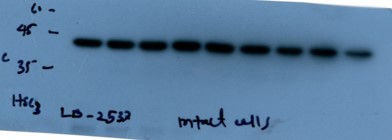

Supplement: Figure 8—source data 1. [file elife-87873-fig8-data1.zip › Figure 8-source data 1/Figure 8d (HSC-3)-Actin-DMSO (N=2).jpg]

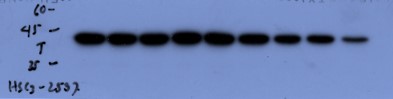

Supplement: Figure 8—source data 1. [file elife-87873-fig8-data1.zip › Figure 8-source data 1/Figure 8d (HSC-3)-Actin-Heliomycin (N=2).jpg]

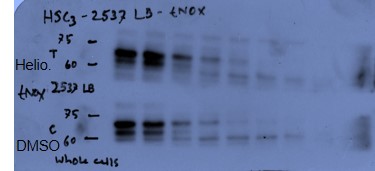

Supplement: Figure 8—source data 1. [file elife-87873-fig8-data1.zip › Figure 8-source data 1/Figure 8d (HSC-3)-tNOX-Heliomycin (N=1).jpg]

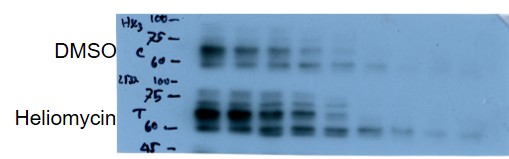

Supplement: Figure 8—source data 1. [file elife-87873-fig8-data1.zip › Figure 8-source data 1/Figure 8d (HSC-3)-tNOX-Heliomycin and DMSO (N=2).jpg]

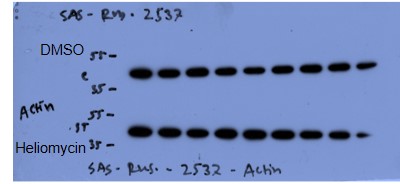

Supplement: Figure 8—source data 1. [file elife-87873-fig8-data1.zip › Figure 8-source data 1/Figure 8d (SAS)-Actin-Heliomycin and DMSO (N=1).jpg]

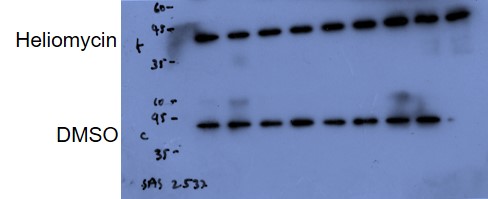

Supplement: Figure 8—source data 1. [file elife-87873-fig8-data1.zip › Figure 8-source data 1/Figure 8d (SAS)-Actin-Heliomycin and DMSO (N=2).jpg]

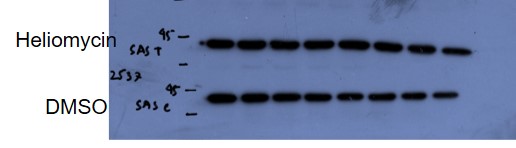

Supplement: Figure 8—source data 1. [file elife-87873-fig8-data1.zip › Figure 8-source data 1/Figure 8d (SAS)-Actin-Heliomycin and DMSO (N=3).jpg]

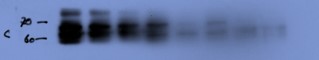

Supplement: Figure 8—source data 1. [file elife-87873-fig8-data1.zip › Figure 8-source data 1/Figure 8d (SAS)-tNOX-DMSO (N=3).jpg]

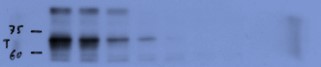

Supplement: Figure 8—source data 1. [file elife-87873-fig8-data1.zip › Figure 8-source data 1/Figure 8d (SAS)-tNOX-Heliomycin (N=3).jpg]

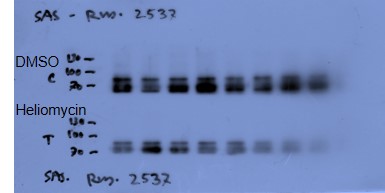

Supplement: Figure 8—source data 1. [file elife-87873-fig8-data1.zip › Figure 8-source data 1/Figure 8d (SAS)-tNOX-Heliomycin and DMSO (N=1).jpg]

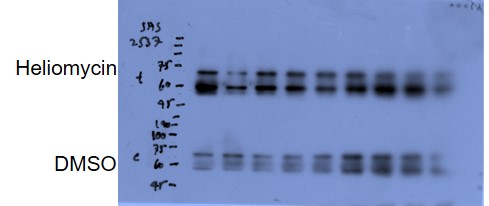

Supplement: Figure 8—source data 1. [file elife-87873-fig8-data1.zip › Figure 8-source data 1/Figure 8d (SAS)-tNOX-Heliomycin and DMSO (N=2).jpg]

Figure 8

a

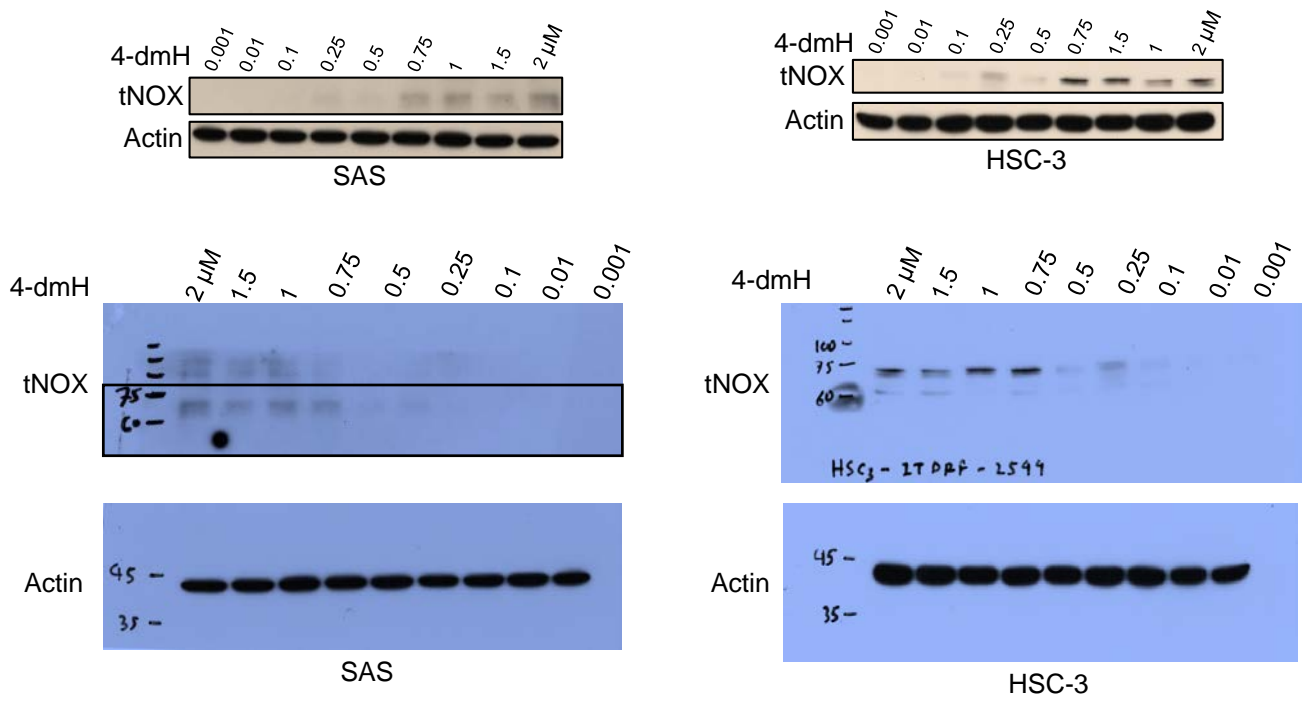

b

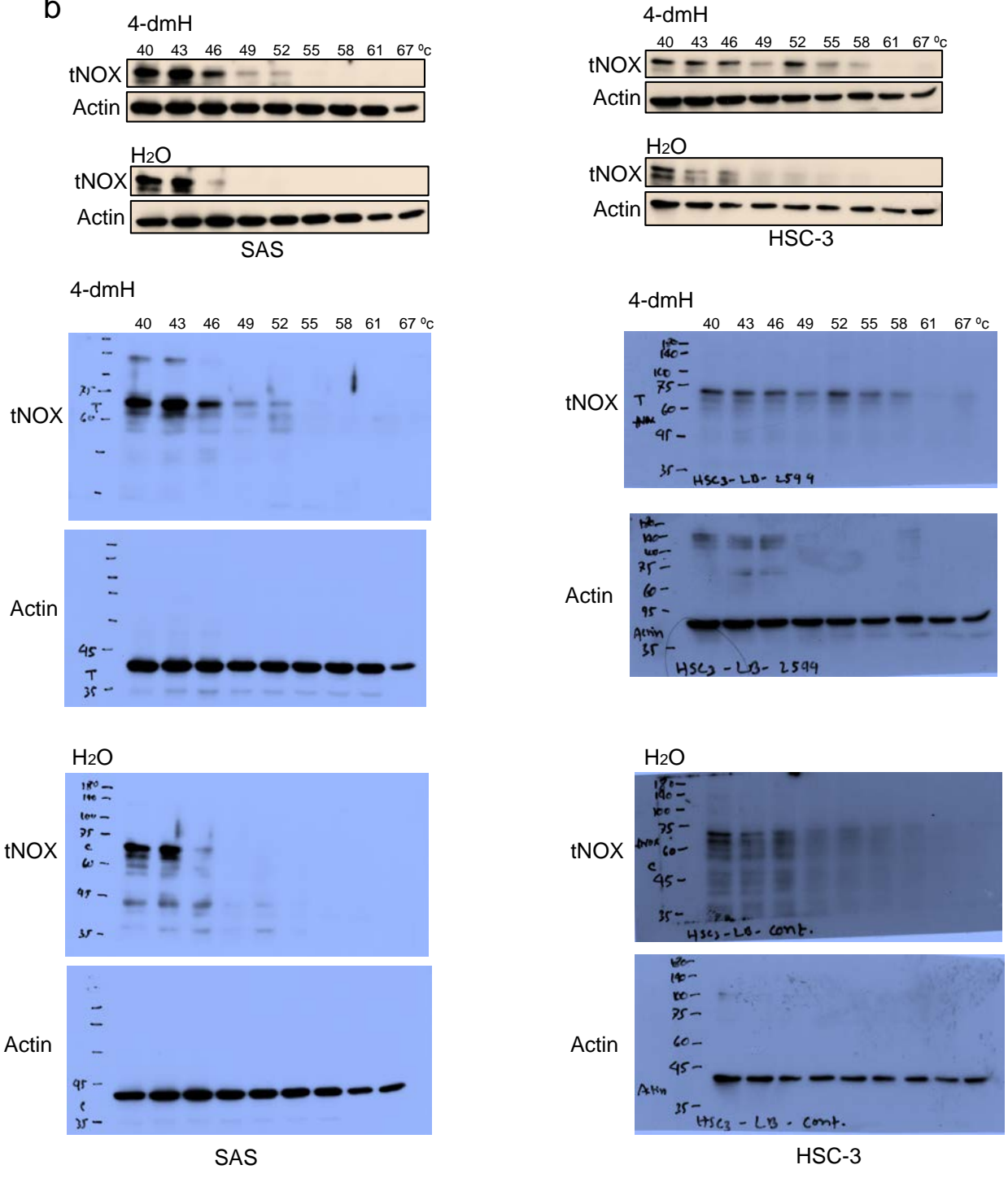

Figure 8

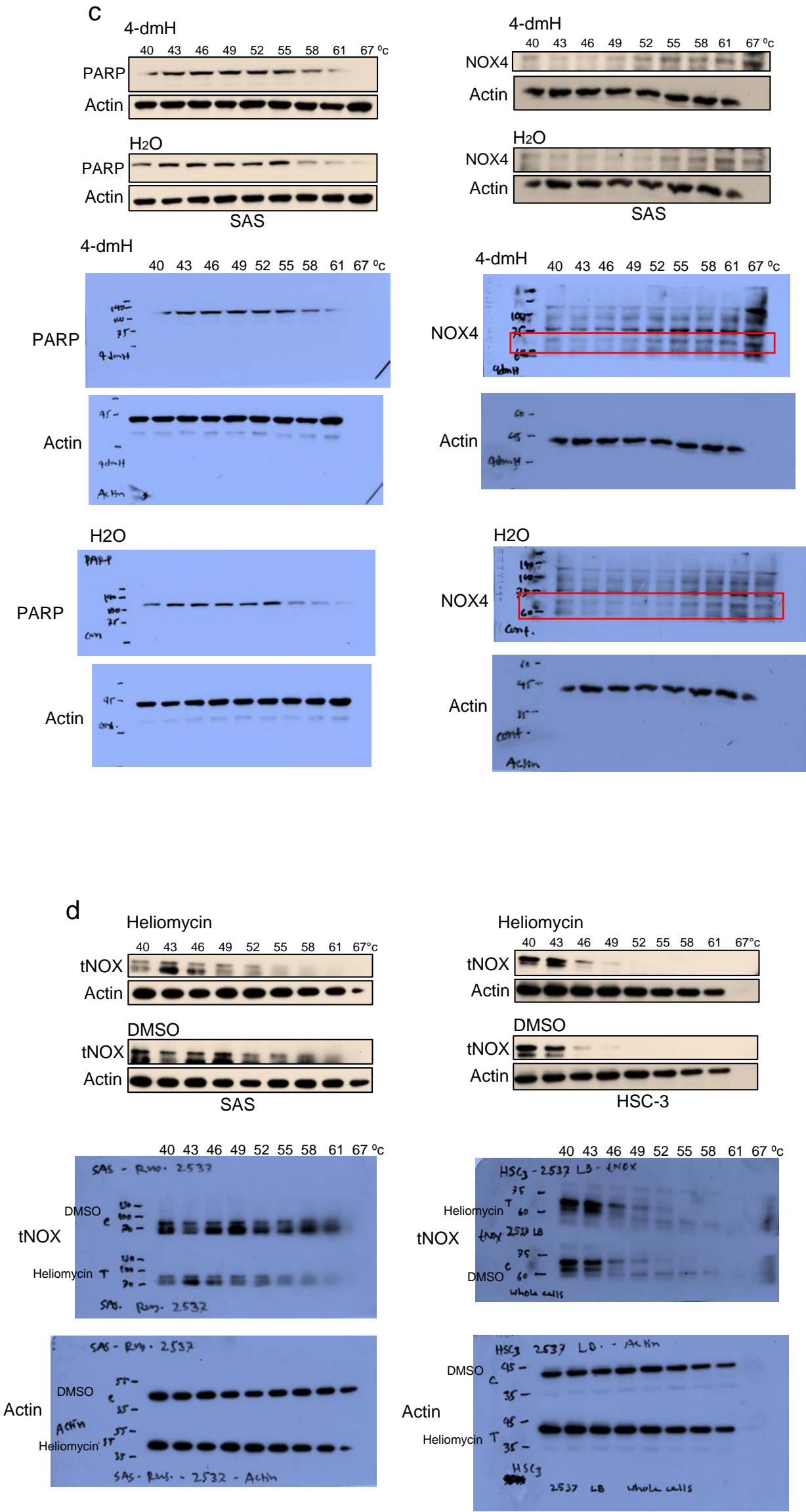

Supplement: Figure 8—source data 2. [file elife-87873-fig8-data2.zip › Figure 8-source data 2.pdf]

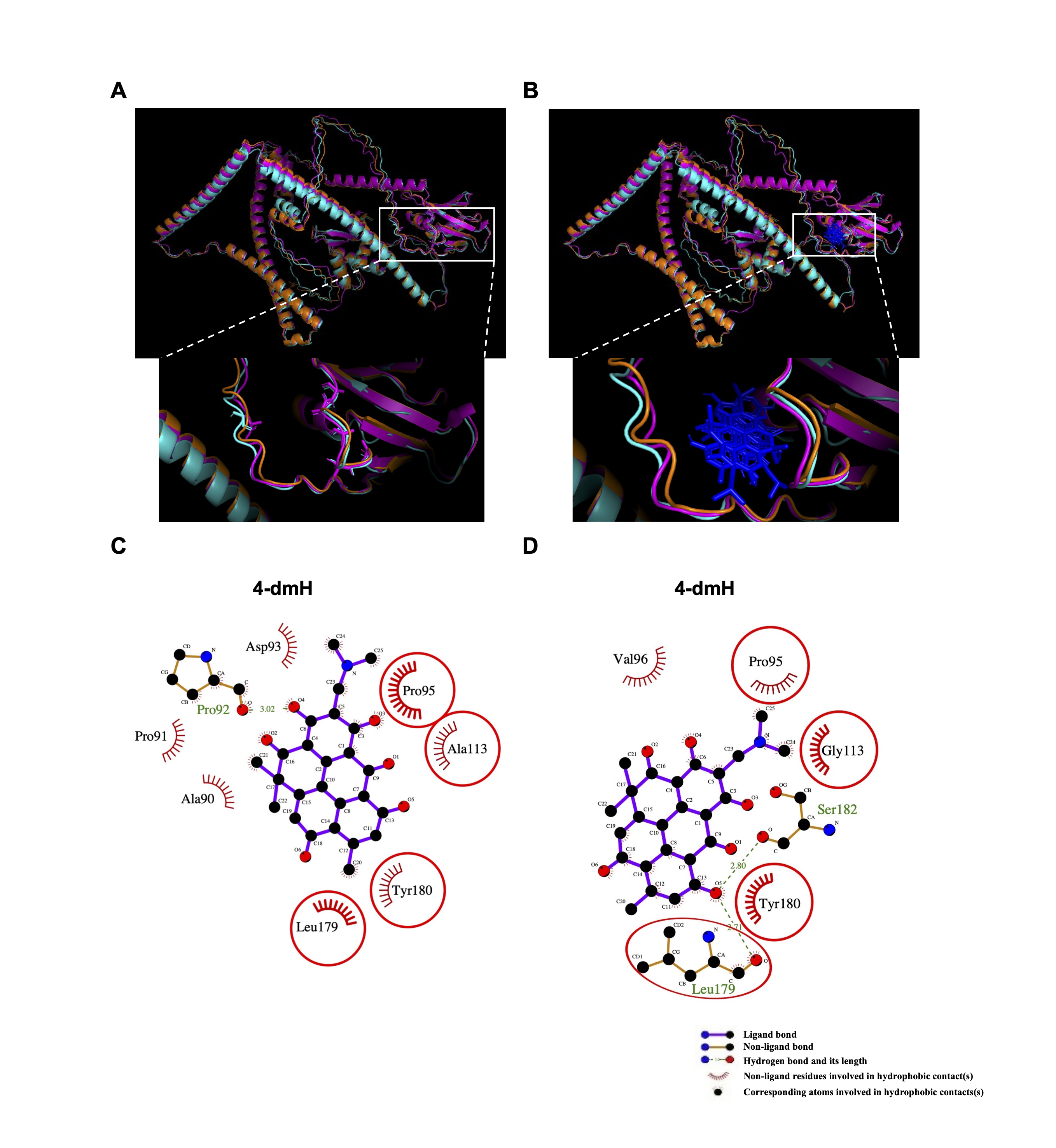

Supplement: Figure 9—figure supplement 1—source data 1. [file elife-87873-fig9-figsupp1-data1.zip › Figure 9-figure supplement 1-source data 1.tif]

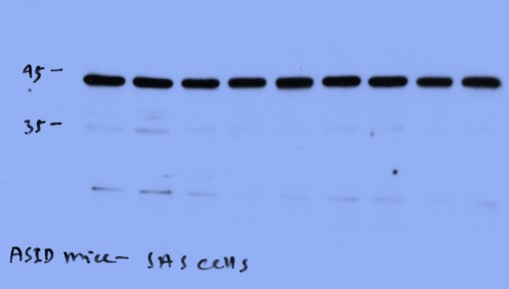

Supplement: Figure 10—source data 1. [file elife-87873-fig10-data1.zip › Figure 10-source data 1/figure 10c-Actin-SAS cells inoculated ASID mice tissue samples.jpg]

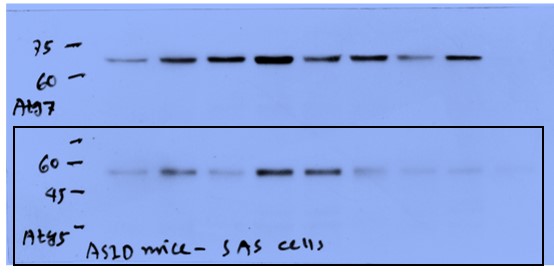

Supplement: Figure 10—source data 1. [file elife-87873-fig10-data1.zip › Figure 10-source data 1/figure 10c-Atg5-SAS cells inoculated ASID mice tissue samples (1).jpg]

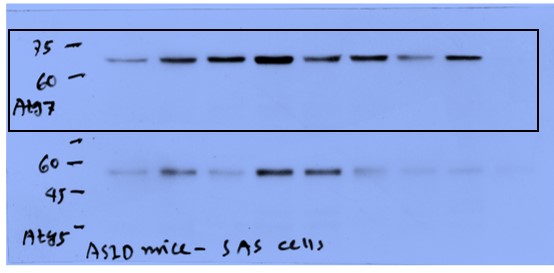

Supplement: Figure 10—source data 1. [file elife-87873-fig10-data1.zip › Figure 10-source data 1/figure 10c-Atg7-SAS cells inoculated ASID mice tissue samples.jpg]

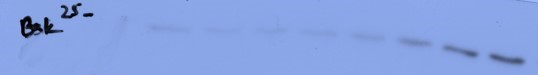

Supplement: Figure 10—source data 1. [file elife-87873-fig10-data1.zip › Figure 10-source data 1/figure 10c-Bak-SAS cells inoculated ASID mice tissue samples.jpg]

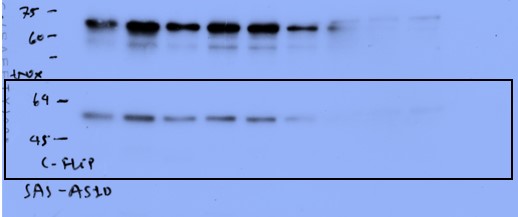

Supplement: Figure 10—source data 1. [file elife-87873-fig10-data1.zip › Figure 10-source data 1/figure 10c-Flip-SAS cells inoculated ASID mice tissue samples.jpg]

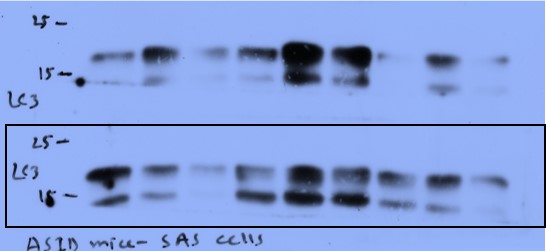

Supplement: Figure 10—source data 1. [file elife-87873-fig10-data1.zip › Figure 10-source data 1/figure 10c-LC3-SAS cells inoculated ASID mice tissue samples.jpg]

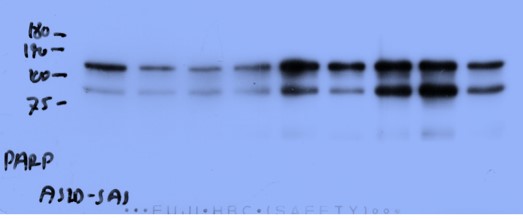

Supplement: Figure 10—source data 1. [file elife-87873-fig10-data1.zip › Figure 10-source data 1/figure 10c-PARP-SAS cells inoculates ASID mice tissue samples.jpg]

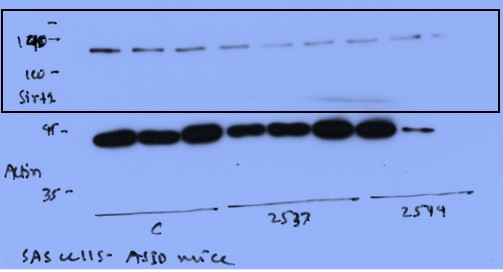

Supplement: Figure 10—source data 1. [file elife-87873-fig10-data1.zip › Figure 10-source data 1/figure 10c-SIRT1-SAS cells inoculated ASID mice tissue samples.jpg]

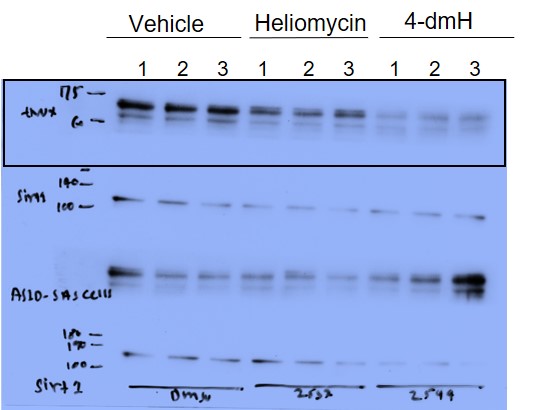

Supplement: Figure 10—source data 1. [file elife-87873-fig10-data1.zip › Figure 10-source data 1/Figure 10c-tNOX-SAS cells ASID mice tissue samples.jpg]

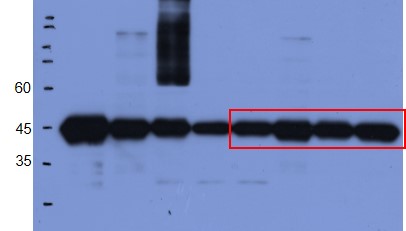

Supplement: Figure 10—source data 1. [file elife-87873-fig10-data1.zip › Figure 10-source data 1/Figure 10d -Actin-tongue.jpg]

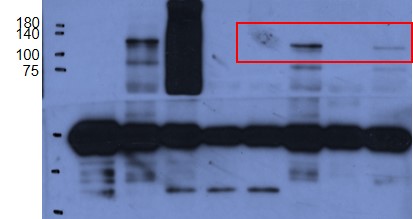

Supplement: Figure 10—source data 1. [file elife-87873-fig10-data1.zip › Figure 10-source data 1/Figure 10d -SIRT1-tongue.jpg]

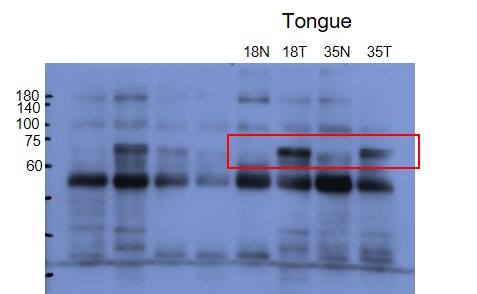

Supplement: Figure 10—source data 1. [file elife-87873-fig10-data1.zip › Figure 10-source data 1/Figure 10d -tNOX-tongue.jpg]

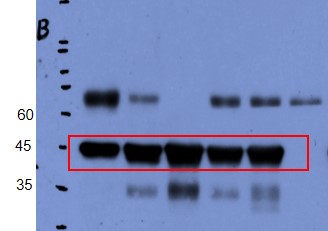

Supplement: Figure 10—source data 1. [file elife-87873-fig10-data1.zip › Figure 10-source data 1/Figure 10d-Actin-Buccal.jpg]

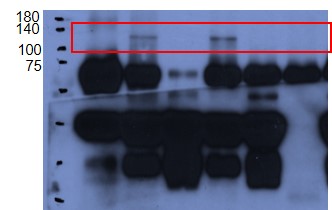

Supplement: Figure 10—source data 1. [file elife-87873-fig10-data1.zip › Figure 10-source data 1/Figure 10d-SIRT1-Buccal.jpg]

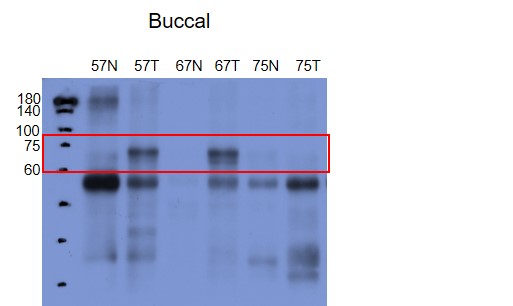

Supplement: Figure 10—source data 1. [file elife-87873-fig10-data1.zip › Figure 10-source data 1/Figure 10d-tNOX-Buccal.jpg]

Figure 10

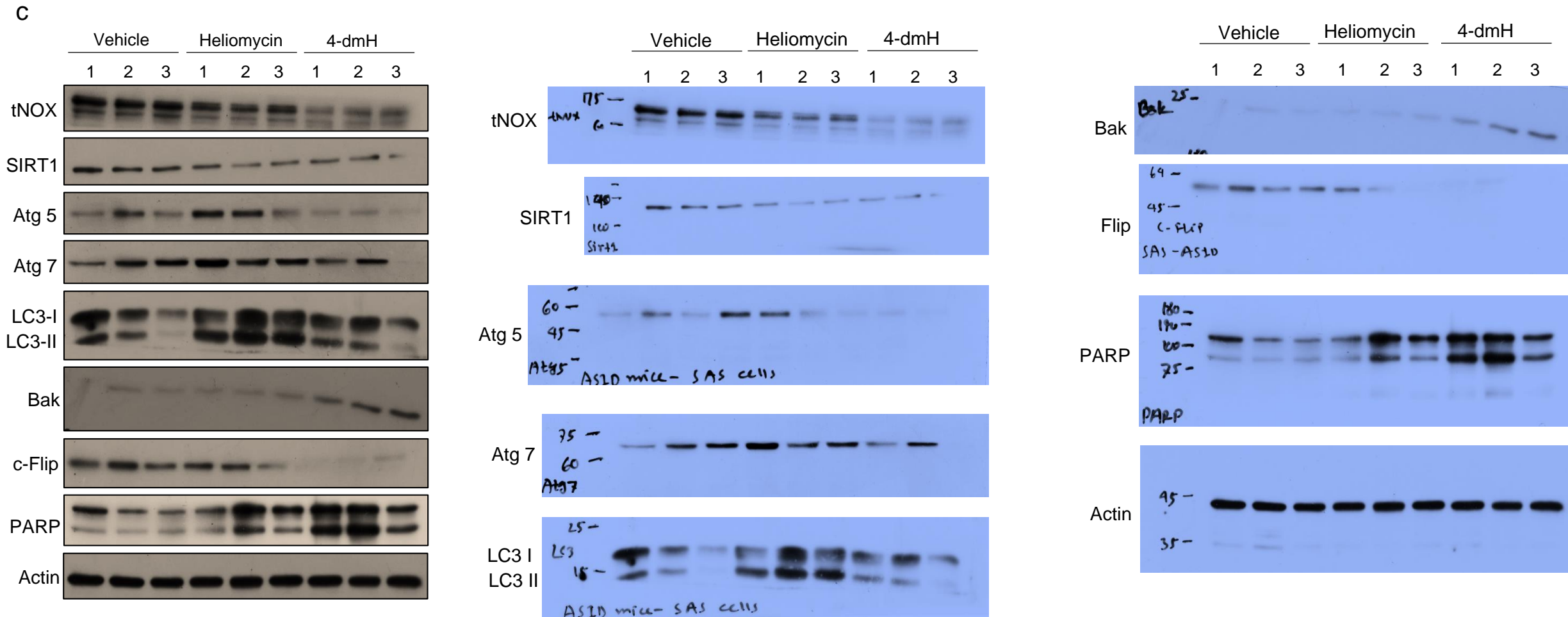

Figure 10

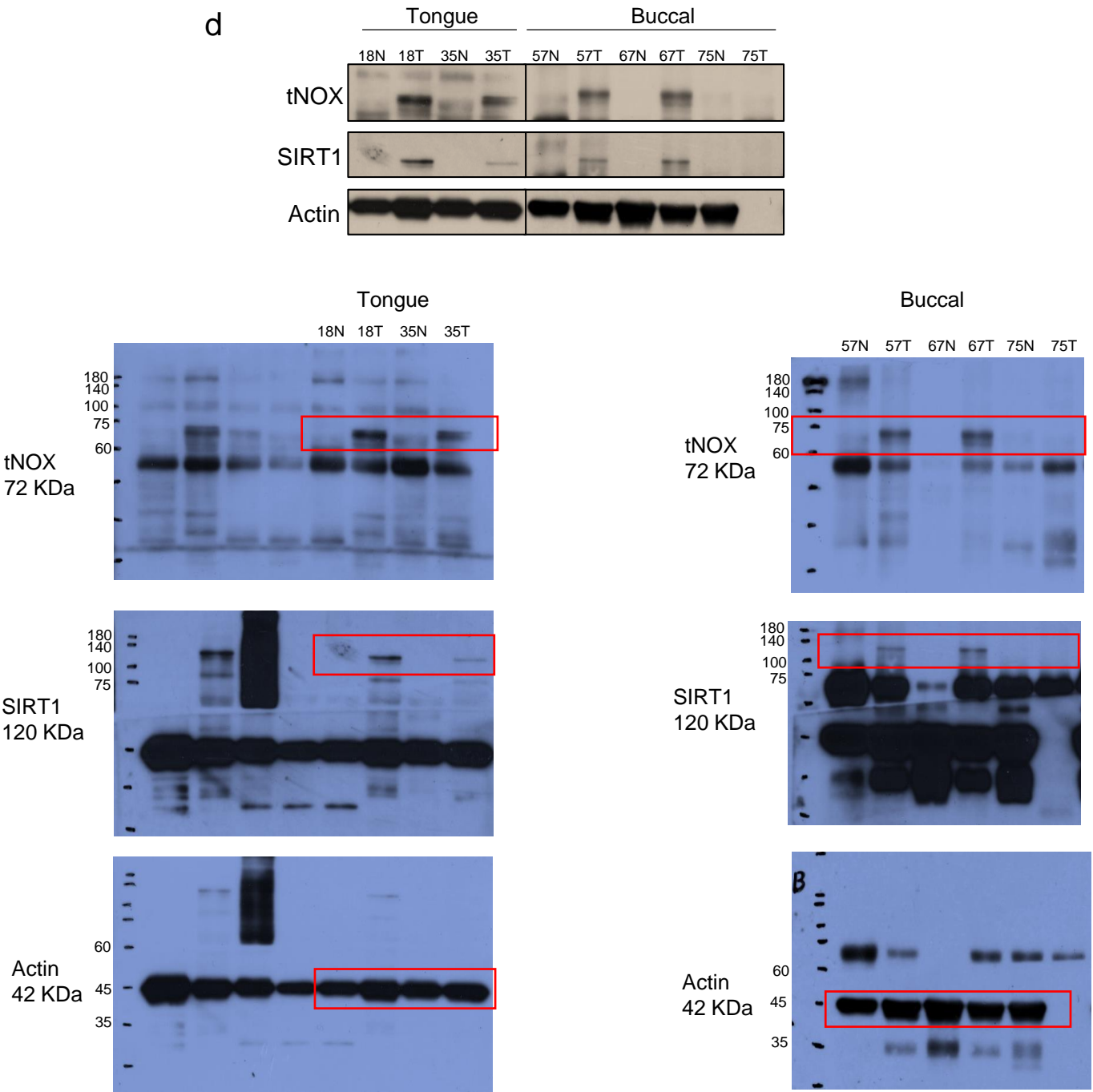

Supplement: Figure 10—source data 2. [file elife-87873-fig10-data2.zip › Figure 10-source data 2.pdf]
